# Outlearning extortioners: unbending strategies can foster reciprocal fairness and cooperation

**DOI:** 10.1093/pnasnexus/pgad176

**Published:** 2023-05-25

**Authors:** Xingru Chen, Feng Fu

**Affiliations:** School of Science, Beijing University of Posts and Telecommunications, Beijing 100876, China; Department of Mathematics, Dartmouth College, Hanover, 03755 NH, USA; Department of Mathematics, Dartmouth College, Hanover, 03755 NH, USA; Department of Biomedical Data Science, Geisel School of Medicine at Dartmouth, Lebanon, 03756 NH, USA

**Keywords:** evolutionary game theory, repeated games, cooperation, reciprocity, fairness

## Abstract

Recent theory shows that extortioners taking advantage of the zero-determinant (ZD) strategy can unilaterally claim an unfair share of the payoffs in the Iterated Prisoner’s Dilemma. It is thus suggested that against a fixed extortioner, any adapting coplayer should be subdued with full cooperation as their best response. In contrast, recent experiments demonstrate that human players often choose not to accede to extortion out of concern for fairness, actually causing extortioners to suffer more loss than themselves. In light of this, here we reveal fair-minded strategies that are *unbending* to extortion such that any payoff-maximizing extortioner ultimately will concede in their own interest by offering a fair split in head-to-head matches. We find and characterize multiple general classes of such unbending strategies, including generous ZD strategies and Win-Stay, Lose-Shift (WSLS) as particular examples. When against fixed unbending players, extortioners are forced with consequentially increasing losses whenever intending to demand a more unfair share. Our analysis also pivots to the importance of payoff structure in determining the superiority of ZD strategies and in particular their extortion ability. We show that an extortionate ZD player can be even outperformed by, for example, WSLS, if the total payoff of unilateral cooperation is smaller than that of mutual defection. Unbending strategies can be used to outlearn evolutionary extortioners and catalyze the evolution of Tit-for-Tat-like strategies out of ZD players. Our work has implications for promoting fairness and resisting extortion so as to uphold a just and cooperative society.

**Significance Statement**

Extortioners witting of the zero-determinant strategy can gain the upper hand in Iterated Prisoner’s Dilemma games by unilaterally enforcing an unfair linear relation between their own payoff and that of their coplayer. Therefore, theory predicts that acceding to extortion is the best response for any adapting coplayer. Recent empirical evidence, however, shows that human players seldom yield to extortion out of concern for fairness and are willing to discipline extortioners by refusing to fully cooperate. To shed light on such fair-minded responses, here we find and characterize general classes of unbending strategies such that the best response of any payoff-maximizing extortioner against a fixed unbending player is to offer a fair split, thereby guaranteeing equal pay for both parties.

## Introduction

The Prisoner’s Dilemma (PD) has been considered a central paradigm for understanding a wide variety of cooperation problems (1). In this game, two players decide whether to cooperate (C) or defect (D). If both players choose to cooperate, they receive the same reward for mutual cooperation, *R*, and if they both defect, they receive the same punishment for mutual defection, *P*. However, if one cooperates but the other defects, the defector receives the temptation to defect, *T*, whereas the cooperator receives the sucker’s payoff, *S*. These payoff values satisfy T>R>P>S (2).

Departure from one-shot games, the dynamics of the Iterated Prisoner’s Dilemma (IPD) can be analyzed by examining the four possible outcomes that arise when two players simultaneously play the game at each time step: (C,C), (D,D), (D,C), and (C,D). The former two outcomes result in equal payoffs for both players, while the latter two create a payoff inequality, with one player receiving a higher payoff than the other. In repeated interactions, it is possible for both players to have equal long-term average payoffs, or for one player to receive a higher payoff than the other.

To shed light on a range of cooperative or exploitative strategies in IPD games (3), prior studies have extensively investigated various behavioral choices and responses that can be characterized by prescribed intentions or preferences to be fair, cooperative, reciprocal, generous, and forgiving (or the opposite). These concepts involving reciprocal fairness and cooperation (also known as direct reciprocity, put it simply, “I will if you will”) can be investigated within the framework of IPD games.

Various strategies can be employed in an IPD game, with some being more cooperative and fair-minded than others. For instance, a fair-minded reciprocator would reciprocate cooperation at least as often as their coplayer does, rather than seeking an advantage over them. Among the common IPD strategies, Tit-for-Tat (TFT) and its variants, such as generous TFT (GTFT), are cooperative and fair-minded in nature (4). TFT-like players do not defect initially unless their coplayers had defected once or more. On the other hand, adaptive learning strategies (5), such as Win-Stay, Lose-Shift (WSLS), are more robust to noise and error than TFT (6). WSLS deterministically keeps the current strategy if the resulting payoff is above a fixed aspiration level, or switches otherwise.

An “equalizer” is capable of unilaterally setting any coplayer’s payoff level to the same arbitrary level within the range of [P,R] (7). Even more capable of bilateral payoff control is the zero-determinant (ZD) strategy, discovered by Press and Dyson (8). A ZD player can unilaterally set a linear relation between the payoff of themselves and that of the coplayer, regardless of the strategy of the coplayer. In recent years, the discovery of ZD strategies has generated renewed interest in studying IPD games in light of Press and Dyson’s finding (9–18).

Of particular interest is the existence of a continuous spectrum of ZD strategies that vary in their level of generosity, ranging from extortionate ZD to generous ZD (19). Undoubtedly, witting of ZD strategies enables players to gain the upper hand in IPD games, even allowing an implicit form of extortion (8). Self-serving extortioners can leverage ZD strategies to their advantage to the fullest extent, aiming to dominate any coplayer preemptively. However, it is shown that two extortioners, both equipped with the knowledge of extortionate ZD, will neutralize each other in their interactions and lead to their own demise, both receiving *P* (8). The lack of mutual cooperation among extortionate ZD players may prevent them from being favored by natural selection in an evolutionary population dynamics setting, particularly in larger populations (14). However, ZD players can still be successful in small populations, and even more so when they either adapt to be more generous towards others (19) or establish reconciliation and cooperation among themselves (20).

Prior work almost invariably considers ZD fixed, while their coplayer tries to adapt to ZD’s unilateral control. In reality, extortion can be met with resistance; *unbending* individuals are willing to push back any attempt to extort out of concern for fairness (10, 15, 21). Indeed, recent experiments demonstrate that fixed computer ZD players are able to outcompete their human counterparts but at a huge cost in a way that human players are significantly less cooperative (15). In a variety of experimental scenarios involving incentives for extortionate human players to receive additional bonuses based on their competitiveness (10), unbending players may give up their disciplinary efforts against extortioners, ultimately losing to them. Nevertheless, these unbending players can still sabotage the extortioners’ success through occasional defections, causing a decline in the extortioners’ payoffs compared to other control conditions (10). Thus, the success of ZD’s extortion attempt can be undermined and become less effective in reaching the fullest possible extent. Moreover, ZD players need to prescribe their strategies in a sophisticated way that explicitly depends on the underlying payoff matrix in the first place. It remains unknown how potential variations in the payoff matrix, which can arise from the uncertainty of evolving game environment (22), will impact the pairwise dominance of ZD strategies and in particular their extortion ability, since not all PD games are qualitatively the same (23).

These considerations lead us to reveal the previously unforeseen Achilles’ heel of ZD strategies, specifically in one-on-one encounters. Namely, there exist simple strategies (including TFT-like strategies and WSLS as particular examples) that are unbending to extortion and can cause an unfair demand to backfire on extortioners. When against a fixed unbending player, the best response of any payoff-maximizing extortioner, characterized by their prescribed smallest possible level of generosity, is to offer a fair split, thereby guaranteeing equal payoffs for both parties.

Moreover, we show that in interactions of more adversarial nature (24), characterized by the payoff structure condition T+S<2P, ZD’s dominance is drastically impaired, and extortioners tempting to dominate the coplayer are likely to become victims of their own success. The fixed unbending strategies, discovered in the present study, are able to not only force greedy ZD coplayers to be fair in their own interest but also more importantly, steer adapting coplayers (including those ZD coplayers) towards fairness and cooperation in adaptive learning settings. Our work provides useful insights into understanding the important role played by unbending strategies as an enforcer and stabilizer of fairness and cooperation in dyadic interactions, of relevance and interest for studying direct reciprocity.

## Results

We begin with studying the effectiveness of ZD strategies in payoff control and extortion and how it depends on their prescribed parameter choices and the underlying payoff matrix. Doing so will provide a new perspective on understanding specific conditions required for intended extortion to be successful or lack thereof. These critical considerations ultimately lead us to reveal unbending strategies that are able to outlearn ZD players and foster fairness and cooperation in pairwise interactions (see Figs. S1–S15 and Tables S1–S7 in the Online Supplementary Material).

Following common practice (6), we denote memory-one IPD strategies by $p=[p_{1},p_{2},p_{3},p_{4}]$, where $p_{i}$, for i=1,…,4, is the conditional probability to cooperate, respectively, after experiencing one of the four possible outcomes each round {CC,CD,DC,DD}, that is, written from the perspective of a focal player X (the first letter represents X’s last move, and the second letter for the coplayer Y’s). Suppose that player X uses a ZD strategy p and the coplayer Y uses an arbitrary strategy $q=[q_{1},q_{2},q_{3},q_{4}]$, and let $s_{X}$ denote the average payoff of player X and $s_{Y}$ that of player Y. A general yet intuitive parameterization of memory-one ZD strategies are based on three control parameters (O,χ′,ϕ):


(1) $$\left\{ \begin{array}{l} p_{1}=1-\phi (R-O)(\chi \prime -1)\;,\\ p_{2}=1-\phi [(T-O)\chi \prime +(O-S)],\\ p_{3}=\phi [(O-S)\chi \prime +(T-O)],\\ p_{4}=\phi (O-P)(\chi \prime -1), \end{array}\right.$$

including the extortion factor χ′>1, the baseline payoff O∈[P,R], and the normalization factor ***ϕ*** that ensures p to be a proper probability vector. A complete discussion of the admissible ranges of these parameters can be found in the Online Supplementary Material.

**Fig. 1.**
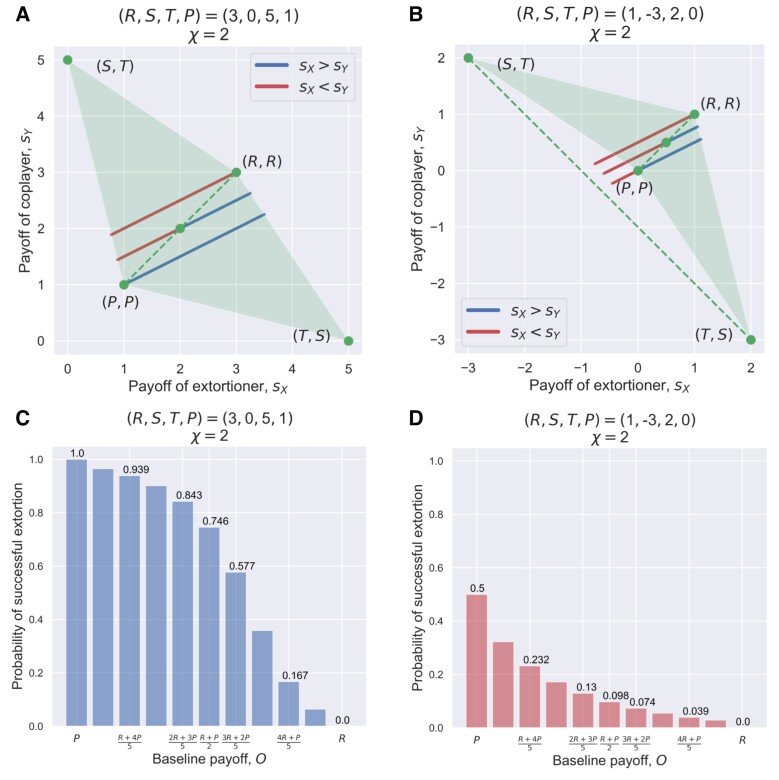
Pairwise dominance and extortion ability of ZD strategies. The baseline payoff *O* used by the ZD player X is regarded as extortionate *P* (least level of generosity), generous *R* (maximum level of generosity), and in between (P+R)/2 (intermediate level of generosity). The optimality of extortionate ZD strategies (with O=P) nontrivially depends on both the strategy of their coplayer and the payoff structure. When playing against a certain type of coplayers (which we call unbending strategies), extortioners can maximize their prospective payoffs only if aiming for an equal split by letting the extortion factor χ′→1. Moreover, when T+S<2P, extortioners can even be outperformed. In panels a) and b), we show the scatter plot of payoff pairs $\left(s_{X},s_{Y}\right)$ of ZD players against random coplayers uniformly drawn from all possible memory-one strategies $[0,1{]}^{4}$ in a) T+S>2P and in b) T+S<2P. Shown in c) and d) is the probability that a ZD player X actually gets better payoff than their coplayer Y ($s_{X}>s_{Y}$) who uses a random strategy uniformly drawn from memory-one strategies $[0,1{]}^{4}$, with respect to varying their baseline payoff O∈[P,R]. The parameter *O* controls the level of generosity of a ZD player but also impacts their chance to outperform their coplayers (“extortion ability”). Increasing *O* above *P* makes ZD less likely to be able to ensure the dominance over their coplayers. Noticeably, the payoff structure plays an even more pronounced role than does the parameter *O*: c) for T+S>2P the curvature is concave downward and ZD is able to maintain dominance for most of the time even using intermediate O>P values, d) whereas concave upward for T+S>2P and ZD is more likely to lose dominance for any P<O≤R. In line with a) and b), extortion with O=P always leads to superior payoff than the coplayers a) when T+S>2P, but not necessarily true for b) T+S<2P. Parameters: a–d) ZD player X’s extortion factor χ′=2, and ***ϕ*** is uniformly distributed and truncated at the admissible upper bound, a,c) R=3, S=0, T=5, P=1, b,d) R=1, S=−3, T=2, P=0.

Regardless of the strategy q used by Y, X unilaterally enforces a linear relative payoff relation of the form (8, 19):


(2) $$s_{X}-O=\chi \prime (s_{Y}-O),$$

which represents a straight line in the parametric plot of $\left(s_{X},s_{Y}\right)$ with the slope 1/χ′ (the reciprocal of the extortion factor χ) (Fig. 1a and b). In this plane, the baseline payoff O∈[P,R] determines the intercept to the line of equal payoffs, $s_{X}=s_{Y}$, and also dictates the level of generosity (19).

The payoff control as given in Eq. 2 enables an implicit form of extortion where ZD player X can prescribe their strategies in a way that they reciprocate cooperation less frequently than their coplayer Y (8, 14). For ZD players, the way to attempt such dominance and extortion is to deliberately choose their parameters *O* and χ in advance, which will in turn determine admissible values of ***ϕ***. The chosen values of *O* and χ can be observed directly from pairwise payoff plots (Fig. 1a and b), and together with the underlying payoff matrix, they jointly determine the upper bound of admissible ***ϕ*** values. For example, a widely used parameterization of this ZD class, which is called extortionate ZD strategy (8), ensures that $s_{X}-P=\chi \prime (s_{Y}-P)$ holds with χ′>1. The admissible range of ***ϕ*** for extortionate ZD is given by


(3) $$0<\phi \leq \phi^{upper}=\left\{ \begin{array}{cc} \frac{1}{\left(T-P)\chi \prime \;+\;(P-S\right)}, & T+S\geq 2P.\\ \frac{1}{\left(P-S)\chi \prime \;+\;(T-P\right)}, & T+S<2P. \end{array}\right.$$

Notably, the parameter ***ϕ*** has an upper bound that explicitly depends on the sign of T+S−2P. We emphasize that this previously overlooked payoff structure condition, whether T+S>2P holds or not, surprisingly strikes out as an important condition for determining the optimality of ZD strategies and their extortion ability (see Fig. S16 in the Online Supplementary Material). As shown in Fig. 1c and d, as long as a ZD player uses the minimal O=P and χ′>1, they secure the most favorable position to dominate and get higher payoffs than their opponent as compared to other *O* values, regardless of the underlying payoff matrix (Fig. 1c and d). Despite such a contextual difference of ZD’s extortion ability owing to the change in the underlying payoff structure, we still refer to this class of “extortionate ZD” as extortioner as in Ref. (14), for the sake of consistency. First, the effect of varying their control parameter *O* on their resulting level of generosity and extortion ability remains qualitatively consistent across IPD games of drastically different nature. Second, for ZD players with O=P, no matter what types of IPD games they are engaged in, the chosen value of *P* characterizes the least level of generosity, and thus preemptively sets their extortion ability at maximum, even though these so-called extortioners will not always succeed in securing advantage as intended, particularly when T+S<2P (cf. Fig. 1c and d).

**Fig. 2.**
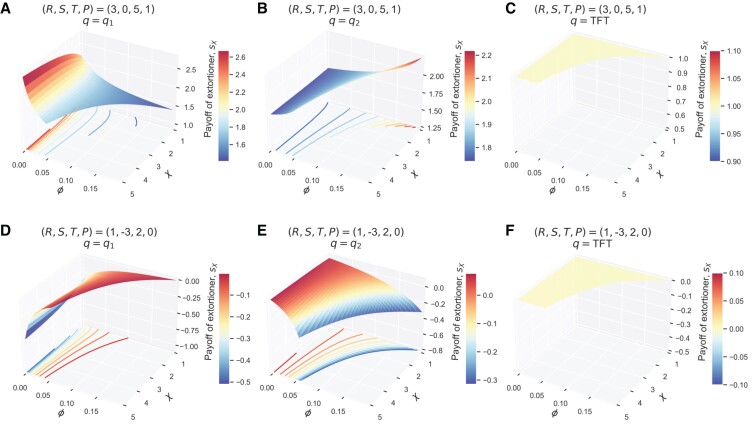
Impacts of control parameters (ϕ,χ′) on the average payoff of a ZD player when playing against a fixed coplayer. The ZD player X’s payoff, $s_{X}$, is shown as a function of the normalization factor ***ϕ*** and the extortion factor χ, along with contour lines projected on the (ϕ,χ′)-plane: a–c) for T+S>2P and d–f) for T+S<2P. The ZD player X’s payoff is either monotonic or remains constant with respect to ***ϕ*** while it can exhibit nonmonotonic behavior with respect to χ. Despite being able to enforce a linear payoff relationship $s_{X}-P=\chi \prime (s_{Y}-P)$, ZD player X that unilaterally uses a larger extortion factor χ does not necessarily lead to further payoff gains as demonstrated in a). As long as “winning isn’t everything” (actual payoff performance is concerned in wide-ranging scenarios), ZD can subtly tune their control parameters to optimize their own payoff performance against a fixed coplayer. Parameters: a–f): X uses the most formidable ZD strategy with O=P (also known as extortionate ZD), a–c) R=3, S=0, T=5, P=1, the upper bound of ϕ=1/[(T−P)χ′+(P−S)], d–f) R=1, S=−3, T=2, P=0, the upper bound of ϕ=1/[(P−S)χ′+(T−P)], coplayer Y’s strategy $q_{1}=[0.05,0.95,0.05,0.1]$, $q_{2}=[0.4,0.1,0.9,0.2]$, $q_{3}=\text{TFT}=[1,0,1,0]$.

On the other hand, given the uncertainty of vastly possible strategies the coplayer could use against ZD players, it is worthwhile to quantify the robustness of the dominance and the performance of ZD strategies with particular respect to varying their baseline payoff *O*. It is likely that ZD players choose O=P+ε deviating from *P* for plausible reasons like the trembling hand (25) or “blurred minds” (4), and as a consequence, they will respond with nonzero cooperation (i.e. $p_{4}>0$) after entering mutual defection state with their coplayer. Even so, any ZD player using O<R still has extortion ability to some extent unless they use the generous ZD with O=R that ensures their average payoffs are never above the coplayer’s (19) (Fig. 1). It is thus reasonable to consider the extortion ability of ZD strategies as a continuous spectrum—“the likelihood of getting better payoffs than any kind of opponent”—instead of a binary character (either always or not at all). In doing so, we are able to quantify and compare the extortion ability of ZD strategies and how it depends on their control parameters (O,χ′,ϕ), and more remarkably, on the underlying payoff structure specified by the sign of T+S−2P (Figs. 1 and 2).

We further note that ***ϕ*** is a hidden parameter, which has received little attention in prior studies. However, we find that albeit the normalization factor ***ϕ*** has no impact on the linear payoff relation, it can nontrivially affect the average payoff values that a ZD player will receive (Fig. 2). Mathematically, ZD’s average payoff $s_{X}$ is given by the ratio of the determinants of two matrices, giving rise to a rational function (8). We can show that $s_{X}$ is a monotonic function of ***ϕ*** (Figs. S17 and S18 in the Online Supplementary Material) but can have strict nonmonotonicity with respect to χ, exhibiting as a one-humped function of χ (see Online Supplementary Material for derivation details). Fig. 2 plots an extortionate ZD’s average payoff $s_{X}$ (with the baseline payoff O=P) against a fixed coplayer Y using a specific strategy as a function of the parameter space (ϕ,χ′). This result further demonstrates that ZD can unilaterally fine tune their control parameters, in particular the previously overlooked parameter ***ϕ*** to their own advantage (which would be boundary values of its admissible interval, either infinitely small or the upper bound).

Only if T+S>2P is an extortionate ZD unbeatable, ensuring no less payoffs than their opponent (the worst scenario is a tie, e.g. against TFT as shown in Fig. 2c and f). In this case, making the extortion factor χ excessively larger surely can help ZD impose a greater relative advantage over their opponent, but their actual average payoff can be seriously comprised (Fig. 2a). Even worse, when T+S<2P, $s_{X}$ can drop below *P* and due to $s_{X}-P=\chi \prime (s_{Y}-P)$ we have $s_{X}<s_{Y}<P$ (Fig. 2e). In accordance with Fig. 1, the payoff structure can completely change the impact of varying ***ϕ*** and χ on ZD’s performance (cf. Fig. 2a and d, cf. 2b and e). This is one of the novel insights stemming from the present study, complementing the prior finding that ZD strategies are disfavored in population dynamics settings (14, 19, 20). Altogether, these results are key to improving our understanding of previously unforeseen limitations of ZD strategies in head-to-head matches in IPD games.

When an individual is knowingly confronted with extortion and especially has known the limitations of ZD strategies (Figs. 1 and 2), should this player be subdued or otherwise unbending? Prior work demonstrates that if an individual accedes by fully cooperating with an extortioner who fixes their strategies, both their payoffs are maximized (Fig. 1a and b). Conversely, here we ask whether there exist *unbending* players who choose to fix their strategies such that extortioners could maximize their payoffs only if they try to be fair by letting χ′→1. Otherwise, extortioners would have experienced a decline in their average payoffs if they ever demanded an unequal share by increasing χ.

Motivated by these, we further explore unbending strategies that are able to force adapting ZD strategies, among these least generous ones with O=P and hence equipped with the greatest level of extortion ability, to offer a fair split by letting χ′→1 in their own interest and guarantee equal pay for both sides. Considering that any ZD player can always modulate their hidden parameter ***ϕ*** to extreme values to favor their gains in the interactions (Fig. 2), we suppose unbending strategies, without loss of generality, will need to (i) neutralize the parameter ***ϕ*** in the first place such that both of their average payoffs are independent of ***ϕ***, $\partial s_{X}/\partial \phi =0$, and (ii) guarantee that the derivative of $s_{X}$ with respect to χ is strictly negative, $\partial s_{X}/\partial \chi \prime <0$.

These required properties of unbending strategies lead us to search and identify general classes of strategy candidates that can counteract the adversary imposed by extortioners, provided that they can trigger the backfire of being extortionate. To put it simply, when confronted with a fixed unbending player, any extortionate ZD player is disciplined with payoff reductions in the way that a higher degree of extortion leads to a smaller average payoff. Here, we simplify the interaction process by assuming targeted interactions between an unbending player and an extortionate ZD coplayer, without requiring the recognition and assessment of the possibility of coplayer’s extortion as discussed in Ref. (26). However, we later relax this assumption during our study of steering learning dynamics of adaptive players to consider more general strategies beyond extortionate ZD.

Thus, a potential candidate q of unbending strategies outlearning any extortionate ZD coplayer p needs to mitigate the impact of χ and ***ϕ***, which are unilaterally controlled by the extortioner. To this end, we find that four classes of unbending strategies $q=[q_{1},q_{2},q_{3},q_{4}]$ that can make their average payoffs independent of ***ϕ*** (as detailed in the Online Supplementary Material):

**Table:** 

Class A:	$q_{1}=1$ and $q_{3}=0$	Class B:	$q_{2}=q_{3}=0$
Class C:	$q_{1}=q_{2}=q_{3}$	Class D:	$q_{4}=h_{D}(q_{1},q_{2},q_{3})$

Here in class D, $h_{D}=[T-R-P+S-(T+S-2P)q_{1}+(R-P)(q_{2}+q_{3})]/(2R-T-S)$, which is exactly the same linear relation satisfied by any ZD strategy. Class B only exists when T+S<2P and the maximum payoffs for both sides can receive is *R* (which is an equal-pay outcome). Class C contains “willing” [1,1,1,0] on the boundary (27), against which an extortioner can only maximize their own payoffs by being fair (χ′→1), thereby ensuring equal payoffs ($s_{X}=s_{Y}\to R$) with unbending “willing” (1−δ,1−δ,1−δ,ε) for δ→0, and ε→0 (Table S12 in the Online Supplementary Material). The complete analysis and discussion of these two classes B and C can be found in the Online Supplementary Material.

**Fig. 3.**
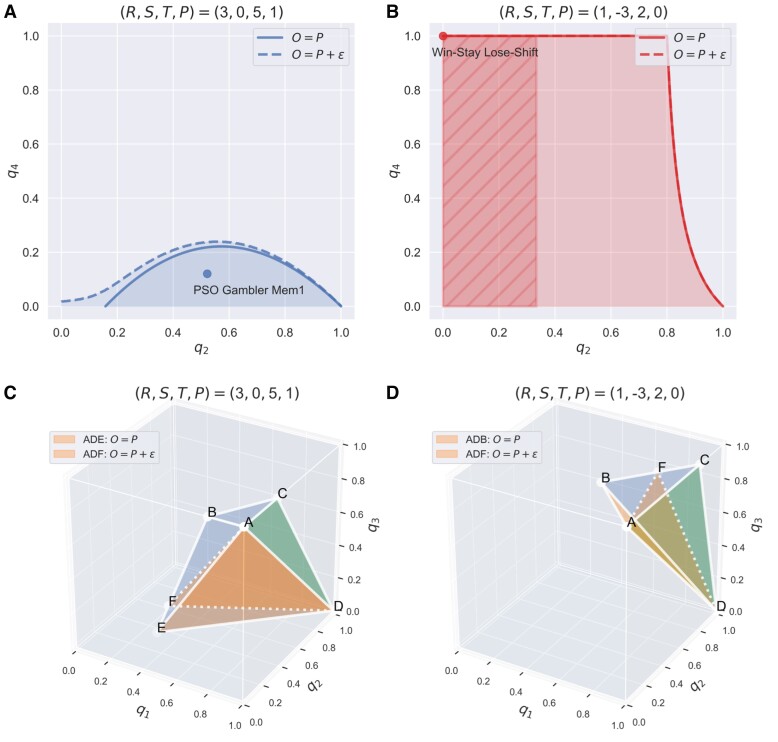
Revealing strategies that are *unbending* to extortioners in IPD games. Shown is the strategy space of unbending players that are able to cause the monotonic decrease of an extortionate ZD (parameterized with O=P, namely, the least generous type regardless of the sign T+S−2P) player’s payoff with respect to the extortion factor χ. Extortioners can demand an even more unfair share by unilaterally raising their extortion factor χ. However, an unexpected drop in their prospective payoffs if intentionally being more extortionate is likely to compel self-interested extortioners who want to maximize their payoffs to be fair. In this sense, unbending strategies can be used to steer their coplayers from extortion to fairness. The strategy space of unbending players depends on the sign of T+S−2P, and we show two general classes of interest (see Online Supplementary Material for the complete classification): one class has the form $\left[1,q_{2},0,q_{4}\right]$ with combinations of $q_{2}$ and $q_{4}$ shown in a) and b), and another class has the form $\left[q_{1},q_{2},q_{3},q_{4}\right]$ where $q_{4}=[T-R-P+S-(T+S-2P)q_{1}+(R-P)(q_{2}+q_{3})]/(2R-T-S)$. This latter class in fact contains all ZD strategies that enforce a linear payoff relation $s_{X}-O=\chi \prime (s_{Y}-O)$ with O>P. Particular examples of unbending strategies include a) the memory-one PSO Gambler which is optimized by using particle swarm algorithms, b) WSLS, and c, d) all ZD strategies with O>P. The dashed lines show the altered boundary of unbending strategies against the ZD player X using O=P+ε as opposed to O=P; the region of unbending strategies for class A (T+S<2P) remains *unchanged* as shown in panel (b). The shaded area in b) shows the region where ZD player X, even though using minimal O=P, can be outperformed by unbending strategies (see Table S9 in the Online Supplementary Material for details). This result is in line with Fig. 1, which shows that the payoff structure T+S<2P drastically hinders a ZD player’s ability to extort and dominate their coplayers, let alone those unbending ones. Parameters: a, b) R=3, S=0, T=5, P=1, ε=0.05; c, d) R=1, S=−3, T=2, P=0, ε=0.5.

Furthermore, the entire strategy space comprised of all admissible unbending strategies can be characterized by requiring the derivative $\partial s_{X}/\partial \chi \prime <0$ (Fig. 3). Again, the sign of T+S−2P determines the geometry of the strategy space satisfying unbending properties (see Fig. 3a and b for class A, Fig. 3c and d for class D). Of particular interest, the memory-one particle swarm optimization (PSO) Gambler q=[1,0.5217,0,0.1205], an optimized strategy using PSO algorithms in IPD games with the conventional payoff values (13), belongs to class A of unbending strategies (Fig. 3a), and WSLS is an unbending strategy only if T+S<2P (Fig. 3b).

Interestingly and coincidentally, we find that all ZD strategies with O>P and χ′>1 are unbending to extortionate ZD (Fig. 3c and d). It is worth noting that these planes specifying the boundary of class D have particular meanings. As shown in Fig. 3c, the shaded triangle ADE represents the set of extortionate ZD strategies with O=P and χ′>1, and the shaded area by the four-sided polygon BCDE represents the set of equalizer strategies, and all unbending strategies in class D are in between these two planes and bounded by the unit cube. Besides, the triangle ACD represents the set of generous ZD strategies with O=R, and the triangle ABD represents the set of ZD strategies with O=(T+S)/2. For T+S<2P (Fig. 3d), the strategy space degenerates into the region between the triangle ABD (extortionate ZD) and triangle BCD (equalizer). Hence, we conclude that class D contains all ZD strategies with O>P.

We also have extended our search of fixed unbending strategies with respect to an even broader class of ZD strategies just with positive χ′>1 (namely, using the baseline payoff O=P+ε≥P and still having extortion ability to some degree as shown in Fig. 1c and d), such that ZD’s payoff is independent of the normalization factor ***ϕ*** and monotonically decreases with their extortion factor χ. As shown in Fig. 3 (highlighted with dashed lines) and the Online Supplementary Material, our classification of unbending strategies (especially nontrivial classes A and D) remains largely robust with respect to this important extension. Unexpectedly, we also find a set of nonlinear memory-one (non-ZD) strategies, when having the knowledge of the ZD coplayer’s baseline payoff *O*, will always be able to ensure equal payoffs *O* for both (see Fig. S21 and Table S27 in the Online Supplementary Material for details).

To provide further intuition about why extortion against unbending players fails to yield better average payoffs, we consider the case where an extortioner *X* with (P,χ′,ϕ) plays against a fixed generous ZD player Y with (R,χ′,ϕ) which in fact belongs to class D of unbending strategies. Both of their resulting payoffs are *independent* of their ***ϕ*** values, and the extortioner *X* has an average payoff given by


(4) $$s_{X}(\chi \prime )=\frac{P(\chi \prime -1)+R\chi \prime (\chi \prime^{\prime }-1)}{\chi \prime \chi \prime^{\prime }-1}.$$

**Fig. 4.**
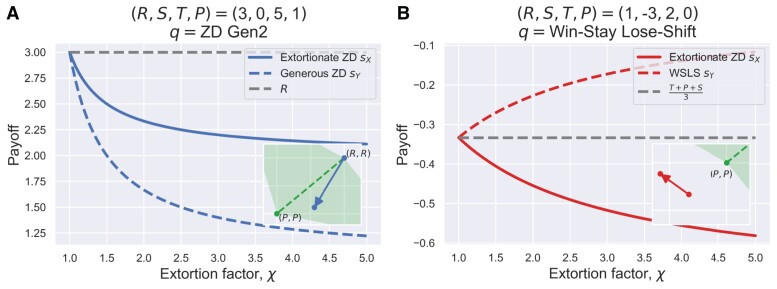
Intuition for how unfair demand can backfire on extortioners. As shown in panel a), despite being able to enforce payoff control against generous ZD (with χ′=2), the prospective payoff of extortionate ZD monotonically decreases with their extortion factor χ. The unfair extortion backfires on ZD which intended to demand a higher proportion but ended up with less payoff than what they would have obtained if being fairer otherwise. Even more, extortioners are outperformed by WSLS, as shown in panel b) when T+S<2P. The payoffs of the extortioner and WSLS, $s_{X}$ and $s_{Y}$, are both less than *P*, but intended extortion inflicts the unprecedented opposite outcomes: extortioner suffers more, whereas WSLS gains more. Targeted at extortioners, unbending strategies can be used to foster fairness in IPD games. The inset plots in a) and b) show the zoomed-in view of the scatter plot of payoffs, in a fashion similar to Fig. 1a and b, with their arrows to indicate their directions of change starting from fair, equal split as ZD increases χ above one. Parameters: a) R=3, S=0, T=5, P=1, b) R=1, S=−3, T=2, P=0.

We see that $s_{X}(\chi \prime )$ is monotonically decreasing with χ, as the derivative $ds_{X}/d\chi \prime =-(R-P)(\chi \prime -1)/(\chi \prime \chi \prime -1{)}^{2}<0$ for R>P, χ′>1, and χ′>1 (Fig. 4a).

Geometrically visualizing this specific example, the generous ZD player Y enforces a linear payoff relation as $s_{Y}-R=\chi \prime (s_{X}-R)$, whereas the extortioner *X* enforces $s_{X}-P=\chi \prime (s_{Y}-P)$, and the resulting payoff pair $\left(s_{X},s_{Y}\right)$ lies in the intersection of these two straight lines. If the extortioner *X* increases the extortion factor χ, the intersection point will move down along the line of $s_{Y}-R=\chi \prime (s_{X}-R)$ (if the generous ZD player Y remains unchanged). Therefore, the more unfair demand towards a fixed generous ZD player, the less payoff extortion yields. This previously unforeseen “backfire” is self-inflicted by the attempt to extort. For a self-interested individual who cares about how much they get, not just about monopolizing control of relative payoff, it does not pay to extort a generous ZD coplayer, and unfair demand backfires on extortioners who would have received the maximum *R* if trying to be fair by setting χ′→1 (Fig. 4a).

We now turn to explain the intuition behind the payoff structure of IPD games that can impact the dominance (optimality) of ZD strategies. It is well known that the condition T+S<2R is needed for mutual cooperation to fare better than alternating C and D pairs in the IPD. Yet another condition T+S>2P comes into sight if one ponders the condition under which the average payoff of any IPD strategy cannot be worse than *P*, the payoff for ending up with the deadlock of mutual defection. IPD is typically studied using the conventional values R=3, S=0, T=5, P=1, satisfying 2P<T+S<2R, and thus it ensures the average payoff of any IPD strategy cannot be less than *P*. Extortionate ZD players attain payoff control and extortion as desired $s_{X}-P=\chi \prime (s_{Y}-P)$ in this scenario using the conventional payoff values (Fig. 1a and c), but the tide will turn against extortioners if the payoff structure satisfies T+S<2P. In this latter case, the average payoff of extortionate ZD strategies can be lower than *P* when facing off certain IPD strategies (Fig. 1b and d).

As ZD strategies are explicitly dependent on the underlying payoff matrix whose elements are (R,S,T,P), we discover that the particular payoff structure, which is governed by the sign of T+S−2P, can fundamentally change the dominance of extortionate ZD strategies (Fig. 1, also see Tables S8–S16 in the Online Supplementary Material). For example, when an extortionate ZD player is pitted against WSLS with q=[1,0,0,1], the stationary distribution v of pairwise outcomes {CC,CD,DC,DD} is, up to a positive normalization factor, given by


(5) $$v_{\text{CC}}=0,\;v_{\text{CD}}=\frac{T-P+\chi \prime (P-S)}{\chi \prime (T-P)+P-S},\;v_{\text{DC}}=1,\;v_{\text{DD}}=1.$$

Therefore, in order to gain an advantage, extortion ZD must ensure $v_{\text{CD}}<v_{\text{DC}}$. However, this condition cannot always be satisfied when T+S<2P (the shaded region in Fig. 3b, see Tables S9 and S13 in the Online Supplementary Material for details). On the contrary, the extortionate ZD player in fact reciprocates unilateral cooperation more frequently than WSLS if $v_{\text{CD}}>v_{\text{DC}}$ holds, which is equivalent to requiring T+S<2P. Under this payoff structure condition, WSLS outperforms any extortionate ZD player (Fig. 4b); the more greedy extortion, the more ZD loses. Noteworthy, there is absolutely no mutual cooperation between WSLS and extortionate ZD players. Extortionate ZD does not fully cooperate after a mutual cooperation move, and thus ZD and WSLS will eventually end up with mutual defection from which ZD will never respond with cooperation while WSLS will always respond with cooperation; they will never be back to mutual cooperation. As a consequence, in the long run, no mutual cooperation between them can be established at all.

**Fig. 5.**
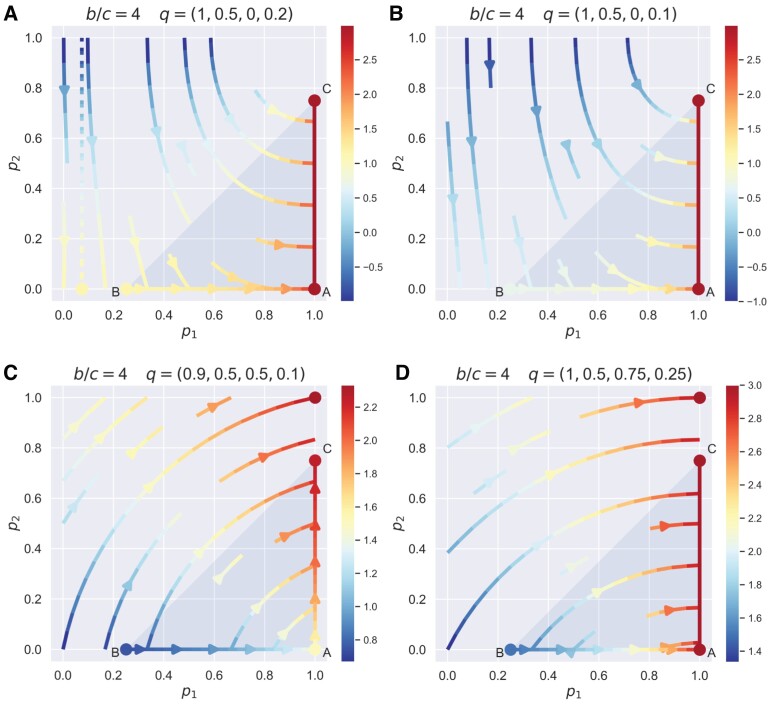
Steering learning dynamics towards fairness and cooperation with unbending strategies. Shown are the stream plots (vector fields) from the adaptive learning dynamics of a self-interested focal player X who uses a general reactive strategy $\left[p_{1},p_{2},p_{1},p_{2}\right]$ against a fixed unbending coplayer Y: a, b) from class A and c, d) from class D. Class A of unbending strategies are able to steer their coplayer X ultimately to behave like GTFT. Panel a) demonstrates that, depending on the specific unbending strategy player Y uses, the direction of change of player X’s $p_{1}$ can exhibit bistability (separated by the dashed line in (a)), which further depends on the initial state of X’s strategy. Panel b) shows that there exists a subset of class A that is able to direct the change of $p_{1}$ always towards full cooperation. In a, b), on the edge $p_{1}=1$, the direction of change of $p_{2}$ is neutral, and the line segment of AC indicates the set of compliers; on the line segment of the edge $p_{2}=0$, indicated by BA, which represents a subset of extortionate ZD players (extortioners), the learning dynamics of the ZD player X always converges to TFT (1,0). Panels c) and d) show that class D of unbending strategies are able to steer adaptive learning dynamics of X globally to the cooperative edge $p_{1}=1$ on which the direction of change of $p_{2}$ c) either is increasing if Y uses a strategy from class D that is an intermediate ZD with P<O<R) d) or remains neutral if Y uses a strategy from class D that happens to be the generous ZD with O=R. In both a) and b), the global maximum for X’s payoff is reached at the edge $p_{1}=1$ if $b/c>(\sqrt{5}+1)/2$, and in c) only at (1,1) does the global payoff maximum for X occur whereas so does the entire cooperative edge $p_{1}=1$ in d). The shaded triangle ABC in each panel indicates the part of reactive strategies which belong to the subset of general ZD strategies with positive χ. The color of the curves and dots corresponds to the payoff values of X in situ, as specified by the given colorbar. The PD game is parameterized using a donation game R=b−c, S=−c, T=b, P=0 with b=4 and c=1.

To further understand fixed unbending player’s unprecedented *steering* role in enforcing fairness and cooperation, we consider adaptive learning dynamics of a focal player *X* using a much broader space of strategies, rather than being limited to extortionate ZD, in a donation game which is a simplified PD (14, 19) (see Tables S17 and S18 in the Online Supplementary Material). Under this donation game satisfying the “equal gains from switching” (i.e. T+S=R+P), the memory-one reactive strategies $p=[p_{1},p_{2},p_{1},p_{2}]$ is actually a subset of ZD strategies (14) (also see Fig. S19 in the Online Supplementary Material). The shaded triangle in Fig. 5 indicates all such ZD strategies with positive χ′>1: BA represents extortionate ZD with χ′>1; point A is TFT with χ′→1 and O=P; point B is equalizer with O=P and χ′→∞; point C is GTFT with O=R and χ′→∞; BC represents the class of “equalizer” strategies. We find that if the benefit-to-cost ratio $b/c>(\sqrt{5}+1)/2$ (“golden ratio”), the cooperative edge $\left(1,p_{2}\right)$ is guaranteed to have the maximum average payoff value for player X when interacting with any fixed unbending player from class A (the region highlighted in Fig. 3a). Depending on the specific strategy of the unbending player Y from class A (see Fig. S20 and Table S23 for more details in the Online Supplementary Material), there could exist bistable learning outcomes of X’s final strategies: X can converge to the all defection corner (0,0) or otherwise to the cooperative edge $\left(1,p_{2}\right)$ (Fig. 5a), but there is a subset of class A of unbending strategies that ensures the global convergence to the cooperative edge $\left(1,p_{2}\right)$ (Fig. 5b, and Fig. S20, Tables S19–S22 in the Online Supplementary Material).

The steered learning dynamics under the influence of the coplayer Y from class D of unbending strategies is shown in Fig. 5c and d (also see Table S24 in the Online Supplementary Material). Generally speaking, the learning dynamics of a focal player X against class D of unbending strategies (in other words, ZD strategies with a higher level of generosity than player X) adds useful insights by complementing previous results in Ref. (8) that focuses on adapting coplayer Y against an extortionate ZD player X. Here, we show that the final strategy of player X converges to the cooperative edge, reaching full cooperation if against an unbending strategy from class D with O<R (still more generous than any extortionate ZD strategies on the edge BA with O=P) (Fig. 5c) or remains neutral on the cooperative edge once reaching there when against an unbending strategy from class D with O=R (generous ZD) (Fig. 5d).

In the Online Supplementary Material, we study the corresponding learning dynamics of a general ZD player within the parameter space (O,χ′) (see Tables S25 and S26 in the Online Supplementary Material) and confirm qualitatively similar results as reported here. In particular, when against a fixed unbending player, an adapting extortioner with the intended extortion factor χ unexpectedly suffers greater payoff reductions than their counterpart who chooses not to accede unless offering a fair split. For this reason, any evolutionary extortioner who aspires to maximize their own payoff will be compelled from extortion to fairness by adjusting their χ values. Since there is no interference by the parameter ***ϕ*** as $\partial s_{X}(\chi \prime ,\phi )/\partial \phi =0$, such reactive learning dynamics of extortioners is governed solely by the evolution of χ towards payoff optimization:


(6) $$\frac{d\chi \prime }{dt}=\tau \frac{\partial s_{X}(\chi \prime ,\phi )}{\partial \chi \prime }<0,$$

where the properly chosen timescale parameter τ guarantees that the state of Markov chains of gameplay reaches equilibrium faster than the learning dynamics (see Online Supplementary Material).

Thus, a self-interested extortioner tends to adjust χ as small as possible and ultimately behaves like TFT by letting χ′→1, thereby guaranteeing equal payoffs for both parties (see the change of direction on the edge of O=P in Tables S25 and S26 in the Online Supplementary Material). In evolving populations, natural selection favors generosity over extortion (19), and in head-to-head matches as demonstrated here, players with the knowledge of unbending strategies can outlearn extortioners and foster fairness and reciprocity in dyadic interactions.

Noteworthy, in the aforementioned steering learning dynamics, we have focused on an adaptive payoff-maximizing player against a fixed unbending coplayer in various scenarios that directly complement the original study by Press and Dyson, where they assume an evolutionary adaptive player against a fixed extortionate ZD coplayer (8). In previous experiments with human subjects, it was observed that while some players refuse to comply with unfair demands to discourage extortionate behavior, some players who consistently refuse to be extorted may eventually give up on punishing extortionists (10, 21). This situation can arise when the extortionists are known to be preprogrammed computer agents (21) or when they are incentivized to win an advantage (10), making it challenging to discipline them effectively. To address this tug-of-war situation in the adaptive dynamics of behavior response, we introduce a relative time scale *ω* that governs the time evolution of the behavioral change of an unbending player as compared to their coplayer. Specifically, the coadaptive dynamics between a ZD player X ($p=[p_{1},p_{2},p_{1},p_{2}]$) and an unbending player Y from class A (starting from a prescribed $q=[1,q_{2},0,q_{4}]$) under the previous donation games can be described by the following system of differential equations:


(7) $$\left\{ \begin{array}{c} \frac{dp_{1}}{dt}=(1-\omega )\frac{\partial s_{X}(p,q)}{\partial p_{1}},\\ \frac{dp_{2}}{dt}=(1-\omega )\frac{\partial s_{X}(p,q)}{\partial p_{2}},\\ \frac{dq_{2}}{dt}=\omega \frac{\partial s_{Y}(p,q)}{\partial q_{2}},\\ \frac{dq_{4}}{dt}=\omega \frac{\partial s_{Y}(p,q)}{\partial q_{4}}. \end{array}\right.$$

As *ω* approaches 0, the dynamics revert back to the original scenario we studied (Fig. 5), where an unbending player is fixed in their behavior. Conversely, when *ω* approaches 1, the dynamics converge to the scenario studied by Press and Dyson, which features a fixed ZD player. For intermediate values of *ω*, an interesting arms race emerges between the two adaptive players, which is similar to the Red Queen dynamics. Namely, adapting quickly means being responsive to instant payoff improvement, but this may lead to unexpected shifts in the game where one player turns out to have more influence than the other in the long run. Our theoretical findings are supported by an analysis of the extended coadaptive dynamics between a ZD player and their unbending coplayer from class A or class D. Our results show that unbending players, even when their learning rates are high, can enforce fairness and cooperation in pairwise interactions as long as they retain some degree of unbending characteristics, as demonstrated in Fig. S22 in the Online Supplementary Material.

Taken together, these results suggest that unbending strategies cannot just outlearn self-interested extortionate ZD and force them to be fair and cooperative (that is, both parties eventually get equal payoffs out of mutual cooperation, Figs. S22a and b, S22d and e in the Online Supplementary Material), but also steer the evolution of TFT-like strategies out of any focal player using a much broader strategy space (represented by the unit square $[0,1{]}^{2}$ in Fig. 5) including but not necessarily limited to extortionate ZD strategies (namely, the edge BA in Fig. 5).

**Fig. 6.**
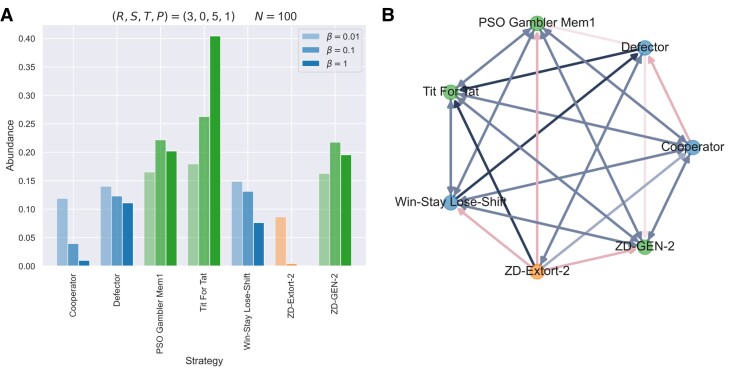
Evolutionary dynamics of unbending strategies in finite populations. Shown are a) the stationary abundance of IPD strategies (as indicated on the *x*-axis) under the limit of rare mutations and for different selection strengths *β* and b) the evolutionary pathways in pairwise competition dynamics (the direction of the arrows indicates dominance where the IPD strategy at the end is favored over that at the start; double arrows indicate neutral evolution). We consider a set of prescribed IPD strategies, including members belonging to unbending strategies from class A (the PSO Gambler) and from class D (generous ZD with χ′=2). TFT is an extreme boundary case of unbending strategies from class D (cf. point A in Fig. 3c). We use the Moran process for evolutionary updating and study the long-term mutation-selection equilibrium. Parameters: population size N=100, mutation rate μ→0, selection strength β=0.01, 0.1, 1, payoff values: R=3, S=0, T=5, P=1.

So far, we have focused on characterizing properties of unbending strategies and demonstrating their steering role in enforcing fairness and cooperation in pairwise interactions that only involve two parties. It is equally, if not less, worthy of studying the evolutionary dynamics of unbending strategies in stochastic population dynamics together with a set of other prescribed IPD strategies. As shown in Fig. 6, we demonstrate the evolutionary advantage of unbending strategies and their stability in stochastic dynamics of invasion and fixation under the limit of rare mutations (28). We see that unbending strategies, including class A (i.e. the PSO Gambler) and class D (i.e. generous ZD and TFT), are favored by natural selection; their abundance is greater than the population average, which holds from weak selection (β=0.01) to strong selection (β=1). Noticeably, the abundance of extortionate ZD almost vanishes under strong selection (see Fig. 6a). In pairwise competition dynamics (arising from the limit of rare mutations such that the system has at most two IPD strategies simultaneously present in the population), the PSO Gambler, generous ZD, and TFT all have an evolutionary advantage over extortionate ZD (see Fig. 6b). Namely, the fixation probability of an unbending strategy is greater than that of an extortionate ZD (see Online Supplementary Material for details).

Nevertheless, we note that the general picture depicted here could qualitatively change for extremely small population size *N* (see Online Supplementary Material, and Fig. S23). In fact, extortionate ZD can be favored over unbending strategies (UB), such as the PSO Gambler, if N=2. To see this, let us express the 2×2 average payoff matrix for their game interactions as follows:


(8) $$\begin{array}{cccc} & & \mathrm{UB} & \mathrm{ZD}\\ \mathrm{UB} & | & a_{11} & a_{12}\\ \mathrm{ZD} & | & a_{21} & a_{22} \end{array}$$

In a population of size *N*, UB is favored over ZD if and only if


(9) $$(N-2)a_{11}+Na_{12}>Na_{21}+(N-2)a_{22}.$$

This condition holds for any selection strength and for any mutation rate (29). As for the PSO Gambler vs an extortionate ZD (with χ′=2) using conventional payoff values, we have $a_{11}=R=3$, $a_{12}=1.5$, $a_{21}=2.0$ (ZD enforcing a linear relation, $a_{21}-P=\chi \prime (a_{12}-P)$), and $a_{22}=P=1$. For N=2, ZD completely dominates UB as $a_{21}>a_{12}$. Only for population size N>8/3 (i.e. N≥3) is it possible for natural selection to favor UB over ZD.

Moreover, the presence of noise can have an impact on the evolutionary performance of IPD strategies, as demonstrated by the susceptibility of TFT against noise (4). In the Online Supplementary Material, we quantify how the level of implementation errors ϵ impacts the ability to foster mutual cooperation among unbending strategies themselves (see Online Supplementary Material for further details, Fig. S24). Like WSLS, unbending strategies, such as the PSO Gambler, are robust against noise; their mutual cooperation level $v_{\text{CC}}$ is impacted only as 1−O(ϵ). Altogether, our results demonstrate that previously unforeseen unbending properties actually exist in some common IPD strategies and that they can be leveraged to foster fairness and cooperation not only in pairwise interactions but also in population dynamics settings.

## Discussions

It is thought that an evolutionary (adapting) player should be subdued to a fixed extortionate ZD player by fully cooperating as the best response (8). In contrast, recent experimental evidence suggests that human players often choose *not* to accede to extortion out of concern for fairness (10, 15). Inspired by this empirical finding, here we show that there exist general classes of *unbending* strategies such that the best response of any payoff-maximizing extortioner against a fixed unbending player is to be fair, thereby ensuring equal pay for both parties. From this perspective, the witting of unbending strategies has effectively turned the opponent’s choices of whether or not to adopt extortionate ZD strategy into an Ultimatum game (30): to demand unfair division via unilaterally setting a large χ value, or to guarantee fair share by letting χ′→1. In the former, the extortion effort is sabotaged by unbending, and both sides will be hurt, whereas in the latter, both sides will get an equal split of the payoffs. Our results demonstrate that unbending strategies can be used to rein in seemingly formidable extortionate ZD players, whose fair offer ultimately can be cultivated in their own interest.

In light of unbending strategies, there is no guarantee that extortioners will be able to subdue their opponents with certainty and get their own way as desired. Extortion cannot be successful unless their coplayers give up resistance in the first place. The extortion ZD exerts on the coplayer can backfire on themselves. For example, an extortionate ZD player will not be able to rein in TFT-like players (a limiting case belonging to class D of unbending strategies) who are fair-minded but willing to punish defection by responding with defection. They will end up in a tie both receiving *P* (Fig. 2). Even if an extortionate ZD player *X* does end up with dominance over the coplayer Y, namely, $s_{X}>s_{Y}>P$, a higher ratio of relative payoff surplus, $\chi \prime =(s_{X}-P)/(s_{Y}-P)$, does not necessarily translate to higher actual payoffs (Fig. 2a). Increasing χ appears to put ZD in a more advantageous position, but such unfair demand would be pushed back by unbending players such as generous ZD and TFT-like players and hence does not always yield higher actual payoffs (Fig. 4a). As recently demonstrated in experiments involving human players against fixed machine extortioners (15), human players respond to more extortionate ZD players with much lower cooperation levels, which are in essence passive punishment measures to counter ZD’s intended extortion.

Unbending behavior is related to the concept of fairness, which has been extensively studied in economics and game theory, particularly through the Ultimatum game (31–34). In such games, individuals may refuse to make concessions they perceive as unfair, even if it would be rational to do so. Third-party mechanisms such as punishment (35) and reputation effects (30) are often necessary to enforce cooperative behavior or fairness. However, in repeated games like the PD, individuals can employ a variety of strategies to balance their expectations of fair play and reinforce niceness, which can lead to reciprocal fairness and cooperation. The unbending strategies we discovered may be part of the repertoires used to counteract the ZD’s extortion and foster mutual cooperation and cultivate fairness, given the ubiquity of unbending properties in some common IPD strategies (Fig. 3).

Our work highlights the importance of payoff structure in determining the optimality of ZD strategies in IPD games (Fig. 1). In particular, if the condition T+S<2P holds, which means the total payoff, T+S, from alternating C and D pairs of two players is worse than that of their mutual defection, 2P, extortionate ZD players can be outperformed (Fig. 1b and d). This surprising finding is an important new insight that stems from the present study. Moreover, the sign of T+S−2P qualitatively determines the admissible strategy space of unbending players that can cause the backfire on extortioners (Fig. 3). Noticeably, only if T+S<2P is WSLS an unbending strategy, and in this case, WSLS dominates any extortionate ZD strategy (Fig. 4b).

The payoff condition T+S<2P implies a more adversarial nature in pairwise interactions than the conventional IPD games where T+S>2P typically holds (24). Intuitively, this means that the best response for a pair of individuals alternating between (C, D) and (D, C) is always to switch to mutual defection (D, D) (cf. Fig. 1a and b). Unbending strategies (those highlighted in the shaded area in Fig. 3b) can outcompete seemingly invincible extortionate ZD players who would have the greatest potential to dominate by using the minimal O=P and χ′>1 (Fig. 1). As aforementioned, since ZD’s strategy is parameterized explicitly by the underlying payoff matrix, variations in the payoff structure can have a previously unforeseen effect that will turn the tables on ZD (Fig. 4b): an extortionate ZD may become a victim of their own success in IPD games satisfying T+S<2P and more broadly, in social dilemmas of more adversarial nature as discussed in Ref. (24).

In the presence of errors and noises (36, 37), complex strategies informed by longer memory of past moves are likely at an advantage against simple memory-one strategies. Beyond pairwise interactions, higher order ones in multiperson games (38), such as the public goods game, as well as asymmetric interactions (39), are also of relevance in studying reciprocity in these generalized situations. Extensions incorporating these considerations are meaningful, but incur computational and theoretical challenges in search of robust optimal strategies. Nevertheless, the recent breakthrough in reinforcement (deep) learning of zero-sum games (40), like the Go (41), can lend some insight into the study of nonzero-sum games where learning agents, despite being self-serving, can mutually foster cooperation for the greater good under certain conditions (42). Thus, the classic framework of IPD still has the potential to be used as a primary testbed for synergistically combining artificial intelligence (AI) and game theory in future work (13, 43, 44), all with an eye towards helping us to enhance global cooperation in many challenging issues confronting our common humanity (45).

While the theoretical and modeling insights of unbending strategies are enlightening, we would like to briefly discuss the limitations of their practical success. First, when individuals encounter unknown coplayers with limited prior information, accurately detecting and assessing potential extortion during repeated interactions may prove challenging due to cognitive constraints. This presents an obstacle to effectively countering extortion using unbending strategies in real time. Second, even when unbending players are made fully aware of the extortion, they may give up their resistance efforts all too quickly, as it could be more beneficial for them to do so, according to recent empirical findings that show extortion prevails under incentive (10) or power (46, 47) asymmetries (see Ref. (48) for a mini review). Moving forward, we hope the present study will help stimulate future studies, both empirical and theoretical, to assess the efficacy of unbending strategies in more realistic scenarios, such as those involving unknown coplayers or short-term incentives that encourage self-interest.

In summary, we have found and characterized general classes of unbending strategies that are fair-minded and can outlearn extortioners in their head-to-head encounters. When an extortionate ZD player attempts to demand an unfair greater share from an unbending player who instead uses a fixed strategy, the unbending player is able to restrain the extortioner from profiting more. The intent to extort an unbending player has unprecedented consequences: extortioners would fare worse than if being fairer, and they can even be outperformed by, for example, WSLS, if the payoff matrix satisfies T+S<2P. Such previously unforeseen backfires caused by unbending players can steer reactive learning dynamics of extortionate ZD players from extortion to fairness. Our work offers novel insights into fostering fairness and suppressing extortion for a more equitable and just society.

## Model and methods

### Model and analytical approach

We use the same analytical approach invented by Press and Dyson (8) to calculate the expected payoffs of any two given players that are head-to-head in the IPD games. In this work, we focus on revealing strategies that are unbending to extortionate ZD players using explicit closed-form solutions (see details in the Online Supplementary Material). The ZD strategies are usually parameterized by three important parameters, the extortion factor χ, the normalization factor ***ϕ***, plus an additional baseline payoff O∈[P,R] which controls the level of generosity (8, 19). Tuning the parameter ***ϕ*** of extortionate ZD strategies with O=P and χ′>1 does not affect the linear payoff relation $s_{X}-P=\chi \prime (s_{Y}-P)$, but will impact the dependence of their own average payoffs on the extortion factor χ in a nontrivial way (see Fig. 2). Therefore, we restrict our search for unbending strategies that can neutralize the impact of this parameter ***ϕ***, that is, we find specific classes of strategies that are able to render the independence of their payoffs on ***ϕ***. Further, we narrow down the search of unbending strategies that can cause the “backfire” of extortion, namely, the expected payoffs of extortionate ZD strategies against a fixed unbending player are monotonically decreasing with χ. Ultimately, these considerations lead us to discover multiple general classes of unbending strategies, against which attempt to extort and dominate, if any, does not pay off at all for ZD players using O=P+ε≥P (including but not limited to extortionate ZD). In some cases, extortionate ZD strategies can even be outperformed by unbending coplayers, if the payoff matrix satisfies 2P<T+S (Fig. 3b). We also investigate how fixed unbending players can steer the learning dynamics of their adapting coplayers who use a much broader range of memory-one strategies beyond the class of extortionate ZD towards fairness and cooperation. We detail our comprehensive analysis in the Online Supplementary Material (see Figs. S1–S24 and Tables S1–S21).

## Supplementary Material

pgad176_Supplementary_Data

## Data Availability

Our Python Jupyter notebooks that can be used to reproduce results reported in this work are available at GitHub: https://github.com/fufeng/unbending.

## Previous presentation

These results were not previously presented.

## Preprints

A preprint of this article is published at arXiv:2201.04198.
