# Supplementary material for: Outlearning extortioners: unbending strategies can foster reciprocal fairness and cooperation
Source: PNAS Nexus. 2023 May 25;2(6):pgad176. doi: 10.1093/pnasnexus/pgad176 (PMC10244001; doi:10.1093/pnasnexus/pgad176)
Supplement: pgad176_Supplementary_Data [file pgad176_supplementary_data.pdf]

Supplementary Information for "Outlearning extortioners: unbending strategies can foster reciprocal fairness and cooperation"

Xingru Chen, Feng Fu

March 25, 2023

Contents

1 Introduction 3

2 The role played by ϕ and the maximization of payoffs 6

3 The unbending strategies 10

4 Class A of unbending strategies 12
- 4.1 Case I: $T + S > 2P$ . . . 13
  - 4.1.1 The conventional IPD game . . . 13
  - 4.1.2 The donation game . . . 15
  - 4.1.3 The general IPD game . . . 17
- 4.2 Case II: $T + S = 2P$ . . . 20
- 4.3 Case III: $T + S < 2P$ . . . 21

5 Class B of unbending strategies 22

6 Class C of unbending strategies 23
- 6.1 Case I: $T + S > 2P$ . . . 24
- 6.2 Case $T + S = 2P$ . . . 24
- 6.3 Case III: $T + S < 2P$ . . . 25

7 Class D of unbending strategies 26
- 7.1 Case I: $T + S > 2P$ . . . 27
- 7.2 Case II: $T + S = 2P$ . . . 28
- 7.3 Case III: $T + S < 2P$ . . . 29

8 The impact of unbending strategies on extortionate ZD strategies: dominance, average payoffs, and best response **30**

8.1 Comparing s_X and P . . . 31

8.2 Comparing s_X and s_Y . . . 32

8.3 Monotonicity of s_Y . . . 32

8.4 Maximum values of s_X and s_Y . . . 33

8.5 Figures . . . 34

9 The complete picture of extortionate ZD's superiority: the need for studying $T + S < 2P$ **38**

10 The robustness of extortion ability of general ZD strategies **41**

11 The role played by ϕ (continued) **42**

12 Steering learning dynamics from extortion to fairness and cooperation: reactive strategies **44**

13 Under influence of class A of unbending strategies (continued) **46**

13.1 Maximum value of s_X . . . 47

13.2 Monotonicity of s_X . . . 48

13.2.1 Partial derivative with respect to p_1 . . . 48

13.2.2 Partial derivative with respect to p_2 . . . 51

13.3 Conclusion and example . . . 54

14 Under influence of class D of unbending strategies (continued) **57**

14.1 Maximum value of s_X . . . 57

14.2 Monotonicity of s_X . . . 57

14.2.1 Partial derivative with respect to p_1 . . . 58

14.2.2 Partial derivative with respect to p_2 . . . 58

14.3 Conclusion and example . . . 58

15 Steering learning dynamics from extortion to fairness and cooperation: general ZD strategies **59**

15.1 Under influence of class A of unbending strategies (continued) . . 60

15.2 Under influence of class D of unbending strategies (continued) . . 60

15.2.1 Partial derivative with respect to O . . . 61

16 Extended search of unbending strategies against general ZD co-players **62**

17 Class A of unbending strategies (continued) **65**

17.1 Case II: $T + S = 2P$ 66

17.2 Case III: $T + S < 2P$ 67

18 Class D of unbending strategies (continued) **68**

19 Arms race of adaptive learning dynamics between unbending players and their co-players **70**

20 Beyond pairwise interactions: evolutionary stability of unbending strategies in population dynamics settings **72**

21 Noisy IPD games with implementation errors **74**

References **76**

1 Introduction

Years have passed since Press and Dyson discovered the class of zero-determinant (ZD) strategies, some of which seem to be able to dominate any evolutionary opponent in the Iterated Prisoner's Dilemma (IPD) game. Assume that there are two players X and Y. Label the four outcomes of a single round 1, 2, 3 and 4 for $\boldsymbol{xy} \in (\mathrm{CC}, \mathrm{CD}, \mathrm{DC}, \mathrm{DD})$. We can write X's strategy as $\boldsymbol{p} = (p_1, p_2, p_3, p_4)$, denoting the probabilities to cooperate under these outcomes. Analogously, Y's strategy is $\boldsymbol{q} = (q_1, q_2, q_3, q_4)$. When X applies a ZD strategy, its payoff s_X and the opponent's payoff s_Y satisfy the equation

$$\alpha s_X + \beta s_Y + \gamma = 0. \tag{1}$$

Further, consider the above linear relation between s_X and s_Y of the form:

$$s_X - O = \chi(s_Y - O), \tag{2}$$

where O is the baseline payoff for both players, χ is the extortion factor, and ϕ is the parameter that guarantees $0 \le p_i \le 1$ for $i \in \{1, 2, 3, 4\}$. Routine calculation gives the corresponding probabilities for player X:

$$\begin{cases} p_1 = 1 - (R - O)\phi(\chi - 1), \\ p_2 = 1 - \phi[(T - O)\chi + (O - S)], \\ p_3 = \phi[(O - S)\chi + (T - O)], \\ p_4 = (O - P)\phi(\chi - 1). \end{cases} \tag{3}$$

Here, the range of χ is

$$\chi \ge 1 \qquad \text{or} \qquad \chi \le \chi^{\text{upper}} = \begin{cases} -\frac{T-O}{O-S}, & T + S \ge 2O \\ -\frac{O-S}{T-O}. & T + S < 2O \end{cases} \tag{4}$$

Given that all the components of p are between 0 and 1, O has to be in the interval $[P, R]$. Choosing P will lead to the meanest ZD strategy (extortioner) while choosing R will generate the most generous one (complier). In our study, we let $O = P$ most of the time.

If X is an extortioner, we have

$$s_X - P = \chi(s_Y - P), \tag{5}$$

which yields the extortionate ZD strategy with probabilities

$$\begin{cases} p_1 = 1 - (R-P)\phi(\chi - 1), \\ p_2 = 1 - \phi[(T-P)\chi + (P-S)], \\ p_3 = \phi[(P-S)\chi + (T-P)], \\ p_4 = 0. \end{cases} \tag{6}$$

Regarding the two parameters in Equation 6, the extortion factor $\chi \geq 1$ by convention. Meanwhile, the legitimate range of ϕ is (a typo has been pointed out in Press and Dyson)

$$0 < \phi \leq \phi^{\text{upper}} = \begin{cases} \frac{1}{(T-P)\chi+(P-S)}, & T+S \geq 2P \\ \frac{1}{(P-S)\chi+(T-P)}. & T+S < 2P \end{cases} \tag{7}$$

Notice that it is also possible for player X to obtain a payoff greater than that of player Y when the extortion factor χ satisfies

$$\chi \leq \chi^{\text{upper}} = \begin{cases} -\frac{T-P}{P-S}, & T+S \geq 2P \\ -\frac{P-S}{T-P}. & T+S < 2P \end{cases} \tag{8}$$

In this case, the parameter ϕ needs to follow the inequality $\phi^{\text{lower}} \leq \phi < 0$, where ϕ^{lower} can be derived under the same restriction that p_1, p_2, p_3, and p_4 should be reasonable probabilistically. Moreover, since χ is negative, it is straightforward to see that $s_X < P < s_Y$ (Figure S2) or $s_Y < P < s_X$ (Figure S4).

The linear relation between the two payoffs s_X and s_Y convinces latecomers that (i) the extortioner's payoff s_X is always no less than the opponent's payoff s_Y and (ii) s_X is an increasing function (at least a non-decreasing function) with respect to the extortion factor χ (on the two branches of χ).

The claim above holds at large. When we work on the conventional values of an IPD game, that is, $(R, S, T, P) = (3, 0, 5, 1)$, it is true for common strategies such as ALLC, whose $\boldsymbol{q} = (1, 1, 1, 1)$, Random, whose $\boldsymbol{q} = (0.5, 0.5, 0.5, 0.5)$, and Win-Stay Lose-Shift (WSLS), whose $\boldsymbol{q} = (1, 0, 0, 1)$ (see Figure S1 (a)). Also, if we assume that $T + S > 2P$ and the opponent Y fixes its cooperation rate by taking an unconditional memory-one strategy with $\boldsymbol{q} = (q, q, q, q)$, routine calculation shows that the derivative of ZD's payoff will be

$$\frac{ds_X}{d\chi} = \frac{q(T-S)[(T-R)q+(P-S)(1-q)][(R-P)q+(T+S-2P)(1-q)]}{f^2(\chi)}, \tag{9}$$

where $f(\chi) = [(R-S)q + (T-P)(1-q)]\chi + [(T-R)q + (P-S)(1-q)]$ is a linear function of χ. Apparently, the derivative is positive as long as q is nonzero and hence s_X is an increasing function of χ on both branches.

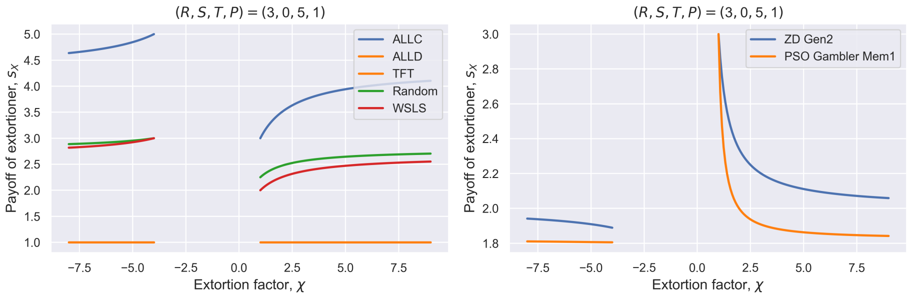

(a) (b)

Figure S1: Extortioner's payoff against different strategies. We consider the conventional IPD game.

However, the above results may not tell the complete story. Figure S1(b) indicates that even for the conventional IPD game, an extortioner's payoff does not necessarily increase with χ. For another example, if $(R, S, T, P) = (1, -3, 2, 0)$, Figure S2 shows that the well-known strategy WSLS, whose $\boldsymbol{q} = (1, 0, 0, 1)$, can not only cause s_X to decrease with χ but also bludgeon the extortioner into accepting a payoff less than the punishment P.

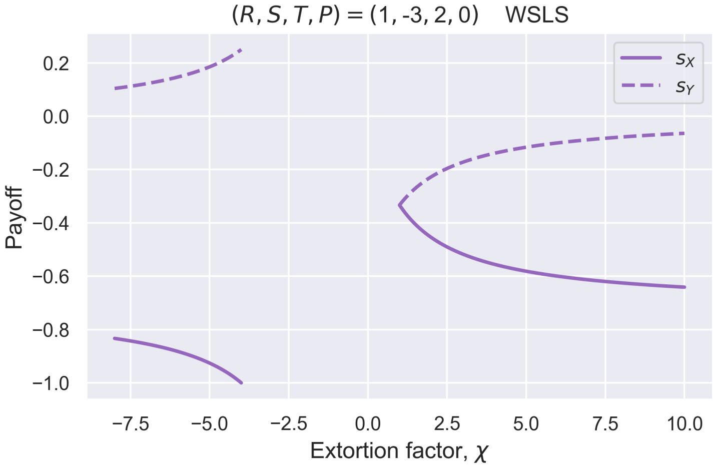

Figure S2: Payoffs of the two players X and Y. The extortionate ZD strategy is applied by X and the WSLS strategy by Y. We work on a IPD game with payments satisfying $T + S < 2P$.

Therefore, given the four payoffs R, S, T and P satisfying $T > R > P > S$ and $T + S < 2R$, there exist strategies playing against which an extortioner's payoff may not always increase as χ increases on either branch. The monotonicity of s_X is dependent on the opponent's strategy. In particular, we have found a set of memory-one strategies that cannot be extorted by any ZD strategy with extortion factor χ greater than 1. We refer to them as unbending strategies.

Definition 1.1. A memory-one strategy is unbending if for any extortionate ZD co-player with extortion factor $\chi > 1$, the co-player's payoff is independent of ϕ and monotonically decreases with respect to χ.

Contrary to the current consensus on ZD strategies, if $\boldsymbol{q}$ is an unbending strategy, it will force the payoff of the extortionate ZD co-player to decrease as χ increases from 1 to $+\infty$ if $T + S \geq 2P$. It may even impose a payoff less than P for the co-player should $T + S < 2P$.

2 The role played by ϕ and the maximization of payoffs

Recall that another parameter ϕ appears in the expression of $\boldsymbol{p}$ (see Equation 6). If the opponent Y tries a common strategy such as ALLC, the payoff of X will be $(T - S)(R - P)\chi/[(R - S)\chi + T - R] + P$, which does not involve ϕ. However, when X plays against a more general opponent, its payoff is oftentimes dependent on ϕ. Take the conventional IPD game as an example (see Figure S3). It is apparent that s_X can either increase or decrease with ϕ. And even if we have fixed $\boldsymbol{q}$, it is possible to alter the monotonicity of s_X by choosing different ϕ.

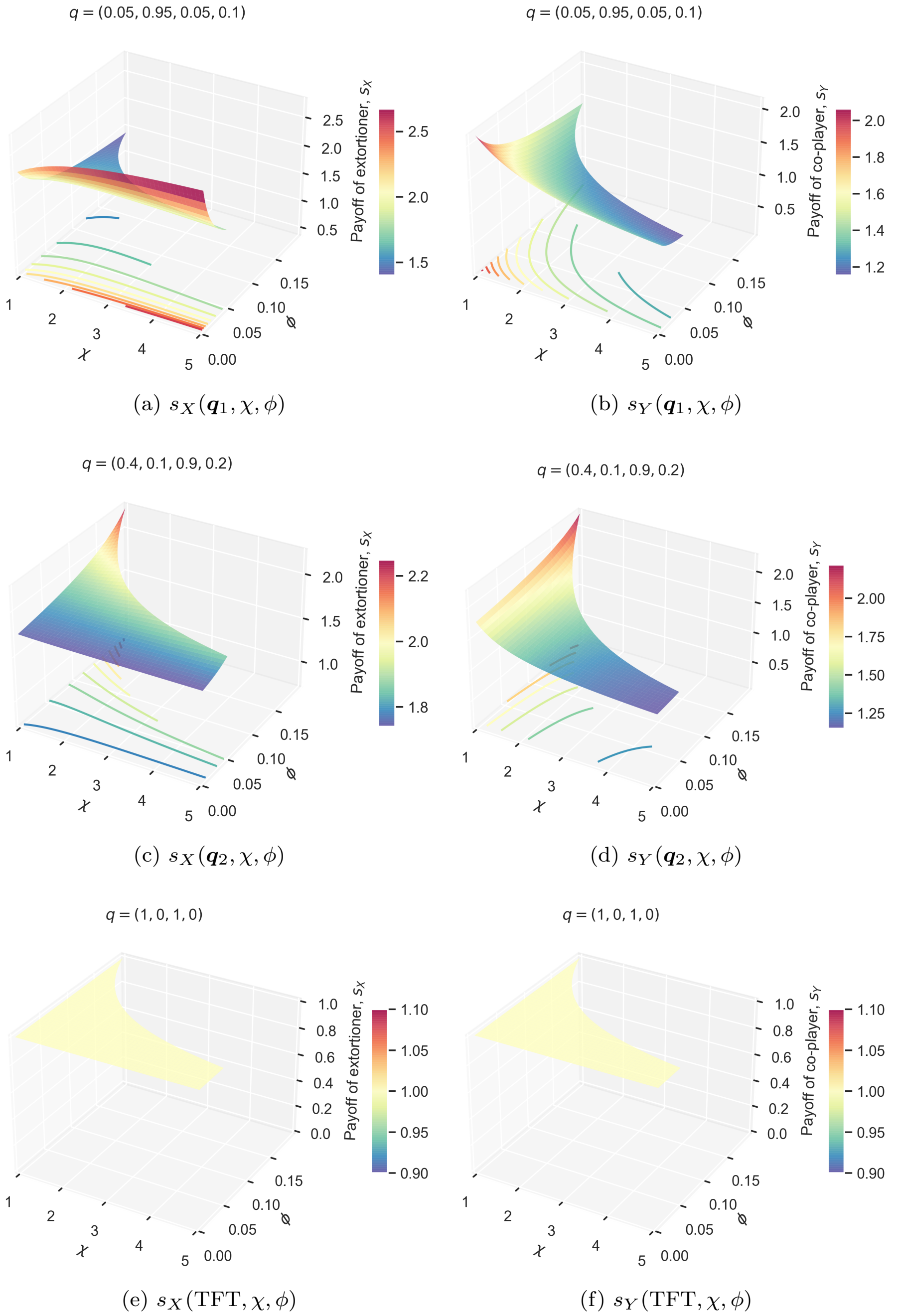

(a) $s_X(\boldsymbol{q}_1, \chi, \phi)$ (b) $s_Y(\boldsymbol{q}_1, \chi, \phi)$

(c) $s_X(\boldsymbol{q}_2, \chi, \phi)$ (d) $s_Y(\boldsymbol{q}_2, \chi, \phi)$

(e) $s_X(\mathrm{TFT}, \chi, \phi)$ (f) $s_Y(\mathrm{TFT}, \chi, \phi)$

Figure S3: Surfaces of s_X (s_Y). We work on the conventional IPD game where strategy $\boldsymbol{q}$ is applied by player Y. A point (x, y, z) in the 3-dimensional space represents the corresponding tuple consisting of the extortion factor χ, the parameter ϕ, and extortioner's payoff s_X (opponent's payoff s_Y). Its color indicates the value of s_X (s_Y). The contour curves on the xy-plane are also given.

Therefore, unbending strategies should be a subset of strategies playing against which the extortioner's payoff is independent of ϕ. To find the latter, we consider a necessary condition

$$s_X(\boldsymbol{q}, \chi, \phi^{\text{upper}}) = s_X(\boldsymbol{q}, \chi, \frac{\phi^{\text{upper}}}{2}). \tag{10}$$

The equation has six solutions, which further yield six expressions of s_X (see Table S1). These s_X's are all free of ϕ. Therefore, the condition is both necessary and sufficient. Examples are given in Figure S4.

Solution	s_X
$q_1 = q_2 = 1$	$\frac{(T-S)(R-P)\chi}{(R-S)\chi+(T-R)} + P$
$q_4 = 0$	P
$q_1 = 1$ and $q_3 = 0$	$\frac{(T-S)(R-P)\{[(P-S)q_2+T+S-2P]\chi+(T-P)q_2-(T+S-2P)\}q_4\chi}{f_A(\chi)} + P$
$q_2 = q_3 = 0$	$\frac{(T-S)(T+S-2P)q_4\chi}{f_B(\chi)} + P$
$q_1 = q_2 = q_3$	$\frac{(T-S)[-(T+S-R-P)q_1+T+S-2P]q_4\chi}{f_C(\chi)} + P$
$q_4 = h_D$	$\frac{(T-S)[-(T+S-2P)q_1+(R-P)(q_2+q_3)+T+S-R-P]\chi}{f_D(\chi)} + P$

Table S1: The solutions of $\boldsymbol{q}$ for s_X being independent of ϕ and the corresponding expressions of s_X. Here, h_D is a multivariate linear function of q_1, q_2 and q_3. Additionally, f_A is a quadratic function of χ, and f_B, f_C, and f_D are linear functions of χ.

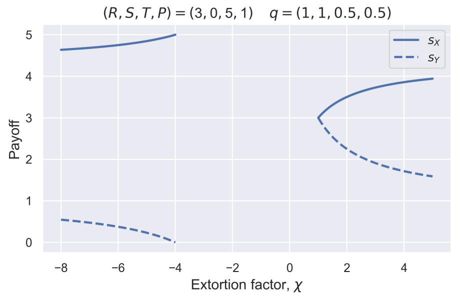

(a) $q_1 = q_2 = 1$

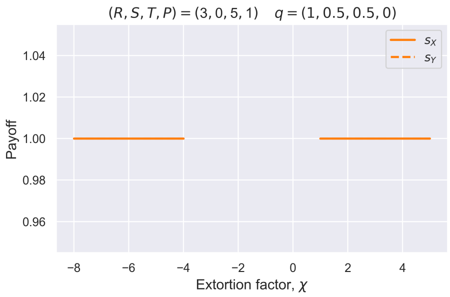

(b) $q_4 = 0$

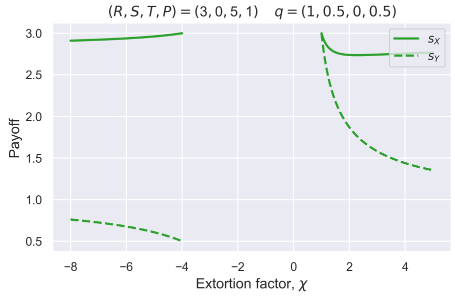

(c) $q_1 = 1$ and $q_3 = 0$

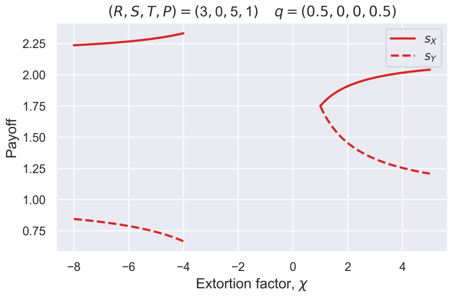

(d) $q_2 = q_3 = 0$

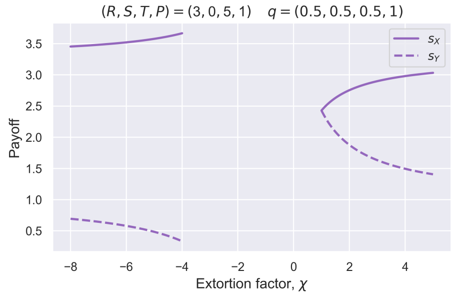

(e) $q_1 = q_2 = q_3$

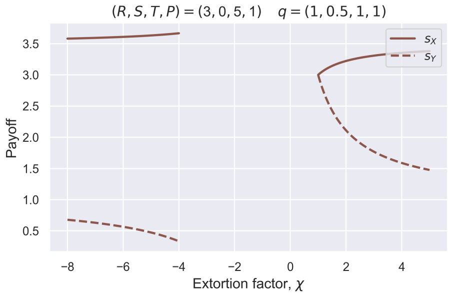

(f) $q_4 = h_D$

Figure S4: Payoffs of the two players X and Y. We work on the conventional IPD game where strategy $\boldsymbol{q}$ is applied by Y. For every panel, $\boldsymbol{q}$ satisfies one of the six solutions in Table S1.

For a few side notes, we emphasize that if the opponent's strategy $\boldsymbol{q}$ fulfills any condition other than the third one in Table S1, the extortioner's payoff will have the same monotonicity for $\chi < \chi^{\text{upper}}$ and $\chi > 1$. The proof is trivial. Whenever s_X is a linear rational function of χ, its curve is a hyperbola and is monotonically increasing or decreasing on both the left and the right branches. Besides, when $\chi = 1$, the IPD game is likely to enter an absorbing state. Given Equation 6, we have $p_1 = 1$ when $\chi = 1$. If the opponent satisfies $q_1 = 1$, there will always be mutual cooperation and hence $s_X = s_Y = R$. On the other hand, we will always have $s_X = s_Y = P$ when $q_4 = 0$.

Therefore, without loss of generality, we assume that $\chi > 1$ and $0 < q_4 \leq 1$ in the discussion that follows.

3 The unbending strategies

Given the above results, we are now ready to figure out the set of unbending strategies.

It is trivial to show that if $q_1 = q_2 = 1$ and $s_X = (T-S)(R-P)\chi/[(R-S)\chi+(T-R)]+P$, the derivative $ds_X/d\chi = (T-R)(T-S)(R-P)/[(R-S)\chi+(T-R)]^2$ will always be positive. Therefore, we only need to work on the last four solutions and explore the 4-dimensional space generated by $\boldsymbol{q} = (q_1, q_2, q_3, q_4)$ where $0 \leq q_i \leq 1$ for $i \in \{1, 2, 3\}$ and $0 < q_4 \leq 1$. We aim to obtain the set of $\boldsymbol{q}$'s against which player X has a monotonically decreasing payoff s_X with respect to χ.

Notice that $s_X - P$ can be rewritten as a quadratic or linear rational function of χ (recall Table S1) under any of the four solutions:

$$s_X - P = \frac{\chi(a_1\chi + a_0)}{d_2\chi^2 + d_1\chi + d_0}, \tag{11}$$

which may sometimes degenerate to

$$s_X - P = \frac{a_0\chi}{d_1\chi + d_0}. \tag{12}$$

The derivative of s_X is henceforth

$$s'_X = \frac{e_2\chi^2 + e_1\chi + e_0}{(d_2\chi^2 + d_1\chi + d_0)^2}, \tag{13}$$

or

$$s'_X = \frac{e_0}{(d_1\chi + d_0)^2}, \tag{14}$$

where

$$\begin{cases} e_2 = a_1 d_1 - a_0 d_2, \\ e_1 = 2a_1 d_0, \\ e_0 = a_0 d_0. \end{cases} \tag{15}$$

As a result, we only need to take into account the sign of $g(\chi) = e_2\chi^2 + e_1\chi + e_0$ to decide the monotonicity of s_X. If s_X is a decreasing function of χ, $g(\chi)$ has to satisfy one of the following arguments:

(i) $e_2 = e_1 = 0$ and $e_0 < 0$.

(ii) $e_2 = 0$, $e_1 < 0$ and $g(1) = e_1 + e_0 \leq 0$.

(iii) $e_2 < 0$, $g(1) = e_2 + e_1 + e_0 \leq 0$, and $g(-\frac{e_1}{2e_2}) < 0$ if $-\frac{e_1}{2e_2} > 1$ (the highest point of the parabola opening downwards is below the x-axis).

The above criterion yields the (explicit) expressions of unbending strategies (see Table S2).

Class \ Case	$T+S>2P$	$T+S=2P$	$T+S<2P$
A<br>$q_1=1, q_3=0$	$\begin{cases} q_a < q_2 < 1 \\ 0 < q_4 \leq h_A \end{cases}$	$\begin{cases} 0 < q_2 < 1 \\ 0 < q_4 \leq h_A(*) \end{cases}$	$\begin{cases} 0 \leq q_2 < 1 \\ 0 < q_4 \leq h_A \end{cases}$
B<br>$q_2=q_3=0$	$\emptyset$	$\emptyset$	$\begin{cases} 0 \leq q_1 \leq 1 \\ 0 < q_4 \leq 1 \end{cases}$
C<br>$q_1=q_2=q_3$	$\begin{cases} q_C < q_1 < 1 \\ 0 < q_4 < h_C \end{cases}$	$\begin{cases} q_C < q_1 < 1 \\ 0 < q_4 < h_C \end{cases}$	$\begin{cases} q_C < q_1 < 1 \\ 0 < q_4 < h_C \end{cases}$<br>or<br>$\begin{cases} 0 \leq q_1 < q_c \\ 0 < q_4 \leq 1 \end{cases}$
D<br>$q_4=h_D$	$\begin{cases} 0 \leq q_1, q_2, q_3 \leq 1 \\ k(q_1, q_2, q_3) < 0 < K(q_1, q_2, q_3) \end{cases}$		

Table S2: Four classes of unbending strategies.

The boundary functions in Table S2 are given in Table S3 and the boundary values are in Table S4. The $(*)$ after the piecewise function h_A indicates that the equality can only hold if the value of the function equals 1 and is not evaluated at the endpoint.

Class	Boundary Function	
A	$T+S>2P$	$h_A(q_2)=\begin{cases}1, & e_{21}<-e_{20}\\ -\frac{e_{20}}{e_{21}}. & \text{otherwise}\end{cases}$
	$T+S=2P$	$h_A(q_2)=\begin{cases}1, & q_2<\frac{3(2R-T-S)}{4R-T-3S}\\ \frac{(2R-T-S)(1-q_2)}{2[(R-S)q_2-(2R-T-S)]}. & \text{otherwise}\end{cases}$
	$T+S<2P$	$h_A(q_2)=\begin{cases}1, & q_2<\frac{(R-P)+(2R-T-S)}{2R-P-S}\\ \frac{(R-P)(1-q_2)}{(R-S)q_2-(2R-T-S)}. & \text{otherwise}\end{cases}$
C	$h_C(q_1)=\frac{[(R-S)q_1-(P-S)](1-q_1)}{(T-S)-(R-S)q_1}$	
D	$h_D=\frac{-(T+S-2P)q_1+(R-P)(q_2+q_3)+T+S-R-P}{2R-T-S}$ $\begin{cases}k(q_1,q_2,q_3)=(T+S-2P)(1-q_1)-(R-P)(1-q_2)+(R-P)q_3,\\ K(q_1,q_2,q_3)=(T-S)(1-q_1)-(R-S)(1-q_2)+(T-R)q_3.\end{cases}$	

Table S3: Boundary functions for unbending strategies. The expressions of e_{21} and e_{20} are given in the next section.

Class	A	C
Value	$q_a=\frac{(T+S-2P)(P-S)}{(T-P)(T-S)-(P-S)^2}$	$\begin{cases}q_c=\frac{2P-T-S}{R+P-T-S}\\ q_C=\frac{P-S}{R-S}\end{cases}$

Table S4: Boundary values for unbending strategies.

We now discuss the four different classes in detail.

4 Class A of unbending strategies

The first class is under the restriction of $q_1 = 1$ and $q_3 = 0$ where the extortioner's payoff s_X is a quadratic rational function of χ as shown in the previous section. We have

$$s_X=\frac{(T-S)(R-P)\{[(P-S)q_2+T+S-2P]\chi+(T-P)q_2-(T+S-2P)\}q_4\chi}{f_A(\chi)}+P. \tag{16}$$

The denominator $f_A(\chi)=d_{A2}\chi^2+d_{A1}\chi+d_{A0}$ is a quadratic function of χ with

$$\begin{cases}d_{A2}=[(T-R)(P-S)q_2+(T-S)(R-P)]q_4+(T-P)(R-P)(1-q_2),\\ d_{A1}=[T(T-P)-S(P-S)-R(T+S-2P)]q_2q_4-(T+S-2P)(R-P)(1-q_2),\\ d_{A0}=[(T-P)(R-S)q_2-(T-S)(R-P)]q_4-(R-P)(P-S)(1-q_2).\end{cases} \tag{17}$$

Notice that

$$\begin{cases} d_{A2} > 0, \\ f_A(1) = q_2 q_4 (T-S)^2 \geq 0, \\ 2d_{A2} + d_{A1} = (T-S)\{[(T-S)q_2 + (R-P)(2-q_2)]q_4 + (R-P)(1-q_2)\} > 0. \end{cases} \tag{18}$$

That is, $f_A(\chi)$ is a quadratic function which opens upwards and whose line of symmetry $\chi = -d_{A1}/2d_{A2}$ lies to the left of $\chi = 1$. Altogether we get $f_A(\chi) > 0$ for $\chi > 1$.

If $q_2 = 0$, we have

$$\begin{aligned} s_X &= \frac{(T-S)(T+S-2P)q_4\chi}{[(T-S)q_4 + T - P]\chi + (T-S)q_4 + P - S} + P, \\ \frac{ds_X}{d\chi} &= \frac{(T-S)(T+S-2P)[(T-S)q_4 + P - S]q_4}{\{[(T-S)q_4 + T - P]\chi + (T-S)q_4 + P - S\}^2}. \end{aligned} \tag{19}$$

And if $T + S = 2P$, we obtain

$$s_X = \frac{(T-S)(2R-T-S)q_2q_4\chi}{d_1\chi + d_0} + \frac{T+S}{2}, \qquad \frac{ds_X}{d\chi} = \frac{(T-S)(2R-T-S)q_2q_4d_0}{(d_1\chi+d_0)^2}, \tag{20}$$

where

$$\begin{cases} d_{A1} = 2[(T-R)q_2 + (2R-T-S)]q_4 + (2R-T-S)(1-q_2), \\ d_{A0} = 2[(R-S)q_2 - (2R-T-S)]q_4 - (2R-T-S)(1-q_2). \end{cases} \tag{21}$$

The set of unbending strategies depends on the relation between $T+S$ and $2P$. We present the details of our analysis in three different cases: $T+S > 2P$, $T+S = 2P$, and $T+S < 2P$.

4.1 Case I: $T + S > 2P$

Before considering a general IPD game satisfying $T + S > 2P$, we first work on two special cases: the conventional IPD game with payments $(R, S, T, P) = (3, 0, 5, 1)$ and the donation game with benefit b and cost c.

4.1.1 The conventional IPD game

Payoff Matrix	Class A	s_X	$\frac{ds_X}{d\chi}$
$\begin{bmatrix} 3 & 0 \\ 5 & 1 \end{bmatrix}$	$\begin{cases} \frac{3}{19} < q_2 < 1 \\ 0 < q_4 \leq \frac{2(1-q_2)(19q_2-3)}{3q_2^2-q_2+30} \end{cases}$	$\frac{10[(q_2+3)\chi+4q_2-3]q_4\chi}{f_A(\chi)} + 1$	$\frac{10q_4g_A(\chi)}{f_A^2(\chi)}$

Table S5: Summary of the conventional IPD game if the extortioner's co-player applies an unbending strategy from Class A.

Here, $f_A(\chi)$ is defined as before and $g_A(\chi) = e_2\chi^2 + e_1\chi + e_0$. We have

$$\begin{cases} d_{A2} = 2(q_2+5)q_4 + 8(1-q_2), \\ d_{A1} = 11q_2q_4 - 6(1-q_2), \\ d_{A0} = 2(6q_2-5)q_4 - 2(1-q_2), \end{cases} \tag{22}$$

and

$$\begin{cases} e_2 = (3q_2^2 - q_2 + 30)q_4 - 2(1-q_2)(19q_2 - 3), \\ e_1 = 4(q_2+3)[(6q_2-5)q_4 - (1-q_2)], \\ e_0 = 2(4q_2-3)[(6q_2-5)q_4 - (1-q_2)]. \end{cases} \tag{23}$$

In particular, if $q_2 = 0$, we obtain

$$s_X = \frac{15q_4\chi}{(5q_4+4)\chi + 5q_4 + 1} + 1, \qquad \frac{ds_X}{d\chi} = \frac{15q_4(5q_4+1)}{[(5q_4+4)\chi + 5q_4 + 1]^2}. \tag{24}$$

The visualization of Class A, which corresponds to the second column in Table S5, is given in Figure S5.

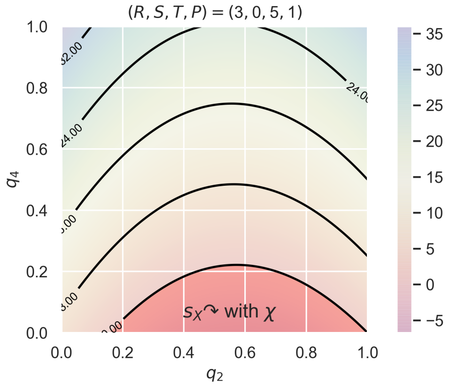

Figure S5: Contour curves of e_2 for the conventional IPD game. The red region is where $e_2 \leq 0$.

No more than fundamental calculus is needed to show that $e_2 \leq 0$ is the necessary and sufficient condition for the extortioner's payoff to be monotonically decreasing with χ (that is, $ds_X/d\chi < 0$). Therefore, we will not elaborate on the proof. The explicit expression of $e_2 \leq 0$ under the restriction that $0 < q_2, q_4 \leq 1$ is given in the second column of Table S5.

4.1.2 The donation game

Payoff Matrix	Class A
$\begin{bmatrix} b-c & -c \\ b & 0 \end{bmatrix}$	$\begin{cases} q_a < q_2 < 1 \\ 0 < q_4 \leq h_A(q_2) \end{cases}$
s_X	$\frac{ds_X}{d\chi}$
$\frac{(b+c)(b-c)[(cq_2+b-c)\chi+bq_2-(b-c)]q_4\chi}{f_A(\chi)}$	$\frac{(b+c)(b-c)q_4 g_A(\chi)}{f_A^2(\chi)}$

Table S6: Summary of the donation game if the extortioner's co-player applies an unbending strategy from Class A. The inequalities $b, c > 0$ and $b > c$ always hold.

The definitions of $f_A(\chi)$ and $g_A(\chi)$ are the same as before. Now we have

$$\begin{cases} d_{A2} = [c^2q_2 + b^2 - c^2]q_4 + b(b-c)(1-q_2), \\ d_{A1} = 2bcq_2q_4 - (b-c)^2(1-q_2), \\ d_{A0} = [b^2q_2 - b^2 + c^2]q_4 - c(b-c)(1-q_2), \end{cases} \tag{25}$$

and

$$e_i = e_{i1}q_4 + e_{i0}, \qquad i \in \{0,1,2\}, \tag{26}$$

where

$$\begin{cases} e_{21} = w_2q_2^2 + w_1q_2 + w_0, \\ e_{20} = (b-c)(1-q_2)(uq_2+v), \\ e_{11} = 2(cq_2+b-c)(b^2q_2-b^2+c^2), \\ e_{10} = -2c(b-c)(1-q_2)(cq_2+b-c), \\ e_{01} = (bq_2-b+c)(b^2q_2-b^2+c^2), \\ e_{00} = -c(b-c)(1-q_2)[bq_2-b+c], \end{cases} \tag{27}$$

and

$$\begin{cases} w_2 = bc^2, \\ w_1 = -(b-c)(b^2-bc-c^2), \\ w_0 = (b+c)(b-c)^2, \\ u = -b^2-bc+c^2, \\ v = c(b-c). \end{cases} \tag{28}$$

The expression of the boundary value q_a is

$$q_a = -\frac{v}{u} = \frac{c(b-c)}{b^2+bc-c^2}, \tag{29}$$

and that of the boundary function $h_A(q_2)$ is

$$h_A(q_2) = -\frac{e_{20}}{e_{21}} = \frac{(b-c)(1-q_2)[(b^2+bc-c^2)q_2 - c(b-c)]}{bc^2q_2^2 - (b-c)(b^2-bc-c^2)q_2 + (b+c)(b-c)^2}. \tag{30}$$

Notice that $e_{21} \geq w_1 q_2 + w_0 > w_0(1 - q_2) \geq 0$. In other words, e_{21} is positive all the time.

In particular, if $q_2 = 0$, we have

$$s_X = \frac{(b+c)(b-c)q_4\chi}{[(b+c)q_4 + b]\chi + (b+c)q_4 + c}. \tag{31}$$

The visualization of Class A, which corresponds to the second column in Table S6, is given in Figure S6.

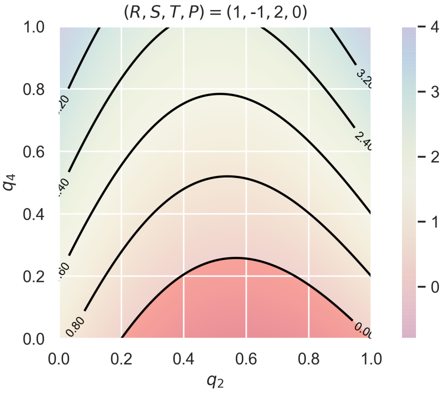

(a) $r = 2$

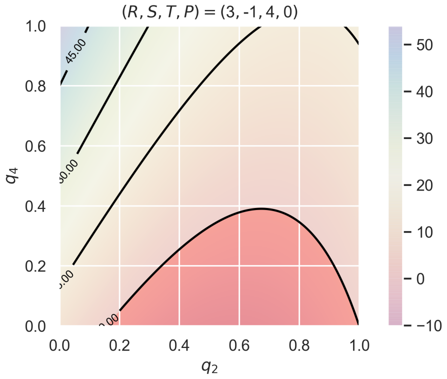

(b) $r = 4$

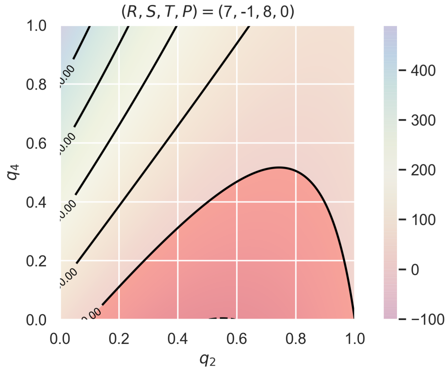

(c) $r = 8$

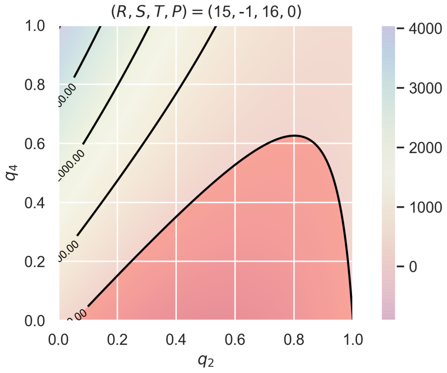

(d) $r = 16$

Figure S6: Contour curves of e_2 for the donation game with different benefit-to-cost ratios $r = b/c$. The red regions are where $e_2 \leq 0$. As the ratio increases, the area of the region increases accordingly.

As before, the set of inequalities regarding q_2 and q_4 is the explicit expression of $e_2 \leq 0$. We now show that $e_2 \leq 0$ is the necessary and sufficient condition for the extortioner's payoff to be monotonically decreasing with χ. Notice that the monotonicity is decided by the sign of $g_A(\chi)$, a quadratic function of χ. Therefore, $e_2 \leq 0$ is a necessary condition for s_X to decrease with respect to χ. It suffices to show that $e_2 \leq 0$ is also a sufficient condition.

If $e_2 = 0$, we have $q_4 = h_A(q_2)$ and $g_A(\chi)$ being a linear function of χ. Given that the slope e_1 satisfies

$$e_1 = -\frac{2b(b+c)^2(b-c)^2 q_2(1-q_2)^2(cq_2+b-c)}{e_{21}} < 0, \tag{32}$$

and the value of the function at $\chi = 1$ satisfies

$$g_A(1) = e_1 + e_0 = -\frac{b(b+c)^2(b-c)^2 q_2(1-q_2)^2[(b+2c)q_2+b-c]}{e_{21}} < 0, \tag{33}$$

we find that $g_A(\chi) < 0$ for $\chi > 1$.

Otherwise, if $e_2 < 0$, we immediately have $0 < q_4 < h_A(q_2)$ and $-\frac{u}{v} < q_2 < 1$. Further calculation yields that

$$g_A(1) = e_2 + e_1 + e_0 = (b+c)^2 q_2[(bq_2 - b + c)q_4 - (b-c)(1-q_2)], \tag{34}$$

of which the last factor on the right-hand side takes its maximum at $q_4 = 0$ or $q_4 = h_A(q_2)$. It is straightforward to show that

$$\max\{-(b-c)(1-q_2), -\frac{b(b-c)^2(1-q_2)^2[(b+2c)q_2+b-c]}{e_{21}}\} < 0. \tag{35}$$

Hence, $g_A(1) < 0$.

Moreover, we consider the sign of

$$2e_2 + e_1 = 2b(b+c)q_2[cq_2q_4 - (b-c)(1-q_2)]. \tag{36}$$

The last factor on the right-hand side takes its maximum at $q_4 = h_A(q_2)$ with value

$$-\frac{(b+c)(b-c)^2(1-q_2)^2(cq_2+b-c)}{e_{21}} < 0. \tag{37}$$

Therefore, $2e_2 + e_1 < 0$ and thus $-e_1/2e_2 < 1$.

Given the above results, we conclude that $g_A(\chi) < 0$ for $\chi > 1$.

4.1.3 The general IPD game

Payoff Matrix	Class A
$\begin{bmatrix} R & S \\ T & P \end{bmatrix}$	$\begin{cases} q_a < q_2 < 1 \\ 0 < q_4 \le h_A(q_2) \end{cases}$
s_X	s'_X
$\frac{(T-S)(R-P)\{[(P-S)q_2+T+S-2P]\chi+(T-P)q_2-(T+S-2P)\}q_4\chi}{f_A(\chi)}$	$\frac{(T-S)(R-P)q_4 g_A(\chi)}{f_A^2(\chi)}$

Table S7: Summary of the general IPD game with $T + S > 2P$.

Likewise, $g_A(\chi) = e_2\chi^2 + e_1\chi + e_0$, where

$$e_i = e_{i1}q_4 + e_{i0}. \qquad i \in \{0, 1, 2\} \tag{38}$$

More specifically, we have

$$\begin{cases} e_{21} = w_2 q_2^2 + w_1 q_2 + w_0, \\ e_{20} = (R-P)(1-q_2)(uq_2 + v), \\ e_{11} = 2[(P-S)q_2 + (T+S-2P)][(T-P)(R-S)q_2 - (T-S)(R-P)], \\ e_{10} = -2(R-P)(P-S)(1-q_2)[(P-S)q_2 + (T+S-2P)], \\ e_{01} = [(T-P)q_2 - (T+S-2P)][(T-P)(R-S)q_2 - (T-S)(R-P)], \\ e_{00} = -(R-P)(P-S)(1-q_2)[(T-P)q_2 - (T+S-2P)], \end{cases} \tag{39}$$

and

$$\begin{cases} w_2 = (R-S)(P-S)^2, \\ w_1 = (T-P)^2(T+P-2R) - (P-S)^3, \\ w_0 = (T-S)(R-P)(T+S-2P), \\ u = -[(T-P)(T-S) - (P-S)^2], \\ v = (P-S)(T+S-2P). \end{cases} \tag{40}$$

The expression of the boundary value q_a is

$$q_a = -\frac{v}{u} = \frac{(T+S-2P)(P-S)}{(T-P)(T-S)-(P-S)^2}, \tag{41}$$

and that of the boundary function $h_A(q_2)$ is

$$h_A(q_2) = \begin{cases} 1, & e_{21} \le -e_{20} \\ h(q_4) = -\frac{e_{20}}{e_{21}}. & \text{otherwise} \end{cases} \tag{42}$$

The visualization of Class A, which corresponds to the second column in Table S7 (the explicit expression of $e_2 \le 0$), is given in Figure S7.

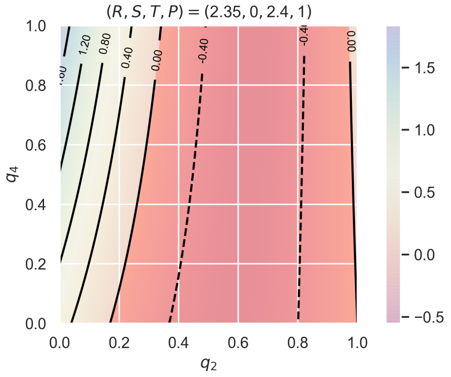

Figure S7: Contour curves of e_2 for the general IPD game with $T + S > 2P$. Here we have $(R, S, T, P) = (2.35, 0, 2.4, 1)$. The red region is where $e_2 \le 0$.

Notice that $e_{20} > 0$ implies $e_{21} > 0$. That is, if $0 < q_2 < q_a$, $e_{21} > 0$. The contrapositive statement also holds ($e_{21} \leq 0$ implies $e_{20} \leq 0$). The proof is trivial. Consider e_{21} as a bivariate function of R and q_2 (linear with respect to R). Letting $R = P$ and $R = T$, we have

$$\begin{cases} e_{21}(P, q_2) = q_2[(P-S)^3 q_2 + (T-P)^3 - (P-S)^3] > 0, \\ e_{21}(T, q_2) = (T-S)(1-q_2)[-(P-S)q_2 + (T-P)(T+S-2P)]. \end{cases} \tag{43}$$

The last factor of $e_{21}(T, q_2)$ takes its minimum $-(T+S-2P)^2(T-S)^2/u$ at $q_2 = q_a$, which is positive.

We now show that $e_2 \leq 0$ is the necessary and sufficient condition for the extortioner's payoff to be monotonically decreasing with respect to χ. As before, $g_A(\chi)$ is a quadratic function of χ. Thus, $e_2 \leq 0$ is a necessary condition for s_X to decrease. It suffices to show that $e_2 \leq 0$ is also a sufficient condition.

If $e_2 = 0$, we have $q_4 = h_A(q_2)$ and $g_A(\chi)$ being a linear function of χ. Given that the slope e_1 satisfies

$$e_1 = -\frac{2(T-S)^2(R-P)(R-S)(T+S-2P)q_2(1-q_2)^2[(P-S)q_2+T+S-2P]}{e_{21}} < 0, \tag{44}$$

and the value of the function at $\chi = 1$ satisfies

$$\begin{aligned} g_A(1) &= e_1 + e_0 \\ &= -\frac{(T-S)^2(R-P)(R-S)(T+S-2P)q_2(1-q_2)^2[(T+P-2S)q_2+T+S-2P]}{e_{21}} \\ &< 0, \end{aligned} \tag{45}$$

we determine that $g_A(\chi) < 0$ for $\chi > 1$.

Otherwise, if $e_2 < 0$, we immediately have $0 < q_4 < h_A(q_2)$ and $q_a < q_2 < 1$.

Further calculation shows that

$$g_A(1) = e_2 + e_1 + e_0 = (T-S)^2 q_2 \Gamma(q_4), \tag{46}$$

of which the last factor on the right-hand side

$$\Gamma(q_4) = [(R-S)q_2 - (2R-T-S)]q_4 - (R-P)(1-q_2) \tag{47}$$

takes its maximum at $q_4 = 0$ or $q_4 = h_A(q_2)$.

Moreover, we have

$$2e_2 + e_1 < e_1 = 2[(P-S)q_2 + (T+S-2P)]\gamma(q_4), \tag{48}$$

of which the last factor on the right-hand side

$$\gamma(q_4) = [(T-P)(R-S)q_2 - (T-S)(R-P)]q_4 - (R-P)(P-S)(1-q_2) \tag{49}$$

also takes its maximum at $q_4 = 0$ or $q_4 = h_A(q_2)$.

Now we consider the following three different cases.

(i) $e_{21} > 0 > e_{20}$ and $e_{21} + e_{20} > 0$, under which $h_A(q_2) = -e_{20}/e_{21}$.

We obtain

$$\Gamma(0) = -(R-P)(1-q_2) < 0,$$
$$\Gamma(-\frac{e_{20}}{e_{21}}) = -\frac{(R-P)(R-S)(T+S-2P)(1-q_2)^2[(T+P-2S)q_2+T+S-2P]}{e_{21}}\} < 0, \tag{50}$$

and

$$\gamma(0) = -(R-P)(P-S)(1-q_2) < 0,$$
$$\gamma(-\frac{e_{20}}{e_{21}}) = -\frac{(T-S)^2(R-P)(R-S)(T+S-2P)q_2(1-q_2)^2}{e_{21}}\} < 0. \tag{51}$$

(ii) $e_{21} > 0 > e_{20}$ and $e_{21} + e_{20} \leq 0$, under which $h_A(q_2) = 1$.
Notice that now $-\frac{e_{20}}{e_{21}} \geq 1$. Therefore,

$$\Gamma(1) = (2R-P-S)q_2 - (R-P) - (2R-T-S) \leq \max\{\Gamma(0), \Gamma(-\tfrac{e_{20}}{e_{21}})\} < 0, \tag{52}$$

and

$$\gamma(1) = [(T-S)(R-S)-(P-S)^2]q_2 - (R-P)(T+P-2S) \leq \max\{\gamma(0), \gamma(-\tfrac{e_{20}}{e_{21}})\} < 0. \tag{53}$$

(iii) $e_{21} \leq 0$ and $e_{20} < 0$, under which $h_A(q_2) = 1$.
Consider the corresponding range of q_2 in this case. Routine calculation shows that

$$e_{21}(\tfrac{2R-T-S}{R-S}) = \tfrac{(T-R)(T-S)(T+S-2P)(2R-T-S+R-P)}{R-S} > 0, \tag{54}$$

$$e_{21}(\tfrac{(T-S)(R-P)}{(T-P)(R-S)}) = \tfrac{(T-R)(T-S)^3(R-P)(T+S-2P)}{(T-P)^2(R-S)} > 0, \tag{55}$$

and

$$\begin{aligned} -\frac{w_1}{2w_2} &= -\frac{(T-P)^2(T+P-2R)-(P-S)^3}{2(R-S)(P-S)^2} < -\frac{(P-S)^2(T+P-2R)-(P-S)^3}{2(R-S)(P-S)^2} \\ &= \frac{2R-T-S}{2(R-S)} < \frac{2R-T-S}{R-S} < \frac{(T-S)(R-P)}{(T-P)(R-S)}. \end{aligned} \tag{56}$$

The last inequality holds as

$$\tfrac{(T-S)(R-P)}{(T-P)(R-S)} - \tfrac{2R-T-S}{R-S} = \tfrac{(T-R)(T+S-2P)}{(T-P)(R-S)}. \tag{57}$$

We determine that q_2 has to be less than these two values where e_{21} is evaluated. Therefore, both $\Gamma(q_4)$ and $\gamma(q_4)$ are decreasing functions of q_4 and are negative for $0 < q_4 \leq 1$.

To conclude, $e_2 < 0$ always implies $g_A(1) < 0$ and $2e_2 + e_1 < 0$ (that is, $-e_1/2e_2 < 1$). As a result, we have $g_A(\chi) < 0$ for $\chi > 1$.

4.2 Case II: $T + S = 2P$

The derivative of s_X has been given in Equation 20, the sign of which is decided by d_{A0}.

Solving $d_{A0} < 0$, we obtain $0 < q_2 < 1$ and

$$0 < q_4 \begin{cases} \leq 1, & 0 < q_2 < \frac{3(2R-T-S)}{4R-T-3S} \\ < \frac{(1-q_2)(2R-T-S)}{2[(R-S)q_2-(2R-T-S)]}. & \frac{3(2R-T-S)}{4R-T-3S} \leq q_2 < 1 \end{cases} \tag{58}$$

It is the same as the inequality in Table S2. An example is given in Figure S8.

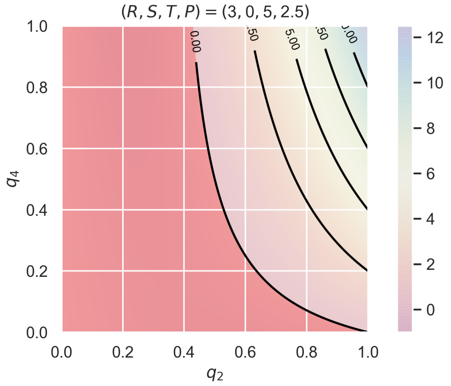

Figure S8: Contour curves of $e_2 = g_A(1)/4 = (T-S)^2 q_2 d_0/8$ for the general IPD game with $T + S = 2P$. Here we have $(R, S, T, P) = (3, 0, 5, 2.5)$. The red region is where $e_2 \leq 0$, or equivalently, $g_A(1) \leq 0$.

4.3 Case III: $T + S < 2P$

The set of q_2 and q_4 is:

$$\begin{cases} 0 \leq q_2 < 1, \\ 0 < q_4 \leq h_A(q_2), \end{cases} \tag{59}$$

where

$$h_A(q_2) = \begin{cases} 1, & 0 \leq q_2 < \frac{(R-P)+(2R-T-S)}{2R-P-S} \\ \frac{(R-P)(1-q_2)}{(R-S)q_2-(2R-T-S)}. & \frac{(R-P)+(2R-T-S)}{2R-P-S} \leq q_2 < 1 \end{cases} \tag{60}$$

The visualization (the explicit expression of $g_A(1) \leq 0$) is given in Figure S9.

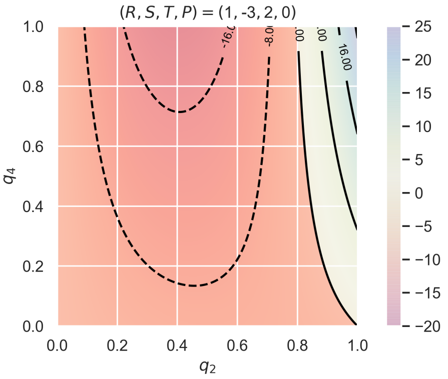

Figure S9: Contour curves of $g_A(1)$ for the general IPD game with $T+S<2P$. Here we have $(R,S,T,P)=(1,-3,2,0)$. The red region is where $g_A(1)\le 0$.

We now show that $g_A(1) \le 0$ is the necessary and sufficient condition for ZD's payoff to be monotonically decreasing with respect to χ.

Recall Equation 19. If $q_2=0$, we immediately have $\frac{ds_X}{d\chi}<0$.

Otherwise, the sign of $\frac{ds_X}{d\chi}$ is decided by $g_A(\chi)$ while $g_A(1)\le 0$ is a necessary condition for s_X to decrease. It suffices to show that $g_A(1)\le 0$, or equivalently, $\Gamma(q_4)\le 0$ is also a sufficient condition.

Here, we let

$$2e_2+e_1=2(T-S)q_2\gamma(q_4), \tag{61}$$

of which the last factor on the right-hand side is

$$\gamma(q_4)=[(R-S)(P-S)q_2-(T-S)(R+P-T-S)]q_4-(T-P)(R-P)(1-q_2). \tag{62}$$

Notice that

$$(T-P)\Gamma-\gamma=(R-S)(2P-T-S)(1-q_2)q_4>0. \tag{63}$$

We get $\gamma<0$.

On the other hand, since

$$\frac{(T-P)^2q_2\Gamma-e_2}{(2P-T-S)(1-q_2)}=(T-S)[(R-S)q_2+R-P]q_4+(R-P)(P-S)(1-q_2)>0, \tag{64}$$

we have $e_2<0$.

To conclude, $g_A(1)\le 0$ always implies $e_2<0$ and $2e_2+e_1<0$. As a result, we have $g_A(\chi)<0$ for $\chi>1$.

5 Class B of unbending strategies

The second class is located in the subspace where $q_2=q_3=0$. The extortioner's payoff s_X is a rational function of degree 1. We have

$$
\begin{aligned}
s_X &= \frac{(T-S)(T+S-2P)q_4\chi}{f_B(\chi)} + P, \\
\frac{ds_X}{d\chi} &= \frac{(T-S)(T+S-2P)[(T-S)q_4+P-S]q_4\chi}{f_B^2(\chi)},
\end{aligned} \tag{65}
$$

where $f_B(\chi) = [(T-S)q_4+T-P]\chi+(T-S)q_4+P-S$. Apparently, $f_B(\chi) > 0$ for $\chi > 1$.

Further, we can draw the following conclusions.

(i) If $T+S > 2P$, s_X is greater than P and increases with χ.

(ii) If $T+S = 2P$, $s_X = P$.

(iii) If $T+S < 2P$, s_X is less than P and decreases with χ. The necessary and sufficient condition q_1 and q_4 need to satisfy is simply

$$
\begin{cases} 0 \le q_1 \le 1, \\ 0 < q_4 \le 1. \end{cases} \tag{66}
$$

6 Class C of unbending strategies

The third class requires $q_1 = q_2 = q_3$. The extortioner's payoff s_X is also a rational function of degree 1. Now we have

$$
\begin{cases} s_X = \frac{(T-S)a_{C0}q_4\chi}{f_C(\chi)} + P, \\ \frac{ds_X}{d\chi} = \frac{(T-S)a_{C0}d_{C0}q_4}{f_C^2(\chi)}, \end{cases} \tag{67}
$$

where $f_C(\chi) = d_{C1}\chi + d_{C0}$ and

$$
\begin{cases} a_{C0} = (R+P-T-S)q_1+T+S-2P = (R-P)q_1+(T+S-2P)(1-q_1), \\ d_{C1} = [(T-S)-(T-R)q_1]q_4+[(T-P)-(T-R)q_1](1-q_1), \\ d_{C0} = [(T-S)-(R-S)q_1]q_4+[(P-S)-(R-S)q_1](1-q_1). \end{cases} \tag{68}
$$

Given that

$$
\begin{cases} d_{C1} > 0, \\ d_{C1}+d_{C0} = (T-S)[(1-q_1)^2+q_4(2-q_1)] > 0, \end{cases} \tag{69}
$$

we obtain that $f_C(\chi) > 0$ for $\chi > 1$.

The monotonicity of s_X is decided by the sign of $a_{C0}d_{C0}$, both being linear functions of q_4. It is straightforward to get

$$
d_{C0} > 0 \quad \Leftrightarrow \quad \begin{cases} 0 \le q_1 \le q_C, \\ 0 < q_4 \le 1, \end{cases} \text{or} \quad \begin{cases} q_C < q_1 \le 1, \\ h_C(q_1) < q_4 \le 1, \end{cases} \tag{70}
$$

and

$$
d_{C0} < 0 \quad \Leftrightarrow \quad \begin{cases} q_C < q_1 < 1, \\ 0 < q_4 < h_C(q_1). \end{cases} \tag{71}
$$

Here

$$q_C = \frac{P-S}{R-S}, \qquad h_C(q_1) = \frac{[(R-S)q_1-(P-S)](1-q_1)}{(T-S)-(R-S)q_1}. \tag{72}$$

On the other hand, the sign of a_{C0} is dependent on that of $T+S-2P$. We now discuss it in detail.

6.1 Case I: $T+S>2P$

Give that a_{C0} is always positive, the sign of $a_{C0}d_{C0}$ is the same as that of d_{C0}. Hence the set of q_1 and q_4 is just

$$\begin{cases} q_C < q_1 < 1, \\ 0 < q_4 < h_C(q_1). \end{cases} \tag{73}$$

We can visualize the result in Figure S10.

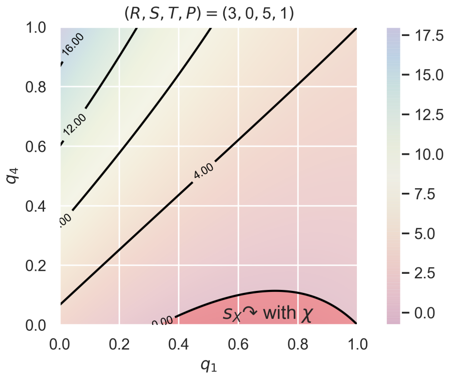

Figure S10: Contour curves of $a_{C0}d_{C0}$ for the general IPD game with $T+S>2P$. Here we use $(R,S,T,P)=(3,0,5,1)$. The red region is where $a_{C0}d_{C0} \le 0$, or equivalently, $d_{C0} \le 0$.

6.2 Case $T+S=2P$

In this case, a_{C0} is positive except when $q_1=0$. We obtain the same set of q_1 and q_4 as Equation 73. To be more specific,

$$q_C = \frac{T-S}{2(R-S)}, \qquad h_C(q_1) = \frac{[2(R-S)q_1-(T-S)](1-q_1)}{2[(T-S)-(R-S)q_1]}. \tag{74}$$

The visualization is given in Figure S11 and we may zoom in to observe the region more closely.

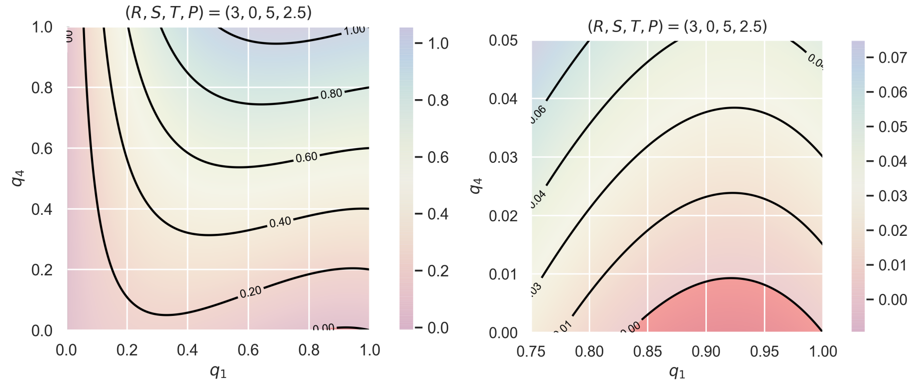

(a) Original (b) Zoomed

Figure S11: Contour curves of $a_{C0}d_{C0}$ for the general IPD game with $T + S = 2P$. Here we use $(R, S, T, P) = (3, 0, 5, 2.5)$. The red region is where $a_{C0}d_{C0} \leq 0$.

6.3 Case III: $T + S < 2P$

The sign of a_{C0} now depends on the value of q_2. It is trivial to get

$$\begin{cases} a_{C0} < 0, & 0 \leq q_1 < q_c, \\ a_{C0} > 0, & q_c < q_1 \leq 1 \end{cases} \tag{75}$$

where $q_c = (2P - T - S)/(R + P - T - S)$. Notice that

$$q_C - q_c = \frac{(T-P)(R-P)}{(R-S)(R+P-T-S)} > 0. \tag{76}$$

To guarantee that the product of a_{C0} and d_{C0} is negative, we need

$$\begin{cases} 0 \leq q_1 < q_c, \\ 0 < q_4 \leq 1, \end{cases} \quad \text{or} \quad \begin{cases} q_C < q_1 < 1, \\ 0 < q_4 < h_C(q_1). \end{cases} \tag{77}$$

An example is given in Figure S12.

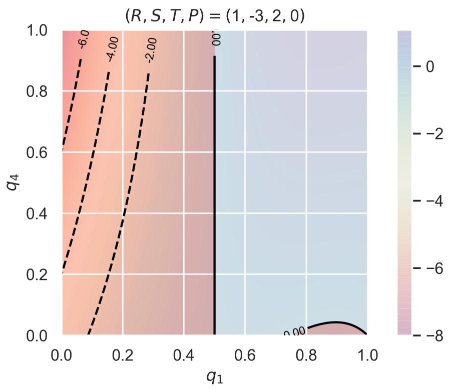

Figure S12: Contour curves of $a_{C0}d_{C0}$ for the general IPD game with $T+S<2P$. Here we use $(R,S,T,P)=(1,-3,2,0)$. The two red regions are where $a_{C0}d_{C0}\leq 0$.

7 Class D of unbending strategies

The last class requires $q_4 = h_D(q_1,q_2,q_3) = a_{D0}/(2R-T-S)$, under which we obtain

$$\begin{cases} s_X = \frac{(T-S)a_{D0}\chi}{f_D(\chi)} + P, \\ \frac{ds_X}{d\chi} = \frac{(T-S)a_{D0}d_{D0}}{f_D^2(\chi)}. \end{cases} \tag{78}$$

Here, $f_D(\chi) = d_{D1}\chi + d_{D0}$ and

$$\begin{cases} a_{D0} = -(T+S-2P)q_1 + (R-P)(q_2+q_3) + T + S - R - P, \\ d_{D1} = -(T-S)q_1 + (T-R)q_2 + (R-S)q_3 + R - S, \\ d_{D0} = -(T-S)q_1 + (R-S)q_2 + (T-R)q_3 + T - R. \end{cases} \tag{79}$$

Simple calculation shows that

$$\begin{cases} (R-P)d_{D1} - (T-R)a_{D0} = (2R-T-S)[(T-P)(1-q_1) + (R-P)q_3] \geq 0, \\ (R-P)(d_{D1}+d_{D0}) - (T-S)a_{D0} = (T-S)(2R-T-S)(1-q_1) \geq 0. \end{cases} \tag{80}$$

Further, $0 < q_4 \leq 1$ implies $0 < a_{D0} \leq 2R-T-S$. We conclude that $f_D(\chi) > 0$ for $\chi > 1$.

Likewise, to determine the monotonicity of s_X, we need to figure out the sign of $a_{D0}d_{D0}$. The factor d_{D0} should be negative for $ds_X/d\chi$ to be negative. Moreover, routine calculation yields

$$(R-S)[(2R-T-S) - a_{D0}] + (R-P)d_{D0} = (2R-T-S)[(P-S)(1-q_1) - (R-P)q_3 + (R-S)] > 0. \tag{81}$$

In other words, if $d_{D0} < 0 < a_{D0}$, we will always have $a_{D0} \leq 2R-T-S$. Therefore, the implicit condition for s_X to be a decreasing function is

$$d_{D0} < 0 < a_{D0}. \tag{82}$$

Notice that both $a_{D0} = 0$ and $d_{D0} = 0$ take the form of the equation of a plane in the 3-dimensional space and the above inequality corresponds to the space between the two planes whilst inside a unit cube.

We list a few critical points on the two planes as well as their line of intersection.

- $a_{D0} = 0$: $(1, 1, 0)$, $(1, 0, 1)$.
- $d_{D0} = 0$: $(1, 1, 0)$, $(0, 0, -1)$.
- $d_{D0} = a_{D0} = 0 : \begin{bmatrix} x \\ y \\ z \end{bmatrix} = \begin{bmatrix} R-P \\ T-P \\ -(P-S) \end{bmatrix} t + \begin{bmatrix} 1 \\ 1 \\ 0 \end{bmatrix}$.

The projection of their intersection onto the plane $q_3 = 1$ is $(T-P)q_1 - (R-P)q_2 - (T-R) = 0$.

For ease of notation, we use the implicit expression in Equation 82 together with the default restrictions on the probabilities to represent the set of q_1, q_2, and q_3:

$$\begin{cases} 0 \le q_1, q_2, q_3 \le 1, \\ d_{D0} < 0 < a_{D0}, \end{cases} \tag{83}$$

where a_{D0} and d_{D0} can also be rewritten as

$$\begin{cases} a_{D0} = (T+S-2P)(1-q_1) - (R-P)(1-q_2) + (R-P)q_3, \\ d_{D0} = (T-S)(1-q_1) - (R-S)(1-q_2) + (T-R)q_3. \end{cases} \tag{84}$$

7.1 Case I: $T + S > 2P$

An example of the possible set of q_1, q_2, and q_3 is given in Figure S13. A point (q_1, q_2, q_3) in the 3-dimensional space, lying between the two planes $a_{D0} = 0$ and $d_{D0} = 0$, corresponds to the strategy $\boldsymbol{q} = (q_1, q_2, q_3, h_D(q_1, q_2, q_3))$.

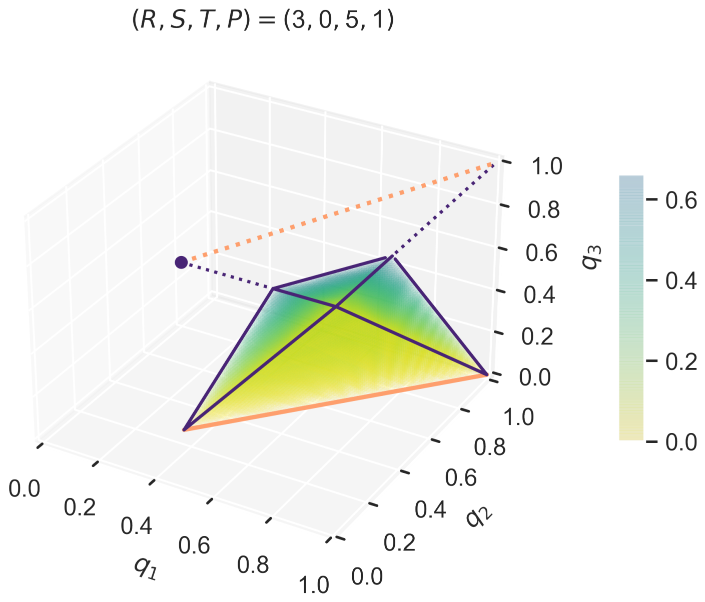

Figure S13: Set of points satisfying $d_{D0} < 0 < a_{D0}$. The color map indicates the value of q_4. The intersection of the two planes $a_{D0} = 0$ and $d_{D0} = 0$ is colored in pink. The projection of the set onto the plane $q_3 = 1$ is marked by dashed lines. In addition, the x-component of the navy point corresponds to the lower bound of q_1. Here, we use $(R, S, T, P) = (3, 0, 5, 1)$.

7.2 Case II: $T + S = 2P$

The visualization of the set of q_1, q_2, and q_3 is given in Figure S14.

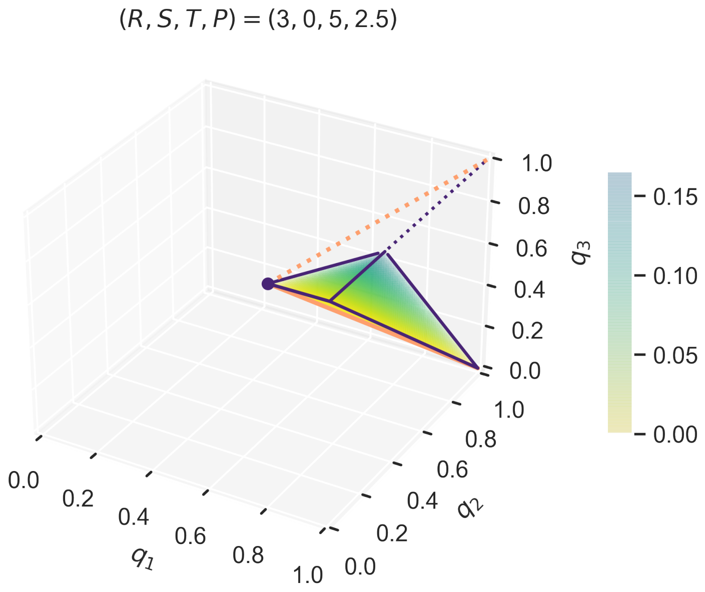

Figure S14: Set of points satisfying $d_{D0} < 0 < a_{D0}$. The color map indicates the value of q_4. The intersection of the two planes $a_{D0} = 0$ and $d_{D0} = 0$ is colored in pink. The projection of the set onto the plane $q_3 = 1$ is marked by dashed lines. In addition, the x-component of the navy point corresponds to the lower bound of q_1. Here, we use $(R, S, T, P) = (3, 0, 5, 2.5)$.

7.3 Case III: $T + S < 2P$

For the last case, an example is given in Figure S15.

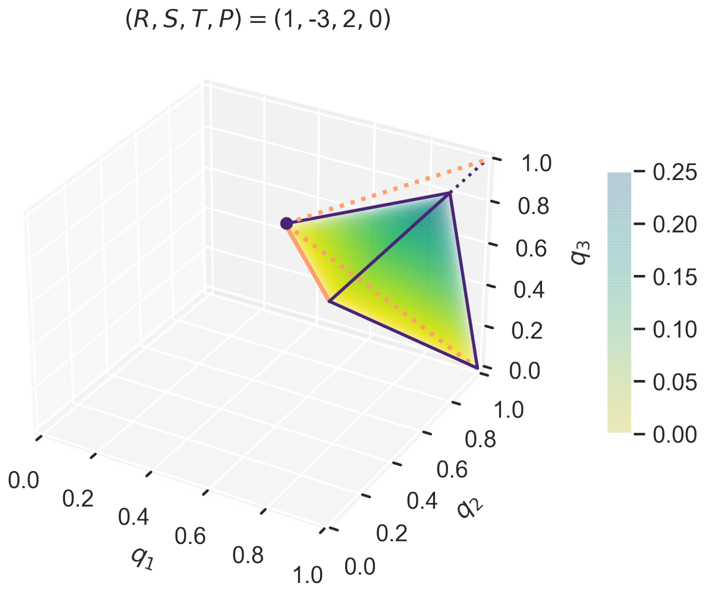

Figure S15: Set of points satisfying $d_{D0} < 0 < a_{D0}$. The color map indicates the value of q_4. The intersection of plane $a_{D0} = 0$ and plane $d_{D0} = 0$ (dashed line) and that of $a_{D0} = 0$ and $q_3 = 1$ (solid line) are both colored in pink. The projection of the set onto the plane $q_3 = 1$ is marked by dashed lines. In addition, the x-component of the navy point corresponds to the lower bound of q_1. Here, we use $(R, S, T, P) = (1, -3, 2, 0)$.

8 The impact of unbending strategies on extortionate ZD strategies: dominance, average payoffs, and best response

The four classes of unbending strategies have been comprehensively studied so far. We further answer three significant questions in the scenario where X plays an extortionate ZD strategy and Y plays an unbending strategy:

- Is X's payoff s_X always greater than the punishment P?
- Is X's payoff s_X always greater than Y's payoff s_Y?
- Does Y's payoff s_Y always decrease with respect to χ?

8.1 Comparing s_X and P

Class	s_X	a_i
A	$\frac{(T-S)(R-P)q_4\chi(a_{A1}\chi+a_{A0})}{f_A(\chi)}+P$	$a_{A1}=(P-S)q_2+T+S-2P$ $a_{A0}=(T-P)q_2-(T+S-2P)$
B	$\frac{(T-S)a_{B0}q_4\chi}{f_B(\chi)}+P$	$T+S-2P$
C	$\frac{(T-S)a_{C0}q_4\chi}{f_C(\chi)}+P$	$(R-P)q_1+(T+S-2P)(1-q_1)$
D	$\frac{(T-S)a_{D0}\chi}{f_D(\chi)}+P$	$(T+S-2P)(1-q_1)-(R-P)(1-q_2)+(R-P)q_3$

Table S8: Payoff of an extortionate ZD strategy playing against an unbending strategy. The expressions of $f_A(\chi)$, $f_B(\chi)$, $f_C(\chi)$, and $f_D(\chi)$ have been given previously and we have verified that they are all positive.

Table S8 lists the expressions of s_X when playing against unbending strategies from different classes. The sign of $s_X - P$ is identical to that of a_0 or $a_1\chi + a_0$. It is worth mentioning that for Class A, if we let $q_2 = 0$, the expression can be further simplified as

$$\frac{(T-S)(T+S-2P)q_4\chi}{[(T-S)q_4+(T-P)]\chi+(T-S)q_4+(P-S)}+P. \tag{85}$$

After some routine calculation, we present the possible signs of $s_X - P$ in Table S9. Besides, given the linear relation in Equation 5, we know that $s_X - P$ and $s_Y - P$ always share the same sign. It is clear from the table that s_X is greater than P under most circumstances yet the opposite may happen well.

Class \ Case	$T+S>2P$	$T+S=2P$	$T+S<2P$
A	$+$	$+$	$\begin{cases} -, & q_2=0 \\ + \to -, & 0<q_2<q_A \\ +. & q_A \le q_2 < 1 \end{cases}$
B	$\emptyset$	$\emptyset$	$-$
C	$+$	$+$	$\begin{cases} -, & 0 \le q_1 < q_c \\ +. & q_C < q_1 < 1 \end{cases}$
D	$+$	$+$	$+$

Table S9: Sign of $s_X - P$ ($s_Y - P$ or $s_X - s_Y$) as χ increases from 1 to $+\infty$. Here, $q_A = (2P-T-S)/(P-S)$.

8.2 Comparing s_X and s_Y

Notice that the linear relation in Equation 5 also implies that $s_X - P > 0$ is equivalent to $s_X > s_Y$. Therefore, from Table S9, we conclude that the extortioner's payoff is likely to be less than its opponent's payoff if $T + S < 2P$. More precisely, the IPD game needs to meet one of the following four conditions:

(i) the opponent plays a strategy from Class A with $q_2 = 0$ (a subset of Class B),

(ii) the opponent plays a strategy from Class A with $0 < q_2 < q_A$ and the extortion factor $\chi > -a_{A0}/a_{A1}$,

(iii) the opponent plays a strategy from Class B, or

(iv) the opponent plays a strategy from Class C with $0 \le q_1 < q_c$.

8.3 Monotonicity of s_Y

For the completeness of our study, we also discuss the monotonicity of s_Y. The expressions of s_Y are similar to those in Table S8 and we only need to remove χ from the numerators.

Recall that if s_X decreases with χ and $s_X - P > 0$, s_Y will decrease with χ as well (once again, we use the linear relation). Now consider the four special circumstances from the last section.

For (i), we get

$$\frac{ds_Y}{d\chi} = -\frac{(T-S)(T+S-2P)[(T-S)q_4+(T-P)]q_4}{\{[(T-S)q_4+(T-P)]\chi+(T-S)q_4+(P-S)\}^2}. \tag{86}$$

It follows immediately that s_Y increases with respect to χ for $\chi > 1$.

For (ii), we have

$$s'_Y = \frac{(T-S)(R-P)q_4(-a_{A1}d_{A2}\chi^2-2a_{A0}d_{A2}\chi+a_{A1}d_{A0}-a_{A0}d_{A1})}{f_A^2(\chi)}. \tag{87}$$

Given that $a_{A1} < 0$ and $d_{A2} > 0$, the last factor of the numerator is a quadratic function opening upwards. Additionally, we evaluate its value at $\chi = 1$ and get

$$-(T-S)^2 q_2\{[(T-R)q_2 + (2R-T-S)]q_4 + (R-P)(1-q_2)\} < 0. \tag{88}$$

Hence s_Y first decreases then increases with χ for $\chi > 1$.

As to (iii) and (iv), notice that $s_Y - P$ is hyperbolic with a negative numerator and a positive denominator. It is straightforward to show that s_Y is an increasing function of χ.

We summarize the above results in Table S10.

<table>
<tr><th>Class \ Case</th><th>$T+S>2P$</th><th>$T+S=2P$</th><th>$T+S<2P$</th></tr>
<tr><td>A</td><td>$\searrow$</td><td>$\searrow$</td><td>$\begin{cases} \nearrow, & q_2 = 0 \\ \searrow\to\nearrow, & 0 < q_2 < q_A \\ \searrow. & q_A \le q_2 < 1 \end{cases}$</td></tr>
<tr><td>B</td><td>$\emptyset$</td><td>$\emptyset$</td><td>$\nearrow$</td></tr>
<tr><td>C</td><td>$\searrow$</td><td>$\searrow$</td><td>$\begin{cases} \nearrow, & 0 \le q_1 < q_c \\ \searrow. & q_C < q_1 < 1 \end{cases}$</td></tr>
<tr><td>D</td><td>$\searrow$</td><td>$\searrow$</td><td>$\searrow$</td></tr>
</table>

Table S10: Monotonicity of s_Y.

8.4 Maximum values of s_X and s_Y

Now that we have figured out the relationship between s_X and P (s_Y and P) and the monotonicity of s_Y, we can finally identify the maximum values of the two payoffs s_X and s_Y as well as the corresponding unbending strategies.

Given that s_X is a decreasing function of χ, player X must compromise with its opponent Y by reducing the extortion factor χ to 1 to get the optimal payoff, which is given in Table S11. We also have $s_X(1) = s_Y(1)$.

Class	$s_X(1)$ and $s_Y(1)$	
A	R	
B	$q_1 = 1$: R	$0 \le q_1 < 1$: $\frac{(T+S-2P)q_4}{2q_4+1} + P$
C	$\frac{[(R-P)q_1+(T+S-2P)(1-q_1)]q_4}{(1-q_1)^2+(2-q_1)q_4} + P$	
D	$\frac{(T+S-2P)(1-q_1)-(R-P)(1-q_2)+(R-P)q_3}{2(1-q_1)-(1-q_2)+q_3} + P$	

Table S11: Payoff of the fair extortioner (or its opponent) with extortion factor $\chi \to 1$.

Furthermore, $s_X(1)$ can be maximized if player Y adopts a proper strategy. After some calculation, we present the final results in Table S12.

Class	Maximum of $s_X(1)$	Opponent's strategy q
A	R	$(1, q_2, 0, q_4)$
B	R	$(1, 0, 0, q_4)$
C	$\lim_{\delta \to 0} \frac{[(R-P)(1-\delta)+(T+S-2P)\delta]q_4}{\delta^2+(1+\delta)q_4} + P = R$	$(1-\delta, 1-\delta, 1-\delta, q_4)$
D	R	$(1, q_2, q_3, \frac{(R-P)(q_2+q_3-1)}{2R-T-S})$

Table S12: Maximum value of $s_X(1)$ and the corresponding unbending strategy. Notice that for Class B and Class C, $s_X(1)$ can get arbitrarily close to the maximum value P (if $0 \le q_1 < 1$) and R. And for any strategy $\boldsymbol{q}$, the four components q_i's still need to satisfy the requirements of being an unbending strategy in Table S2.

8.5 Figures

To sum up, we visualize the two payoffs s_X (Table S13) and s_Y (Table S14) in an IPD game where X plays an extortionate ZD strategy and Y plays an unbending strategy. We also show the parametric curve of (s_X, s_Y) (Table S15), which can be zoomed in for a closer observation (Table S16). All three cases ($T+S>2P$, $T+S=2P$, and $T+S<2P$) are included.

We emphasize the following facts which can be observed directly from these figures.

- Both the two payoffs s_X and s_Y are independent of the parameter ϕ.
- The payoff s_X is greater than the punishment P most of the time. Nevertheless, it may be (i) less than P if the extortion factor χ exceeds the threshold value $-a_{A0}/a_{A1}$, or (ii) always less than P for χ greater than one.
- The "extortioner" is dominant most of the time ($s_X > s_Y$) yet it may be beaten by an unbending strategy ($s_X < s_Y$) if $T+S<2P$ and
  - (i) the opponent plays a strategy from Class A with $q_2 = 0$ (a subset of Class B),
  - (ii) the opponent plays a strategy from Class A with $\begin{cases} \chi > -a_{A0}/a_{A1} \\ 0 < q_2 < q_A \end{cases}$, or
  - (iii) the opponent plays a strategy from Class B, or
  - (iv) the opponent plays a strategy from Class C with $0 \le q_1 < q_c$.
- The payoff s_X is a decreasing function of χ. The other payoff s_Y decreases with χ as well under most circumstances but it may (i) first decreases then increases with χ, or (ii) always increases with χ.
- The relation between $T+S$ and $2P$ determines the shape and convexity of the quadrilateral with four vertices (P,P), (T,S), (R,R) and (S,T) (when

$T + S = 2P$ it degenerates to a triangle, where the vertex (P, P) lies on the edge pointing from (T, S) to (S, T)). if $T + S > 2P$, the quadrilateral is convex and the parametric curve of (s_X, s_Y) stays within it. Whereas if $T + S < 2P$, the quadrilateral is concave and it is possible for a fraction of, or even the entire parametric curve lies outside it.

Class	$T + S > 2P$ $(R, S, T, P) = (3, 0, 5, 1)$	$T + S = 2P$ $(R, S, T, P) = (3, 0, 5, 2.5)$	$T + S < 2P$ $(R, S, T, P) = (1, -3, 2, 0)$
A	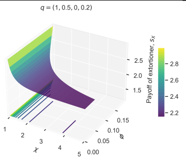	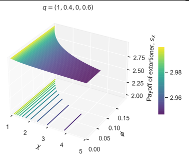	$q_2 < q_A$ $q_2 > q_A$ 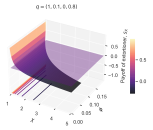 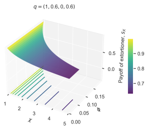
B			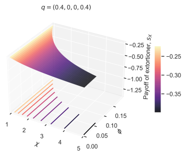
C	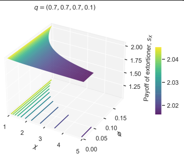	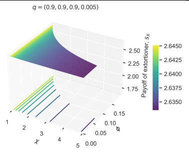	$q_1 < q_c$ $q_1 > q_C$ 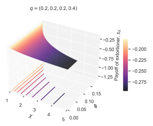 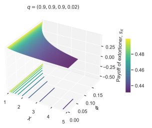
D	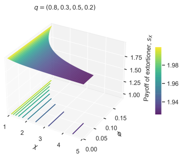	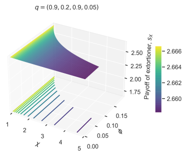	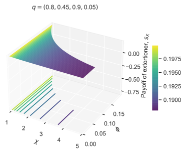

Table S13: Surface of s_X when X uses an extortionate ZD strategy and Y uses an unbending strategy. The contour curves on the xy-plane are also given and the horizontal plane $z = P$ is added for one particular example on the upper right corner. We use two different colormaps depending on whether it is possible for s_X to be less than P. As before, $q_A = (2P - T - S)/(P - S)$.

Class	$T+S>2P$ $(R,S,T,P)=(3,0,5,1)$	$T+S=2P$ $(R,S,T,P)=(3,0,5,2.5)$	$T+S<2P$ $(R,S,T,P)=(1,-3,2,0)$
A	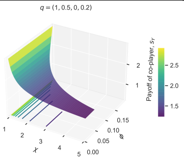	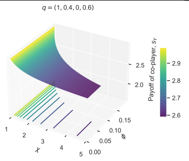	$q_2 < q_A$ $q_2 > q_A$ 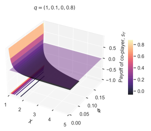 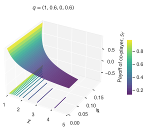
B			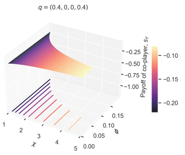
C	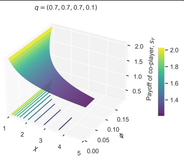	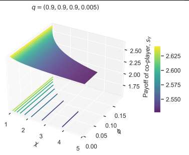	$q_1 < q_c$ $q_1 > q_C$ 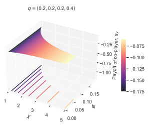 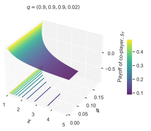
D	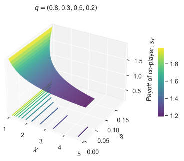	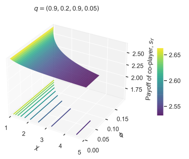	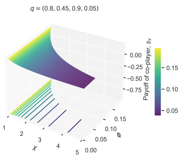

Table S14: Surface of s_Y when X uses an extortionate ZD strategy and Y uses an unbending strategy.

Class	$T+S>2P$<br>$(R,S,T,P)=(3,0,5,1)$	$T+S=2P$<br>$(R,S,T,P)=(3,0,5,2.5)$	$T+S<2P$<br>$(R,S,T,P)=(1,-3,2,0)$
A	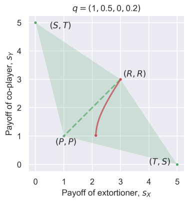	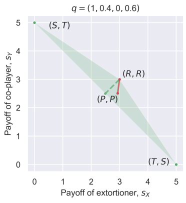	$q_2 < q_A$ $q_2 > q_A$<br>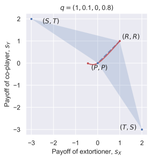 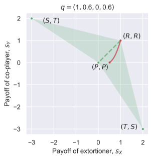
B			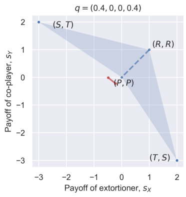
C	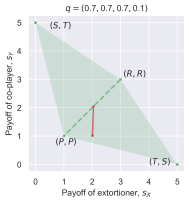	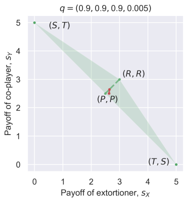	$q_1 < q_c$ $q_1 > q_C$<br>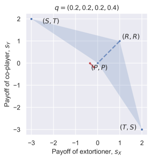 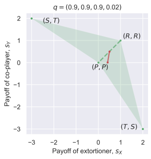
D	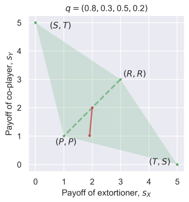	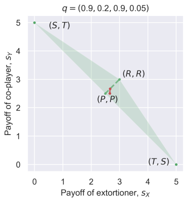	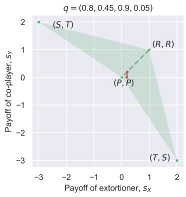

Table S15: Parametric curve of (s_X, s_Y) with variable χ increasing from 1 to $+\infty$. The coordinates of the vertices are the four possible outcomes of the one-shot PD game. The dashed line connects the two vertices (P,P) and (R,R).

Class	$T+S>2P$ $(R,S,T,P)=(3,0,5,1)$	$T+S=2P$ $(R,S,T,P)=(3,0,5,2.5)$	$T+S<2P$ $(R,S,T,P)=(1,-3,2,0)$
A	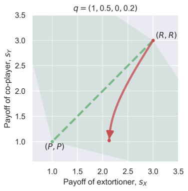	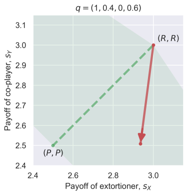	$q_2 < q_A$ $q_2 > q_A$ 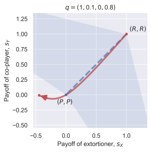 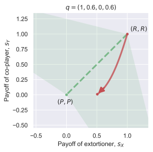
B			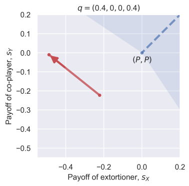
C	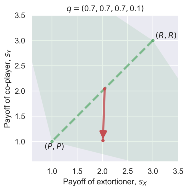	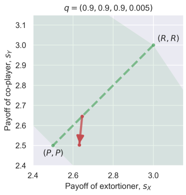	$q_1 < q_c$ $q_1 > q_C$ 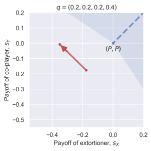 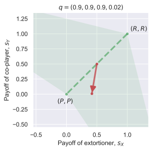
D	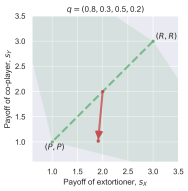	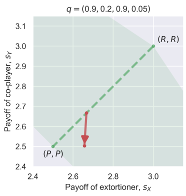	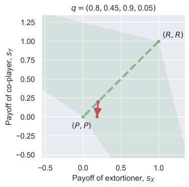

Table S16: Zoom-in view of the parametric curve of (s_X, s_Y) with variable χ increasing from 1 to $+\infty$. The arrow denotes the direction of motion.

9 The complete picture of extortionate ZD's superiority: the need for studying $T+S<2P$

In what follows, we include a few excerpts from the literature where it is believed that an extortioner (using $O=P$ and $\chi>1$) always gets a higher payoff than the punishment P and that of the co-player and (or) its payoff increases with respect to the extortion factor χ. We also attach a few examples which ignore the role of ϕ in shaping the payoffs. As we point out in the main text as well as in the supplementary information here, the superiority of extortionate ZD strategies is typically studied under conventional IPD games where $T+S>2P$, but for the sake of completeness, it is equally important to study the robustness of ZD strategies across Prisoner's Dilemma games even of drastically different nature such as the case of $T+S<2P$, as detailed in our

supplementary information.

[1]

- Under the extortionate strategy, X's score depends on Y's strategy $\boldsymbol{q}$, and both are maximized when Y fully cooperates, with $\boldsymbol{q} = (1, 1, 1, 1)$.

[2]

- Extortion strategies, whose existence Press and Dyson report, grant a disproportionate number of high payoffs to X at Y's expense.

[3]

- In particular, Press and Dyson highlighted a subset of ZD strategies, called "extortion strategies" that grants the extorting player a disproportionately high payoff when employed against a naive opponent who blindly adjusts his strategy to maximize his own payoff.

[4]

- In extortionate games, the strategy being preyed upon can increase their own payoff by modifying their own strategy $\boldsymbol{q}$, but this only increases the extortionate strategy's payoff. As a consequence, Press and Dyson conclude that a ZD strategy will always dominate any opponent that adapts its own strategy to maximize their payoff, for example, by Darwinian evolution.

[5]

- Another subset consists of the extortion strategies, which guarantee that one player's own surplus exceeds the coplayer's surplus by a fixed percentage.
- In this case, player I can guarantee that his or her own "surplus" (over the maximin value P) is the χ-fold of the coplayer's surplus.

[6]

- In this way, a player can manipulate and extort his co-player, thereby ensuring that the own payoff never falls below the co-player's payoff.
- Press and Dyson discovered that certain zero-determinant strategies can guarantee that a player always yields at least the opponent's payoff.
- Such extortioner strategies $\boldsymbol{p}$ guarantee that the player's own surplus (over the maximin value P) exceeds the co-player's surplus by a fixed percentage.

[7]

- In particular, ZD strategies can fix the co-player's payoff to an arbitrary value between P and R; or ensure that the own 'surplus' (over the maximin value P) is twice as large as the co-player's surplus; etc.

[8]

- According to the theory, extortioners demand and receive an excessive share of any surplus, which allows them to outperform any adapting co-player.
- As a result, extortioners are unbeatable: in a pairwise encounter, they cannot be outperformed by any opponent.

[9]

- Among the selfish strategies there are two main types: the "Equalizers" that manage to force a fixed payoff onto the opponent, and the "Extortionists" that fix the relative payoff between player and opponent in such a manner that if the opponent changes its strategy so as to get a better payoff, the extortioner always receives commensurately more.

10 The robustness of extortion ability of general ZD strategies

The introduction of unbending strategies points out the fact that player X may not always get a higher payoff than player Y even if it uses an extortionate ZD strategy with $O = P$ and $\chi > 1$. We can not only extend the above finding to a more general ZD strategy with $P \leq O \leq R$ but also quantify the probability of successful extortion made by a ZD player.

Consider different values of O and χ. We present the results in Figure S16.

On the one hand, the baseline payoff O controls the level of generosity of the ZD player and hence impacts its chance to outperform its co-player. Increasing O above P makes an "extortioner" less likely to be able to ensure the dominance. Noticeably, the payoff structure players an even more pronounced role than does O. For $T + S > 2P$, the curvature is concave downwards and the ZD player is able to maintain dominance for most of the time even using intermediate O values. Yet for $T+S < 2P$, the curvature is concave upwards and the ZD player is more likely to lose its dominance for any $P < O \leq R$.

On the other hand, the extortion factor χ determines the level of extortion of the ZD player. It also plays a non-negligible role in making an "extortioner" behave like actual one. For $T + S > 2P$, a higher χ can help the ZD player be dominant if O is small, whereas the same higher χ can have a counter effect if O is large. As to $T+S < 2P$, the higher χ is, the more likely an extortion relation would backfire, that is, the more likely ZD fails to dominant its co-player.

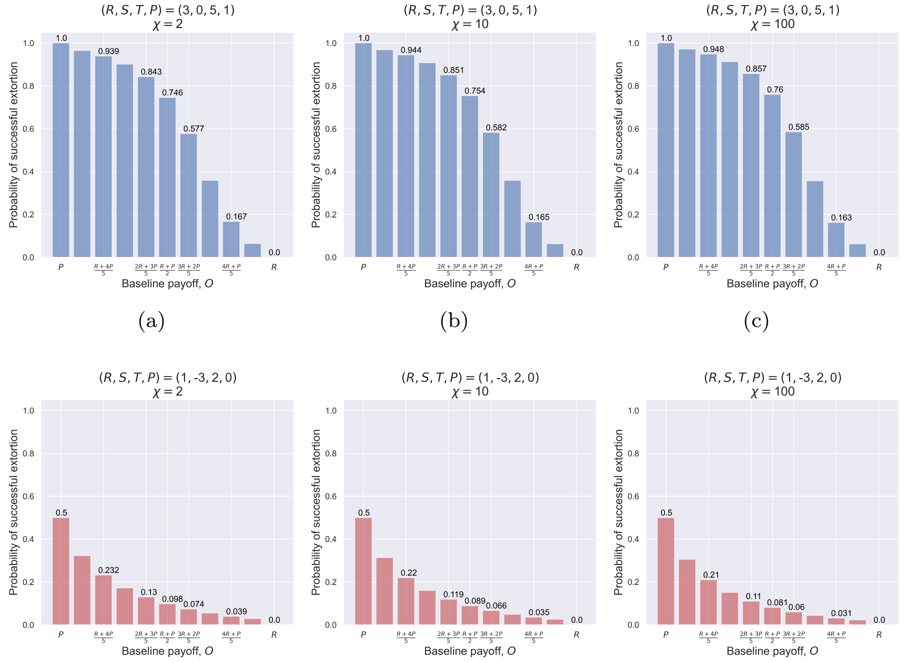

(a) (b) (c)

(d) (e) (f)

Figure S16: Probability of "successful extortion" that player X actually gets a better payoff than its co-player Y ($s_X > s_Y$). The two players use a general ZD strategy and a random strategy uniformly drawn from memory-one strategies $[0, 1]^4$, respectively. The baseline payoff O varies from P to R and the extortion factor χ is set as 2, 10, and 100, respectively.

11 The role played by ϕ (continued)

We have seen previously that tuning the parameter ϕ will impact the dependence of s_X on the extortion factor in a non-trivial way. We further show that when χ is fixed, s_X is a monotonic function of ϕ. It can be told immediately from the expression of s_X, which takes the form of a linear rational function of ϕ. Recall the three strategies we used in Figure S3. As a matter of fact, s_X decreases with respect to ϕ if Y plays $\boldsymbol{q_1}$, increases if Y plays $\boldsymbol{q_2}$, and remains a constant if Y plays Tit-for-Tat. The corresponding curves of s_X are given in Figure S17 for $\chi = 2$. More examples are shown in Figure S18.

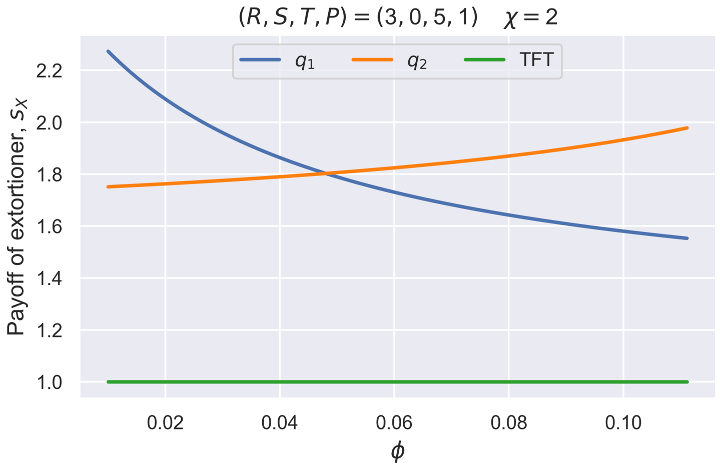

Figure S17: Extortioner's payoff against different strategies. We work on the conventional IPD game and fix χ as 2. Here, $\boldsymbol{q}_1 = (0.05, 0.95, 0.05, 0.1)$ and $\boldsymbol{q}_2 = (0.4, 0.1, 0.9, 0.2)$.

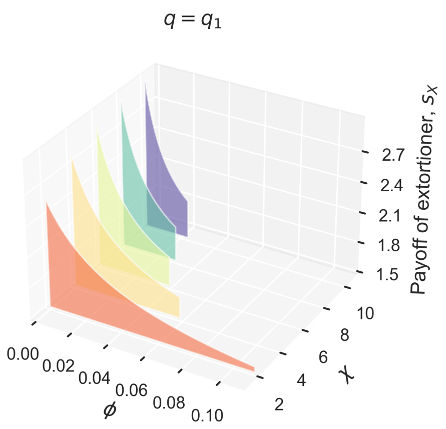

(a) $s_X(\boldsymbol{q}_1, \chi, \phi)$

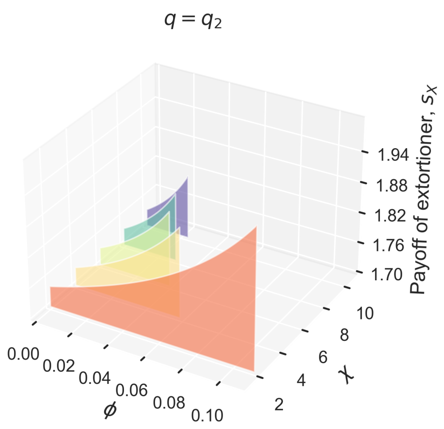

(b) $s_X(\boldsymbol{q}_2, \chi, \phi)$

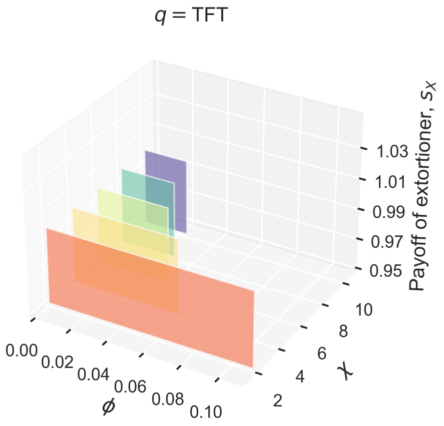

(c) $s_X(\mathrm{TFT}, \chi, \phi)$

Figure S18: Waterfall plots of s_X when playing against different strategies for fixed values of χ. We work on the conventional IPD game. Different colors correspond to different values of χ (2, 4, 6, 8, and 10). As before, $\boldsymbol{q}_1 = (0.05, 0.95, 0.05, 0.1)$ and $\boldsymbol{q}_2 = (0.4, 0.1, 0.9, 0.2)$.

12 Steering learning dynamics from extortion to fairness and cooperation: reactive strategies

From now on, we work on the donation game where the four payoffs satisfies $(R, S, T, P) = (b-c, -c, b, 0)$, and $r = b/c$ is the benefit-to-cost ratio. According to the definition, a strategy $\boldsymbol{p} = (p_1, p_2, p_3, p_4)$ is reactive if $p_1 = p_3$ and $p_2 = p_4$. In the donation game, a general ZD strategy (see Equation 3) becomes a reactive strategy if $\phi = 1/(b\chi + 1)$. That is, reactive strategies is a subset of general ZD

strategies. As a matter of fact, let

$$\chi \geq 1 \qquad \text{or} \qquad \chi \leq \chi^{\text{upper}} = \min\{-\frac{b-O}{O+c}, -\frac{O+c}{b-O}\}. \tag{89}$$

We can divide the region of reactive strategies into two parts, each of which is a subset of general ZD strategies. The proof is trivial. Two examples are sketched in Figure S19.

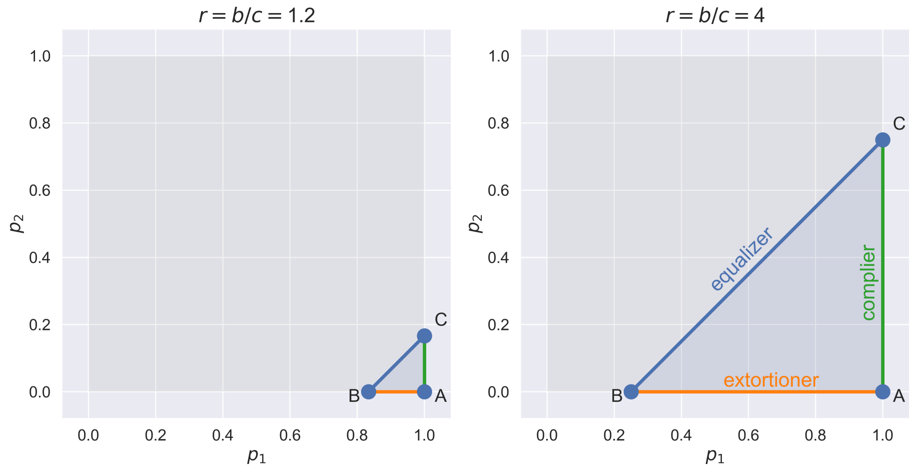

(a) $r = 1.2$

(b) $r = 4$

Figure S19: Reactive strategies as a subset of general ZD strategies. Assume that $\phi = 1/(b\chi + 1)$. In each panel, the set of reactive strategies is shaded in gray (the unit square), that of ZD strategies with positive χ is in blue (the right triangle), and that of ZD strategies with negative χ is in gray (the pentagon, which "=" the unit square "−" the triangle). Moreover, the three particular types of ZD strategies: extortioner, complier, and equalizer are highlighted in orange, green, and blue, respectively.

Given the value of ϕ, we can update the expressions for p_1 and p_2:

$$\begin{cases} p_1 = 1 - \frac{(b-c-O)(\chi-1)}{b\chi+c}, \\ p_2 = \frac{O(\chi-1)}{b\chi+c}. \end{cases} \qquad 0 \leq O \leq b-c \tag{90}$$

The two components satisfy a linear relation

$$O(1-p_1) - (b-c-O)p_2 = 0. \tag{91}$$

Further, for a few particular types of ZD strategies, their expressions can be obtained by letting the baseline payoff O and (or) the extortion factor χ be some specific values. The results are summarized in Table S17. Notice that the generous Tit-for-Tat (GTFT) strategy for the donation game is $(1, \delta, 1, \delta)$, where

$$\delta = \min\{1 - \frac{T-R}{R-S}, \frac{R-P}{T-P}\} = \min\{\frac{b-c}{b}, \frac{b-c}{b}\} = \frac{b-c}{b}. \tag{92}$$

Therefore, for a generous ZD strategy, if $O = b - c$ and $\chi \to \infty$, or equivalently, $p_1 = 1$ and $p_2 = 1 - c/b$, it would become GTFT.

Type	Parameters	Expressions	Remarks
extortionate ZD (extortioner)	$O = P = 0$ $\chi \geq 1$	$\begin{cases} p_1 = 1 - \frac{(b-c)(\chi-1)}{b\chi+c}, \\ p_2 = 0. \end{cases}$	$\frac{c}{b} \leq p_1 \leq 1$
generous ZD (complier)	$O = R = b - c$ $\chi \geq 1$	$\begin{cases} p_1 = 1, \\ p_2 = \frac{(b-c)(\chi-1)}{b\chi+c}. \end{cases}$	$0 \leq p_2 \leq 1 - \frac{c}{b}$
equalizer	$0 \leq O \leq b - c$ $\chi = +\infty$	$\begin{cases} p_1 = \frac{O+c}{b}, \\ p_2 = \frac{O}{b}. \end{cases}$	$p_1 - p_2 = \frac{c}{b}$
mediocre ZD	$O = \frac{T+S}{2} = \frac{b-c}{2}$ $\chi \geq 1$	$\begin{cases} p_1 = 1 - \frac{(b-c)(\chi-1)}{2(b\chi+c)}, \\ p_2 = \frac{(b-c)(\chi-1)}{2(b\chi+c)}. \end{cases}$	$p_1 + p_2 - 1 = 0$
fair ZD (Tit-for-Tat)	$\chi = 1$	$\begin{cases} p_1 = 1, \\ p_2 = 0. \end{cases}$	$s_X = s_Y$

Table S17: Different types of ZD strategies in the donation game.

To study extortion and fairness in reciprocal actions, we have considered the scenario where player X uses an extortionate ZD strategy and player Y uses an unbending strategy. To further extend previous results, we now assume that player X uses a reactive strategy in the donation game where X's learning dynamics (payoff optimization through exploring the entire strategy space) is under influence of co-player Y using a fixed unbending strategy from class A or class D.

13 Under influence of class A of unbending strategies (continued)

Let $\boldsymbol{p} = (p_1, p_2, p_1, p_2)$ (a reactive strategy) and $\boldsymbol{q} = (1, q_2, 0, q_4)$ (an unbending strategy from Class A), where q_2 and q_4 satisfy

$$\begin{cases} \frac{c(b-c)}{b^2+bc-c^2} = q_a < q_2 < 1, \\ 0 < q_4 \leq h_A(q_2) = \frac{(b-c)(1-q_2)[(b^2+bc-c^2)q_2-(b-c)c]}{bc^2q_2^2-(b-c)(b^2-bc-c^2)q_2+(b-c)^2(b+c)}. \end{cases} \tag{93}$$

We obtain $s_X(p_1, p_2)$ as a quadratic rational function of p_1 and p_2. More specifically, we have

$$s_X(p_1, p_2) - (b - c) = \frac{(1 - p_1)\Delta(p_1, p_2)}{f_A(p_1, p_2)}, \tag{94}$$

where

$$\Delta(p_1, p_2) = [bq_2q_4 + c(1 - q_2) - (b - c)q_4](1 - p_2) - b(1 - q_2)(q_4p_1 + 1 - q_4), \tag{95}$$

and $f_A(p_1, p_2) = (1 - q_2)[(1 - p_1) - q_4(p_2 - p_1)^2] + q_4(1 - p_2)$, which is always positive.

In particular, we list the values of s_X in Table S18 for certain values of p_1 and p_2 on the boundaries of the unit square $[0, 1]^2$.

p_i	$\boldsymbol{p}$	$s_X(p_1, p_2)$
$p_1 = p_2 = 0$	ALLD	$\frac{bq_4}{1-q_2+q_4}$
$p_1 = 1,\ 0 \le p_2 < 1$	$(1, p_2, 1, p_2)$	$b - c$
$0 \le p_1 < 1,\ p_2 = 1$	$(p_1, 1, p_1, 1)$	$-c$

Table S18: Examples of s_X. Here, player X uses a reactive strategy $\boldsymbol{p}$ while player Y uses an unbending strategy $\boldsymbol{q}$ from Class A.

13.1 Maximum value of s_X

We claim that the maximum value of s_X is either $s_X(1, p_2)$ ($p_2 \ne 1$) or $s_X(0, 0)$. The proof is straightforward as Δ decreases with respect to both p_1 and p_2. That is,

$$\max \Delta(p_1, p_2) = \Delta(0, 0) = cq_4 - (b - c)(1 - q_2). \tag{96}$$

Further, if $\Delta(0, 0) \le 0$, $\max s_X(p_1, p_2) = s_X(1, p_2)$; otherwise, $\max s_X(p_1, p_2) = s_X(0, 0)$. Define

$$h_{Aa}(q_2) = \frac{(b - c)(1 - q_2)}{c}. \tag{97}$$

We have

$$\max s_X(p_1, p_2) = \begin{cases} s_X(1, p_2), & q_4 \le h_{Aa} \\ s_X(0, 0). & q_4 > h_{Aa} \end{cases} \tag{98}$$

Compare $h_A(q_2)$ and $h_{Aa}(q_2)$. It is straightforward to obtain the solutions to $h_A(q_2) = h_{Aa}(q_2)$, which are $q_2 = 1$ and $q_2 = b(b - c)/c^2$. Notice that

$$b(b - c)/c^2 - q_a = \frac{(b + c)^2(b - c)^2}{c^2(b^2 + bc - c^2)} > 0, \tag{99}$$

and

$$h_{Aa}(q_a) = \frac{b^2(b - c)}{c(b^2 + bc - c^2)} > 0. \tag{100}$$

The relation between $b(b-c)/c^2$ and 1, that is, the relation between r and $(1+\sqrt{5})/2$ (the golden ratio), decides whether s_X always takes the maximum value at $p_1 = 1$ or not. More specifically,

(i) if $1 < r < \frac{1+\sqrt{5}}{2}$,

$$\max s_X(p_1, p_2) = \begin{cases} s_X(1, p_2) = b - c, & 0 < q_4 \leq h_{Aa} \\ s_X(0, 0) = \frac{bq_4}{1-q_2+q_4}, & h_{Aa} < q_4 \leq h_A \end{cases} \tag{101}$$

(ii) if $r \geq \frac{1+\sqrt{5}}{2}$, $\max s_X(p_1, p_2) = s_X(1, p_2) = b - c$.

13.2 Monotonicity of s_X

Consider $s_X(p_1, p_2)$ as a quadratic rational function of p_1 and p_2. We study the monotonicity of s_X with respect to the two variables.

13.2.1 Partial derivative with respect to p_1

We first take the partial derivative with respect to p_1. In particular, we obtain the expressions of $\partial s_X/\partial p_1$ on the boundaries of the unit square $[0, 1]^2$. The results are summarized in Table S19.

p_i	$\boldsymbol{p}$	$\frac{\partial s_X(p_1,p_2)}{\partial p_1}$
	(p_1, p_2, p_3, p_4)	$\frac{q_4(1-p_2)g_{A1}(p_1,p_2)}{f_A^2(p_1,p_2)}$
$p_1 = p_2 = 0$	ALLD	$\frac{\{-[b(1-q_2)+c]q_4+(1-q_2)(bq_2-c)\}q_4}{(1-q_2+q_4)^2}$
$p_1 = 0$ and $p_2 = 1$	$(0, 1, 0, 1)$	0
$p_1 = 0$ and $p_2 = 1-\varepsilon$	$(0, 1-\varepsilon, 0, 1-\varepsilon)$	$\frac{\{-[b(1-q_2)+2b+c]q_4+b-c(1-q_2)\}\varepsilon q_4}{(1-q_2)(1-q_4)^2}$
$p_2 = 0$	$(p_1, 0, p_1, 0)$	$\frac{q_4 g_{A1}(p_1,0)}{f_A^2(p_1,0)}$
$p_1 = 1$	$(1, p_2, 1, p_2)$	$\frac{[b(1-q_2)-c](1-p_2)q_4+(1-q_2)[b-c(1-p_2)]}{[q_2(1-p_2)+p_2](1-p_2)q_4}$

Table S19: Partial derivative of s_X with respect to p_1. The parameter ε is an infinitesimal ($\varepsilon \to 0$).

Here, $f_A(p_1, p_2)$ is defined as before. Besides, $g_{A1}(p_1, p_2) = e_2 p_1^2 + e_1 p_1 + e_0$,

where

$$\begin{cases} e_2 = -(1-q_2)[b(1-q_2)+c]q_4 - c(1-q_2)^2, \\ e_1 = 2(1-q_2)[b(1-q_2)p_2+b+c]q_4 + 2c(1-q_2)^2, \\ e_0 = [q_2(1-p_2)+p_2]\{[b(1-q_2)-c](1-p_2)q_4 + (1-q_2)[b-c(1-p_2)]\} - e_2 - e_1, \end{cases} \tag{102}$$

Moreover, we have $f_A(p_1, 0) = (1-q_2)[(1-p_1) - q_4 p_1^2] + q_4$ and $g_{A1}(p_1, 0) = e_{20}p_1^2 + e_{10}p_1 + e_{00}$, where

$$\begin{cases} e_{20} = e_2, \\ e_{10} = 2(b+c)(1-q_2)q_4 + 2c(1-q_2)^2, \\ e_{10} = q_2\{[b(1-q_2)-c]q_4 + (b-c)(1-q_2)\} - e_2 - e_1. \end{cases} \tag{103}$$

With the expressions in Table S19, we now consider the sign of the partial derivative.

It is straightforward to show that

$$\left.\frac{\partial s_X}{\partial p_1}\right|_{p_1=1} \tag{104}$$

is always positive, which is equivalent to show that the numerator

$$(*) = [b(1-q_2)-c](1-p_2)q_4 + (1-q_2)[b-c(1-p_2)] \tag{105}$$

is positive. As $(*)$ is linear, we only need to consider its values estimated at the boundaries. Letting $p_2 = 0$ and $q_4 = 0$, $p_2 = 0$ and $q_4 = h_A(q_2)$, and $p_2 = 1$, the values are all positive. For example, when $p_2 = 0$ and $q_4 = h_A(q_2)$, $(*)$ becomes

$$\frac{b(b-c)^2(1-q_2)^2[(b+2c)q_2+b-c]}{bc^2q_2^2 - (b-c)(b^2-bc-c^2)q_2 + (b-c)^2(b+c)}, \tag{106}$$

which is greater than zero.

Given that $e_2 < 0$ and $2e_2 + e_1 = 2b(1-q_2)[q_2(1-p_2)+p_2]q_4 > 0$, the curve of g_{A1} (as a function of p_1) is a parabola opening downwards and the axis of symmetry is on the right of $p_1 = 1$. From the above analysis, we also know that when $p_1 = 1$, $g_{A1} > 0$. Therefore, as p_1 increases (p_2 being fixed),

$$\frac{\partial s_X}{\partial p_1} \tag{107}$$

is either always nonnegative or first negative and then positive.

As to

$$\left.\frac{\partial s_X(p_1,p_2)}{\partial p_1}\right|_{(0,0)}, \tag{108}$$

it is easy to see that the derivative is nonnegative if

$$0 < q_4 \le h_a(q_2) = \frac{(bq_2-c)(1-q_2)}{b(1-q_2)+c}. \tag{109}$$

Otherwise, the derivative is negative. Notice that the difference between $h_A(q_2)$ and $h_a(q_2)$ is

$$\frac{bcq_2(1-q_2)^2[(2b+c)(b-c)+bcq_2]}{[b(1-q_2)+c][bc^2q_2^2 - (b-c)(b^2-bc-c^2)q_2 + (b-c)^2(b+c)]}, \tag{110}$$

which is always positive. For the extreme case where the benefit-to-cost ratio $r \to +\infty$, the difference approaches zero. Moreover, the difference between $h_{Aa}(q_2)$ and $h_a(q_2)$ is

$$\frac{b^2(1-q_2)^2}{c[b(1-q_2)+c]}, \tag{111}$$

being positive as well.

Similarly, for

$$\left.\frac{\partial s_X(p_1,p_2)}{\partial p_1}\right|_{(0,1-\varepsilon)}, \varepsilon \to 0 \tag{112}$$

the derivative is nonnegative if

$$0 < q_4 \le \frac{b-c(1-q_2)}{b(1-q_2)+2b+c}. \tag{113}$$

Otherwise, the derivative is negative. Nevertheless, the difference between the right-hand side and $h_A(q_2)$ can be either positive or negative, depending on the values of r and q_2. The explicit expressions are tedious and we only list a few examples here:

(i) $b/c = 1.2$, the difference is always positive,

(ii) $b/c = 2$, the difference is always nonnegative and is zero when $q_2 = 1/2$,

(iii) $b/c = 10$, the difference is nonnegative if $9/109 = q_a < q_2 \le 3/7$ or $9/10 \le q_2 < 1$.

Finally, we consider the sign of

$$\left.\frac{\partial s_X}{\partial p_1}\right|_{p_2=0}. \tag{114}$$

Since $e_{20} = e_2 < 0$ and $2e_{20}+e_{10} = 2bq_2(1-q_2)q_4 > 0$, the curve of $g_{A1}(p_1,0)$ (as a function of p_1) is a parabola opening downwards and the axis of symmetry is on the right of $p_1 = 1$. Also, we have $g_{A1}(1,0) > 0$. Therefore, as p_1 increases, the derivative is either always nonnegative or first negative and then positive.

Combined with previous results, we claim that

(i) $0 < q_4 \le h_a(q_2)$, $\left.\frac{\partial s_X}{\partial p_1}\right|_{p_2=0}$ is nonnegative for $0 \le p_1 \le 1$,

(ii) $h_a(q_2) < q_4 < h_A(q_2)$, $\left.\frac{\partial s_X}{\partial p_1}\right|_{p_2=0}$ is first negative and then positive for $0 \le p_1 \le 1$.

- e.g. $q_2 = (b-c)/b$ and $q_4 = (2b-3c)/4b$ ($h_a(q_2) = (b-2c)/2b$ and $h_A(q_2) = (b-c)/2b$): the partial derivative is negative for $0 \le p_1 < c/(2b-c)$, zero for $p_1 = c/(2b-c)$, and positive for $c/(2b-c) < p_1 \le 1$.

In particular, we can show that $\left.\frac{\partial s_X}{\partial p_1}\right|_{p_2=0}$ is always positive for $p_1 = c/b$ (hence positive for $c/b \le p_1 \le 1$). It is straightforward to show as

$$\left.\frac{\partial s_X}{\partial p_1}\right|_{(c/b,0)} = \frac{[bc^2q_2^2-(b-c)(b^2-bc-c^2)q_2+(b-c)^2(b+c)]b^2q_4(h_A-q_4)}{[(c^2q_2+b^2-c^2)q_4+b(b-c)(1-q_2)]^2}. \tag{115}$$

Therefore, if player X uses an extortionate ZD strategy (recall Figure S19), s_X is free of p_2, and its derivative with respect to p_1 is always positive.

We summarize the above analysis for $\partial s_X/\partial p_1$ in Table S20.

Partial derivative	Sign	Monotonicity
$\frac{\partial s_X}{\partial p_1}$	$+$ or<br>$- \to +$	$\nearrow$ or<br>$\searrow\to\nearrow$
$\frac{\partial s_X(p_1,p_2)}{\partial p_1}\Big\vert_{(0,0)}$	$\begin{cases} +, & 0 < q_4 \leq h_a(q_2) \\ -. & h_a(q_2) < q_4 < h_A(q_2) \end{cases}$	$\begin{cases} \nearrow, & 0 < q_4 \leq h_a(q_2) \\ \searrow. & h_a(q_2) < q_4 < h_A(q_2) \end{cases}$
$\frac{\partial s_X(p_1,p_2)}{\partial p_1}\Big\vert_{(0,1)}$	0	$\to$
$\frac{\partial s_X}{\partial p_1}\Big\vert_{p_2=0}$	$\begin{cases} +, & 0 < q_4 \leq h_a(q_2) \\ - \to +. & h_a(q_2) < q_4 < h_A(q_2) \end{cases}$	$\begin{cases} \nearrow, & 0 < q_4 \leq h_a(q_2) \\ \searrow\to\nearrow. & h_a(q_2) < q_4 < h_A(q_2) \end{cases}$
$\frac{\partial s_X}{\partial p_1}\Big\vert_{p_1=1}$	$+$	$\nearrow$

Table S20: Monotonicity of s_X with respect to p_1 as p_1 increases from 0 to 1. When the sign is $+$, it also includes zero.

13.2.2 Partial derivative with respect to p_2

We then take the partial derivative of s_X with respect to p_2. As before, we consider the expressions of $\partial s_X/\partial p_2$ on the boundaries of the unit square $[0,1]^2$. The results are summarized in Table S21.

p_i	$\boldsymbol{p}$	$\frac{\partial s_X(p_1,p_2)}{\partial p_2}$
$p_1 = 0$	$(0, p_2, 0, p_2)$	$\frac{(1-q_2)[(1-q_4p_2)\delta(p_2)-(1-q_2+q_4)(1-q_4)(cp_2+b)q_4p_2]}{f_A^2(0,p_2)}$
$p_2 = 0$	$(p_1, 0, p_1, 0)$	$\frac{(1-q_2)(1-p_1)g_{A2}(p_1,0)}{f_A^2(p_1,0)}$
$p_1 = 1$	$(1, p_2, 1, p_2)$	0
$p_1 = 1-\varepsilon$ and		
$p_2 = 0$	$(1-\varepsilon, 0, 1-\varepsilon, 0)$	$\frac{\epsilon(1-q_2)[(b-c-bq_2)q_4-(2b-c)q_2+(b-c)]}{q_2^2q_4}$
$p_2 = 1-c/b$	$(1-\varepsilon, 1-c/b, 1-\varepsilon, 1-c/b)$	$\frac{b^2\epsilon(1-q_2)(*)}{c^2(cq_2+b-c)^2q_4}$
$p_2 = 1$	$(1-\varepsilon, 1, 1-\varepsilon, 1)$	$-\frac{bq_4}{\epsilon(1-q_2)}$

Table S21: Partial derivative of s_X with respect to p_2. The parameter ε is an infinitesimal ($\varepsilon \to 0$).

Here, f_A is defined as before. Besides,

$$\delta(p_2) = [(b-c)(1-q_2) - cq_4]q_4(1-p_2) - (1-q_4)[c(1-q_2) + (b+c)q_4]. \quad (116)$$

Moreover, we have $g_{A2}(p_1, 0) = e_{20}p_1^2 + e_{10}p_1 + e_{00}$, where

$$\begin{cases} e_{20} = [b(1-q_2) + c]q_4^2 + c(1-q_2)q_4, \\ e_{10} = [(b-c-bq_2)q_4 - (2b-c)q_2 + (b-c)]q_4 - e_{20} - e_{00}, \\ e_{00} = bq_4^2 - (bq_2 + c)q_4 - c(1-q_2). \end{cases} \quad (117)$$

In addition,

$$(*) = -c(bcq_4 + 2b^2 - c^2)q_2 + (b-c)(c^2q_4 - b^2 + bc + c^2). \quad (118)$$

With the expressions in Table S21, we now consider the sign of the partial derivative.

We first show that

$$\left.\frac{\partial s_X(p_1,p_2)}{\partial p_2}\right|_{p_1=0} \quad (119)$$

is always negative. It can be told that

$$\delta(p_2) < 0 \Rightarrow \left.\frac{\partial s_X(p_1,p_2)}{\partial p_2}\right|_{p_1=0} < 0. \quad (120)$$

As $\delta(p_2)$ is a linear function of p_2, to show that $\delta(p_2) < 0$, we only need to show that $\delta(0) < 0$ and $\delta(1) < 0$. The latter is $-(1-q_4)[c(1-q_2) + (b+c)q_4]$ and the former is

$$\delta(0) = bq_4^2 - (bq_2 + c)q_4 - c(1-q_2). \quad (121)$$

Treat it as a quadratic function of q_4, corresponding to a parabola opening upwards. Its value at $q_4 = 0$ and $q_4 = q_2 + c/b$ are both negative. Therefore, to show that $\delta(0) < 0$ always holds for $0 < q_4 \leq h_A(q_2)$, it suffices to show that $h_A(q_2) \leq q_2 + c/b$. The proof is tedious but trivial. In particular, if the benefit-to-cost ratio $r \to \infty$, we get $c/b \to 0$ and $h_A(q_2) \to q_2$.

We then show that

$$\left.\frac{\partial s_X(p_1,p_2)}{\partial p_2}\right|_{p_2=0} \tag{122}$$

is either always nonpositive or first negative and then positive as p_1 increases. Note that $g_{A2}(p_1, 0)$ is a quadratic function of p_1, corresponding to a parabola opening upwards. Also, from the above analysis, we know that $g_{A2}(0, 0) < 0$. Therefore, as p_1 increases, if $g_{A2}(1, 0) \leq 0$, the derivative is always nonpositive; otherwise, it is first negative and then positive.

We have

$$g_{A2}(1,0) = [(b - c - bq_2)q_4 - (2b - c)q_2 + (b - c)]q_4. \tag{123}$$

That is, $g_{A2}(1, 0) \leq 0$ if

$$\begin{cases} \frac{b-c}{2b-c} < q_2 < \frac{b-c}{b}, \\ 0 < q_4 \leq \min\{h_A(q_2), \frac{(2b-c)q_2-(b-c)}{b-c-bq_2}\}, \end{cases} \quad \text{or} \quad \begin{cases} \frac{b-c}{b} \leq q_2 < 1, \\ 0 < q_4 \leq h_A(q_2). \end{cases} \tag{124}$$

Otherwise, $g_{A2}(1, 0) > 0$.

Finally, given different values of p_2, we consider the sign of

$$\left.\frac{\partial s_X(p_1,p_2)}{\partial p_2}\right|_{p_1=1-\epsilon}, \epsilon \to 0. \tag{125}$$

If $p_2 = 0$, we draw the same conclusion as Equation 124. If $p_2 = 1$, the derivative is negative. Further, if $p_2 = 1 - c/b$ (where $\boldsymbol{p}$ approaches GTFT), we show that the derivative is also negative. It is equivalent to show that $(*) < 0$. Consider $(*)$ as a linear function of q_2. We have

$$(*)(q_a) = -\frac{(b+c)(b-c)^2(b^2-c^2q_4)}{b^2+bc-c^2}, \qquad (*)(1) = -b^3 - c^3q_4. \tag{126}$$

Therefore, $(*) < 0$ always holds for $q_a < q_2 < 1$. Hence the derivative is negative.

We summarize the above analysis for $\partial s_X/\partial p_2$ in Table S22.

Partial derivative	Sign	Monotonicity
$\left.\frac{\partial s_X(p_1,p_2)}{\partial p_2}\right\vert_{p_1=0}$	$-$	$\searrow$
$\left.\frac{\partial s_X(p_1,p_2)}{\partial p_2}\right\vert_{p_2=0}$	$-$ or<br>$- \to +$	$\searrow$<br>or $\searrow \to \nearrow$
$\left.\frac{\partial s_X(p_1,p_2)}{\partial p_2}\right\vert_{p_1=1}$	0	$\to$
$\left.\frac{\partial s_X(p_1,p_2)}{\partial p_2}\right\vert_{(1-\epsilon,0)}$	$-$ or $+$	$\searrow$ or $\nearrow$
$\left.\frac{\partial s_X(p_1,p_2)}{\partial p_2}\right\vert_{(1-\epsilon,1-c/b)}$	$-$	$\searrow$
$\left.\frac{\partial s_X(p_1,p_2)}{\partial p_2}\right\vert_{(1-\epsilon,1)}$	$-$	$\searrow$

Table S22: Monotonicity of s_X with respect to p_2 as p_1 increases from 0 to 1. When the sign is $-$, it also includes zero. As before, the parameter ε is an infinitesimal ($\varepsilon \to 0$).

13.3 Conclusion and example

To conclude, we emphasize how the benefit-to-cost ratio r would shape the aforementioned regions of

(i) $s_X \leq b - c$, that is, $\max s_X(p_1, p_2) = s_X(1, p_2) = b - c$,

(ii) unbending strategies, and

(iii) $\partial s_X(0, 0)/\partial p_1 \geq 0$, or equivalently, $\partial s_X(p_1, 0)/\partial p_1 \geq 0$.

Mathematically, the three regions correspond to

(i)

$$q_4 \leq h_{Aa}(q_2) = \frac{(b-c)(1-q_2)}{c}, \tag{127}$$

(ii)

$$q_4 \leq h_A(q_2) = \frac{(b-c)(1-q_2)[(b^2+bc-c^2)q_2-(b-c)c]}{bc^2q_2^2-(b-c)(b^2-bc-c^2)q_2+(b-c)^2(b+c)}, \tag{128}$$

and

(iii)

$$q_4 \leq h_a(q_2) = \frac{(bq_2-c)(1-q_2)}{b(1-q_2)+c}. \tag{129}$$

It is worth pointing out that $h_a \leq h_{Aa}$ and $h_a \leq h_A$ always hold. Also,

$$\left.\frac{dh_A}{dq_2}\right|_{q_2=1} = \left.\frac{dh_{Aa}}{dq_2}\right|_{q_2=1} = \left.\frac{dh_a}{dq_2}\right|_{q_2=1} = -\frac{b-c}{c} = -(r-1). \tag{130}$$

The visualization is given in Figure S20.

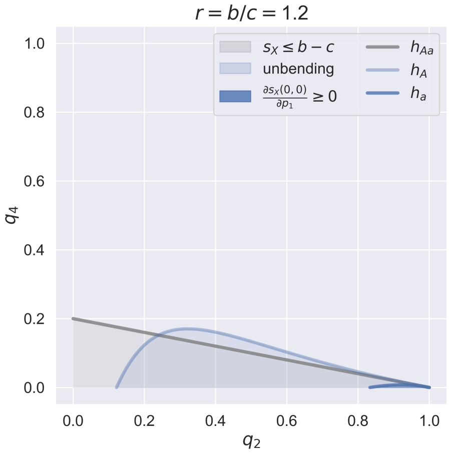

(a) $r = 1.2$

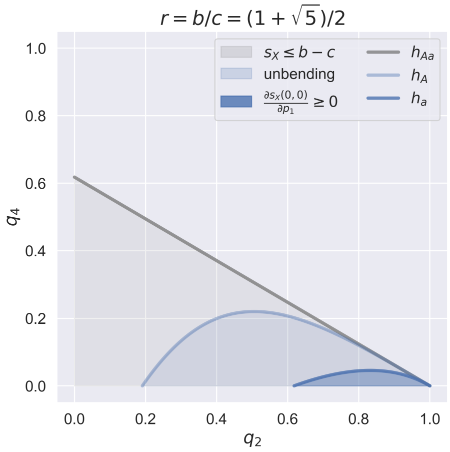

(b) $r = (1 + \sqrt{5})/2$

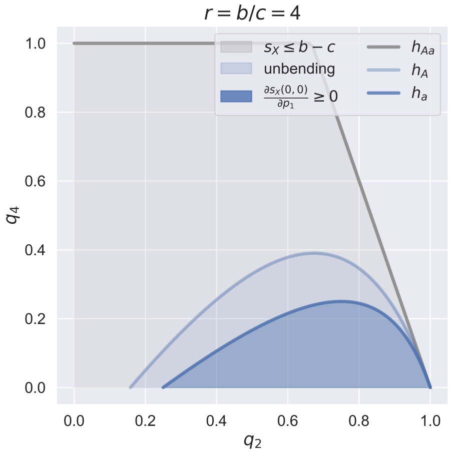

(c) $r = 4$

Figure S20: Regions of $0 < h_4 \leq h_{Aa}$, $0 < h_4 \leq h_A$, and $0 < h_4 \leq h_a$ for the donation game with increasing benefit-to-cost ratios.

Based on the conclusion, we show a few examples of the payoff s_X and its partial derivatives with respect to p_1 and p_2 in Table S23.

<table>
<tr><th></th><th></th><th>$\partial s_X(0,0)/\partial p_1 < 0$</th><th>$\partial s_X(0,0)/\partial p_1 > 0$</th></tr>
<tr><td rowspan="2">$r < r^*$</td><td>$\max s_X = s_X(0,0)$</td><td>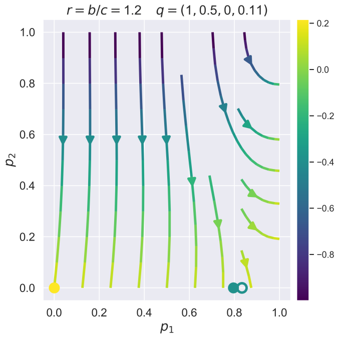
</td><td></td></tr>
<tr><td>$\max s_X = s_X(1,p_1)$</td><td>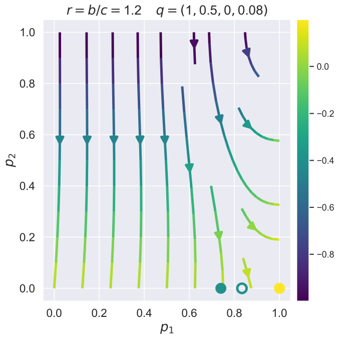
</td><td>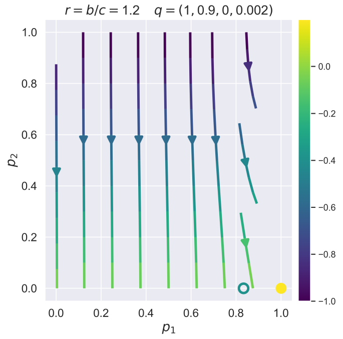
</td></tr>
<tr><td colspan="2">$r > r^*$
$\max s_X = s_X(1,p_1)$</td><td>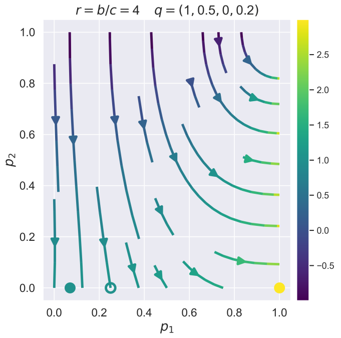
</td><td>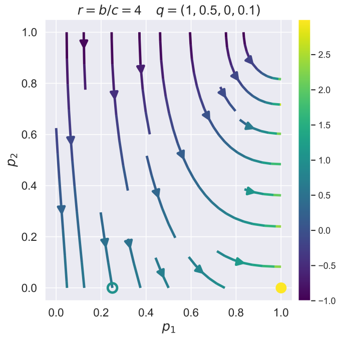
</td></tr>
</table>

Table S23: Stream plot of s_X. Here, $r^* = (1+\sqrt{5})/2$ is the golden ratio. The 2-dimensional gradient vector field of s_X is given with respect to p_1 and p_2. The color at a point (p_1, p_2) reflects the value of s_X at that point (with reference to the color bar). The yellow point denotes where the maximum value of s_X lies (if there is more than one point, only the bottom one with $p_2 = 0$ is shown). The solid green point indicates where $\partial s_X(p_1, 0)/\partial p_1$ becomes zero and the empty green point is $(c/b, 0)$ (the left boundary of extortionate ZD strategies).

14 Under influence of class D of unbending strategies (continued)

Let $\boldsymbol{p} = (p_1, p_2, p_1, p_2)$ (a reactive strategy) and $\boldsymbol{q} = (q_1, q_2, q_3, q_4)$ (an unbending strategy from Class D), where q_i's satisfy

$$q_4 = h_D(q_1, q_2, q_3) = q_2 + q_3 - q_1, \tag{131}$$

and

$$b(q_2 - q_1) + c(q_3 - q_1) + c = d_{D0} < 0 < a_{D0} = (b - c)(q_2 + q_3 - q_1). \tag{132}$$

Notice that $q_3 = 0$ is impossible.

We obtain $s_X(p_1, p_2)$ as a linear rational function of p_1 and p_2. In particular, when $p_1 = p_2 = 1$, we have

$$s_X(1,1) = \frac{(b - c)q_3 - c(1 - q_1)}{1 - q_1 + q_3}. \tag{133}$$

On the other hand, when $p_1 = q_1 = 1$, $s_X = R = b - c$. Moreover, we get

$$\begin{aligned} s_X(p_1, p_2) - s_X(1,1) &= \frac{[b(q_2 - q_1) + c(q_3 - q_1) + c][q_3(1 - p_1) + (1 - q_1)(1 - p_2)]}{(1 - q_1 + q_3)[1 - q_1 + q_3 + (q_1 - q_2)(p_2 - p_1)]} \\ &= \frac{d_{D0}[q_3(1 - p_1) + (1 - q_1)(1 - p_2)]}{(1 - q_1 + q_3)[1 - q_1 + q_3 + (q_1 - q_2)(p_2 - p_1)]}. \end{aligned} \tag{134}$$

14.1 Maximum value of s_X

We claim that the maximum value of s_X is $s_X(1,1)$ if $q_1 \neq 1$ ($\boldsymbol{q}$ is not a generous ZD strategy) and $s_X(1, p_2)$ if $q_1 = 1$ ($\boldsymbol{q}$ is a generous ZD strategy).

Given that $a_{D0} > 0$, $q_1 < q_2 + q_3$, we have

$$1 - q_1 + q_3 + (q_1 - q_2)(p_2 - p_1) > 1 - q_2 + (q_1 - q_2)(p_2 - p_1) \geq 0. \tag{135}$$

Therefore, the denominator of $s_X(p_1, p_2) - s_X(1,1)$ is always positive. Meanwhile, we know that $d_{D0} < 0$. Besides,

$$q_3(1 - p_1) + (1 - q_1)(1 - p_2) \geq 0, \tag{136}$$

where the equality holds if $p_1 = p_2 = 1$ or $p_1 = q_1 = 1$. That is, $s_X(p_1, p_2) - s_X(1,1) \leq 0$, where the equality holds if $p_1 = p_2 = 1$ or $p_1 = q_1 = 1$.

14.2 Monotonicity of s_X

Treat $s_X(p_1, p_2)$ as a linear rational function of p_1 and p_2. We study the monotonicity of s_X with respect to the two variables.

14.2.1 Partial derivative with respect to p_1

We first take the partial derivative with respect to p_1:

$$\begin{aligned}\frac{\partial s_X(p_1,p_2)}{\partial p_1} &= -\frac{[b(q_2-q_1)+c(q_3-q_1)+c][(q_1-q_2)p_2+q_2+q_3-q_1]}{[1-q_1+q_3+(q_1-q_2)(p_2-p_1)]^2}\\ &= -\frac{d_{D0}[(q_1-q_2)p_2+q_2+q_3-q_1]}{[1-q_1+q_3+(q_1-q_2)(p_2-p_1)]^2} > 0.\end{aligned} \tag{137}$$

Therefore, s_X always increases with respect to p_1 for $0 \le p_1 \le 1$.

14.2.2 Partial derivative with respect to p_2

We then take the partial derivative with respect to p_2:

$$\begin{aligned}\frac{\partial s_X(p_1,p_2)}{\partial p_2} &= -\frac{[b(q_2-q_1)+c(q_3-q_1)+c][(q_2-q_1)p_1+1-q_2]}{[1-q_1+q_3+(q_1-q_2)(p_2-p_1)]^2}\\ &= -\frac{d_{D0}[(q_2-q_1)p_1+1-q_2]}{[1-q_1+q_3+(q_1-q_2)(p_2-p_1)]^2} \ge 0.\end{aligned} \tag{138}$$

The equality holds when $p_1 = q_1 = 1$. Therefore, if $p_1 \neq 1$ or $q_1 \neq 1$, s_X always increases with respect to p_2; otherwise, $s_X = b - c$, being a constant.

14.3 Conclusion and example

To conclude, we show how the type of general ZD strategies would determine the maximum value and the monotonicity of s_X.

(i) If $q_1 \neq 1$, that is, if $\boldsymbol{q}$ is not a generous ZD strategy,

$$\max s_X(p_1,p_2) = s_X(1,1) = \frac{(b-c)q_3 - c(1-q_1)}{1-q_1+q_3}, \tag{139}$$

and

$$\frac{\partial s_X(p_1,p_2)}{\partial p_1} > 0, \frac{\partial s_X(p_1,p_2)}{\partial p_2} > 0. \tag{140}$$

(ii) If $q_1 = 1$, that is, if $\boldsymbol{q}$ is a generous ZD strategy,

$$\max s_X(p_1,p_2) = s_X(1,p_2) = b - c, \tag{141}$$

and

$$\frac{\partial s_X(p_1,p_2)}{\partial p_1} > 0, \frac{\partial s_X(p_1,p_2)}{\partial p_2} \begin{cases} > 0, & p_1 \neq 1 \\ = 0. & p_1 = 1 \end{cases} \tag{142}$$

We also give two examples of the payoff s_X and its partial derivatives with respect to p_1 and p_2 in Table S24.

<table>
<tr>
<td>$\max s_X(p_1, p_2) = s_X(1, 1)$
$\left.\frac{\partial s_X(p_1,p_2)}{\partial p_2}\right|_{p_1=1} > 0$</td>
<td>$\max s_X(p_1, p_2) = s_X(1, p_2)$
$\left.\frac{\partial s_X(p_1,p_2)}{\partial p_2}\right|_{p_1=1} = 0$</td>
</tr>
<tr>
<td>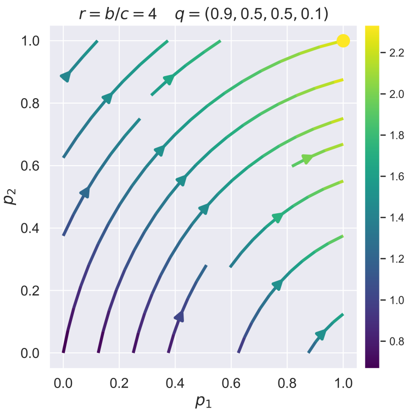
</td>
<td>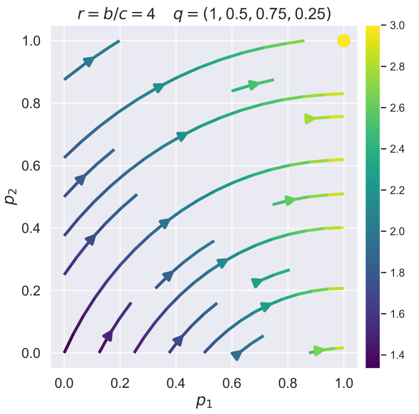
</td>
</tr>
</table>

Table S24: Stream plot of s_X. The 2-dimensional gradient vector field of s_X is given with respect to p_1 and p_2. The color at a point (p_1, p_2) reflects the value of s_X at that point (with reference to the color bar). The yellow point denotes where the maximum value of s_X lies (if there is more than one point, only the top one with $p_2 = 1$ is shown).

15 Steering learning dynamics from extortion to fairness and cooperation: general ZD strategies

Recall Figure S19. Reactive strategies is a subset of general ZD strategies in donation games. In particular, we can consider the set of reactive ZD strategies $\boldsymbol{p} = (p_1, p_2, p_1, p_2)$, where p_1 and p_2 can be written as functions of O and χ (see Equation 90). When playing against a fixed unbending co-player, this parametrization in terms of O and χ actually includes all possible variations of general ZD strategies that can have a distinct effect on the average payoff s_X since the parameter ϕ is already neutralized by unbending strategies in the first place.

We have studied the most general case where player X uses a reactive strategy (subset of ZD) and player Y uses an unbending strategy in the donation game. We now extend it to general ZD strategies parameterized by (O, χ) for player X as the following.

15.1 Under influence of class A of unbending strategies (continued)

Let $\boldsymbol{q}$ be an unbending strategy from Class A. We obtain $s_X(O, \chi)$ as a quadratic rational function of O and χ. Its monotonicity with respect to the two variables can be further discussed. Here, we summarize the corresponding analysis on the boundaries.

- $\left.\frac{\partial s_X(O,\chi)}{\partial O}\right|_{\chi=1} = 0.$
- $\left.\frac{\partial s_X(O,\chi)}{\partial \chi}\right|_{O=b-c} = 0.$
- $$\left.\frac{\partial s_X(O,\chi)}{\partial \chi}\right|_{\chi=1} = \frac{(b-c-O)(*)}{(b+c)q_2q_4} < 0. \tag{143}$$
  Here, $(*) = -[(b-c)(1-q_2+q_4) - bq_2q_4] < 0$ for $0 < q_4 \leq h_A$. The proof is straightforward. In particular, its value at $q_4 = h_A$ is
  $$-\frac{b(b-c)^2[(b+2c)q_2+b-c](1-q_2)^2}{bc^2q_2^2-(b-c)(b^2-bc-c^2)q_2+(b-c)^2(b+c)} > 0. \tag{144}$$

Two examples of the payoff s_X and its partial derivatives are given in Table S25.

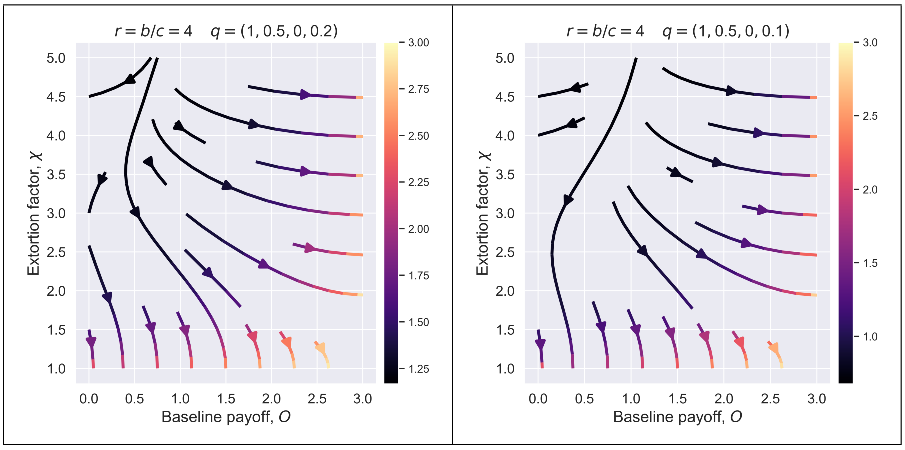

Table S25: Stream plot of s_X. The 2-dimensional gradient vector field of s_X is given with respect to O and χ. The color at a point (O, χ) reflects the value of s_X at that point (with reference to the color bar).

15.2 Under influence of class D of unbending strategies (continued)

Let $\boldsymbol{q}$ be an unbending strategy from Class D. Still, $s_X(O, \chi)$ can be treated a quadratic rational function of O and χ. Its monotonicity with respect to the two variables can be further discussed.

15.2.1 Partial derivative with respect to O

We first take the partial derivative with respect to the baseline payoff O. We get

$$\frac{\partial s_X}{\partial O} = -\frac{(*)}{[b(q_2-q_1)+c(q_3-q_1)+c](\chi-1)} = -\frac{(*)}{d_{D0}(\chi-1)}. \tag{145}$$

Here,

$$\begin{aligned}(*) &= [c(q_2-q_1)+b(q_3-q_1)+b]\chi + b(q_2-q_1)+c(q_3-q_1)+c\\ &= [c(q_2-q_1)+b(q_3-q_1)+b](\chi-1)+(b-c)(q_2+q_3-q_1+1-q_1)\\ &= [(b+c)(h_D+1-q_1)-d_{D0}](\chi-1)+(b-c)(h_D+1-q_1)\\ &> 0.\end{aligned} \tag{146}$$

Therefore, $\partial s_X/\partial O \geq 0$ and the equality holds if $\chi = 1$.

We then take the partial derivative with respect to the extortion factor χ. We obtain

$$\frac{\partial s_X}{\partial \chi} = -\frac{(b+c)[b(q_2-q_1)+c(q_3-q_1)+c](**)}{(*)^2} = -\frac{(b+c)d_{D0}(**)}{(*)^2}. \tag{147}$$

here, $(*)$ is the same as defined above and

$$(**) = (q_2+q_3-q_1+1-q_1)O-(b-c)(q_2+q_3-q_1). \tag{148}$$

Notice that Class D is actually the set of general ZD strategies with baseline payoff O' satisfying $0 < O' \leq b-c$ and that

$$O' = \frac{(b-c)(q_2+q_3-q_1)}{q_2+q_3-q_1+1-q_1} \tag{149}$$

is the solution of $(**)$. Therefore, we get

$$\frac{\partial s_X}{\partial \chi}\begin{cases} <0, & O<O'\\ =0, & O=O'\\ >0. & O>O'\end{cases} \tag{150}$$

For instance, we always have

$$\left.\frac{\partial s_X(O,\chi)}{\partial \chi}\right|_{O=0} < 0 \quad \text{and} \quad \left.\frac{\partial s_X(O,\chi)}{\partial \chi}\right|_{O=b-c} \geq 0. \tag{151}$$

The equality holds if $O' = b-c$, or equivalently, $q_1 = 1$ (player Y uses a generous ZD strategy).

Two examples of the payoff s_X and its partial derivatives are given in Table S26.

$\left.\frac{\partial s_X(O,\chi)}{\partial \chi}\right\|_{O=b-c} > 0$	$\left.\frac{\partial s_X(O,\chi)}{\partial \chi}\right\|_{O=b-c} = 0$
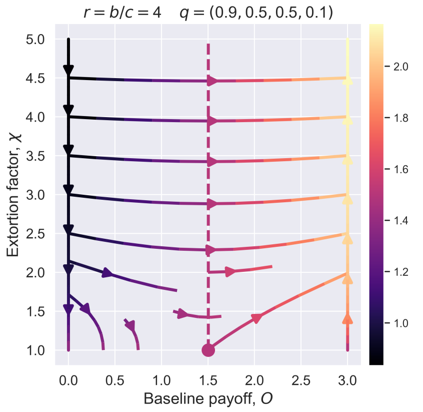	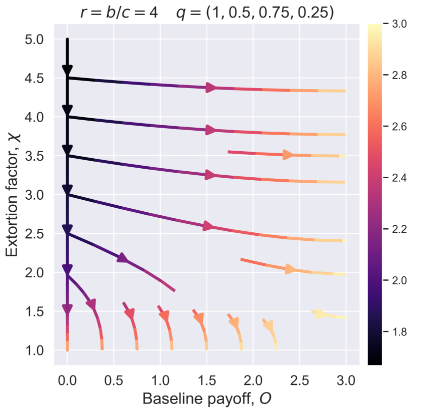

Table S26: Stream plot of s_X. The 2-dimensional gradient vector field of s_X is given with respect to O and χ. The color at a point (O, χ) reflects the value of s_X at that point (with reference to the color bar). The dashed line in the first panel indicates where $\partial s_X/\partial \chi$ turns from negative to positive, that is, where the two baseline payoffs O and O' are equal.

16 Extended search of unbending strategies against general ZD co-players

Finally, we consider that player X uses a more general ZD strategy with the baseline payoff O satisfies $P < O < R$. For a given O, the sets of admissible unbending strategies from the four classes discussed previously need to be updated based on the original definition of unbending properties.

As before, if the opponent Y tries a common strategy such as ALLC, the payoff of X will be $(T - S)(R - O)\chi/[(R - S)\chi + T - R] + O$, which does not involve ϕ. However, when X plays against a more general opponent, its payoff is oftentimes dependent on ϕ. Moreover, it can be shown that when χ is fixed, s_X is a monotonic function of ϕ (the expression of s_X takes the form of a linear rational function of ϕ).

Similarly, a necessary condition $s_X(\boldsymbol{q}, \chi, \phi^{\text{upper}}) = s_X(\boldsymbol{q}, \chi, \phi^{\text{upper}}/2)$ yields the solutions of $\boldsymbol{q}$ for s_X being independent of ϕ (see Table S27, which shares a few solutions with, but is not identical to Table S1).

Solution	s_X
$q_1 = q_2 = 1$	$\frac{(T-S)(R-O)\chi}{(R-S)\chi+(T-R)} + O$
$q_2 = 1$ and $q_4 = 0$	$\frac{(T-S)(O-P)(a_{O1}\chi+a_{O0})\chi}{f_O(\chi)} + O$
$q_3 = q_4 = 0$	$-\frac{(T-S)(O-P)\chi}{(T-P)\chi+(P-S)} + O$
$q_1 = 1$ and $q_3 = 0$	$\frac{(T-S)(R-O)(a_{A1}\chi+a_{A0})\chi}{f_A(\chi)} + O$
$q_1 = q_2 = q_3 = q_4$	$\frac{(T-S)[(T+S-R-P)q_1^2-(T+S-2P)q_1+O-P]\chi}{f_C(\chi)} + O$
$q_1 = \frac{O-P-(T+S-O-P)q_2}{O-P-(T+S-R-P)q_2}$, $q_2 = q_3$, and $q_4 = \frac{(O-P)(1-q_2)}{(T+S-O-P)-(T+S-R-P)q_2}$	O
$q_4 = h_D$	$\frac{(T-S)[-(T+S-2O)q_1+(R-O)(q_2+q_3)+T+S-R-O]\chi}{f_D(\chi)} + O$

Table S27: Solutions of $\boldsymbol{q}$ for s_X being independent of ϕ and the corresponding expressions of s_X. Here, h_D is the same multivariate linear function of q_1, q_2 and q_3 as before. Additionally, f_O and f_A are quadratic functions of χ, whereas f_C and f_D are linear functions of χ.

It is straightforward to see that if $q_1 = q_2 = 1$, the derivative $ds_X/d\chi = (T-R)(T-S)(R-O)/[(R-S)\chi + (T-R)]^2$ will always be positive. On the other hand, if $q_3 = q_4 = 0$, the derivative $ds_X/d\chi = -(T-S)(P-S)(O-P)/[(T-P)\chi + (P-S)]^2$ will always be negative.

The two solutions $q_2 = 1$ and $q_4 = 0$ and $q_1 = q_2 = q_3 = q_4$ also allow unbending properties.

Assume that $q_2 = 1$ and $q_4 = 0$. If we further let $q_3 = 0$ (a subset of the strategies satisfying $q_3 = q_4 = 0$), s_X will be a decreasing function of χ. If we let $q_3 = 1$ instead, whether s_X can be a decreasing function of χ depends on the relation between $T + S$ and $2O$. To be more specific, if $T + S < 2O$ (that is, $\max\{T + S, 2P\} < 2O$), $ds_X/d\chi$ will be negative for $0 \leq q_1 < (2O - T - S)/(R + O - T - S)$.

On the other hand, assume that $q_1 = q_2 = q_3 = q_4$. If we further let $q_1 = 0$ ($\boldsymbol{q} = (0, 0, 0, 0)$), s_X will be a decreasing function of χ. More generally, $ds_X/d\chi$ will be negative for $0 \leq q_1 < (*)$, where $(*)$ is the root between 0 and 1 of the

equation

$$(T+S-R-P)q_1^2-(T+S-2P)q_1+O-P=0. \tag{152}$$

More interestingly, if $\boldsymbol{q}=(q_1,q_2,q_3,q_4)$ satisfies

$$q_1=q_{1s}=\frac{O-P-(T+S-O-P)q_2}{O-P-(T+S-R-P)q_2}, \tag{153}$$

$$q_2=q_3, \tag{154}$$

and

$$q_4=q_{4s}=\frac{(O-P)(1-q_2)}{(T+S-O-P)-(T+S-R-P)q_2}, \tag{155}$$

we have $s_X=s_Y=O$. That is, as long as player Y is aware of the baseline payoff O used by player X, it can apply such a strategy $\boldsymbol{q}$ that the payoffs of both players will be fixed to O no matter how large the extortion factor χ is. The set of $\boldsymbol{q}$ in different cases (depending on the relation between $T+S$ and $2P$) is given in Figure S21.

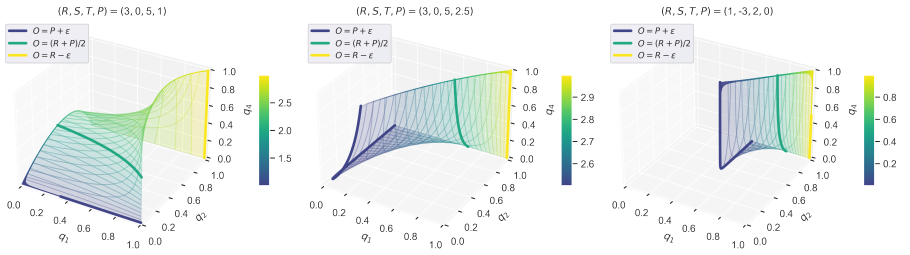

(a) $T+S>2P$ (b) $T+S=2P$ (c) $T+S<2P$

Figure S21: Set of points satisfying $q_1=q_{1s}$ and $q_4=q_{4s}$. The color map indicates the value of O. For a given O, (q_1,q_2,q_4) forms a curve in the 3-dimensional space. The three curves corresponding to $O=P+\varepsilon$, $O=(R+P)/2$ and $O=R-\varepsilon$ are highlighted. Here, $\varepsilon=0.001$.

In particular, if $O=P$, the expressions of $\boldsymbol{q}$ in different cases can be further simplified.

(i) $T+S>2P$: there is no admissible $\boldsymbol{q}$.

(ii) $T+S=2P$: $\boldsymbol{q}=(0,q_2,q_2,0)$, where $0\le q_2\le 1$.

(iii) $T+S<2P$: $\boldsymbol{q}=(\frac{2P-T-S}{R+P-T-S},q_2,q_2,0)$, where $0\le q_2\le 1$.

And if $O=P+\varepsilon$ ($\varepsilon\to 0$), we get the following sets of $\boldsymbol{q}$ in different cases.

(i) $T+S>2P$:

$$\boldsymbol{q}=(\tfrac{\varepsilon-(T+S-2P-\varepsilon)q_2}{\varepsilon-(T+S-R-P)q_2},q_2,q_3,\tfrac{\varepsilon(1-q_2)}{(T+S-2P-\varepsilon)-(T+S-R-P)q_2}), \tag{156}$$

where

$$0\le q_1\le 1, 0\le q_2\le \tfrac{\varepsilon}{T+S-2P-\varepsilon}, \tag{157}$$

and

$$\frac{(T+S-2P-2\varepsilon)\varepsilon}{(T+S-2P-\varepsilon)^2-(T+S-R-P)\varepsilon} \leq q_4 \leq \frac{\varepsilon}{T+S-2P-\varepsilon}. \tag{158}$$

For example, for the conventional IPD game where $(R, S, T, P) = (3, 0, 5, 1)$, we have

$$\boldsymbol{q} = (\frac{\varepsilon-(3-\varepsilon)q_2}{\varepsilon-q_2}, q_2, q_2, \frac{\varepsilon(1-q_2)}{(3-\varepsilon)-q_2}), \tag{159}$$

where

$$0 \leq q_1 \leq 1, 0 \leq q_2 \leq \frac{\varepsilon}{3-\varepsilon}, \text{ and } \frac{(3-2\varepsilon)\varepsilon}{(3-\varepsilon)^2-\varepsilon} \leq q_4 \leq \frac{\varepsilon}{3-\varepsilon}. \tag{160}$$

(ii) $T + S = 2P$:

$$\boldsymbol{q} = (\frac{\varepsilon(1+q_2)}{\varepsilon+(R-P)q_2}, q_2, q_3, \frac{\varepsilon(1-q_2)}{(R-P)q_2-\varepsilon}), \tag{161}$$

where

$$\frac{2\varepsilon}{R-P+\varepsilon} \leq q_1 \leq \frac{R-P+3\varepsilon}{3(R-P)+\varepsilon}, \frac{2\varepsilon}{R-P+\varepsilon} \leq q_2 \leq 1, \text{ and } 0 \leq q_4 \leq 1. \tag{162}$$

(iii) $T + S < 2P$:

$$\boldsymbol{q} = (\frac{\varepsilon+(2P-T-S+\varepsilon)q_2}{\varepsilon+(R+P-T-S)q_2}, q_2, q_3, \frac{\varepsilon(1-q_2)}{(R+P-T-S)q_2-(2P-T-S+\varepsilon)}), \tag{163}$$

where

$$\frac{2P-T-S+2\varepsilon}{R+P-T-S+\varepsilon} \leq q_1 \leq \frac{(2P-T-S+3\varepsilon)(2P-T-S)+(R+P-T-S)\varepsilon+3\varepsilon^2}{(R+P-T-S)(2P-T-S)+3(R+P-T-S)\varepsilon+\varepsilon^2}, \tag{164}$$

$$\frac{2P-T-S+2\varepsilon}{R+P-T-S+\varepsilon} \leq q_2 \leq 1, \text{ and } 0 \leq q_4 \leq 1. \tag{165}$$

Likewise, we can try to figure out the set of unbending strategies based on Table S27. However, for a general O between P and R, it is not always possible to get the explicit expression of $\boldsymbol{q}$. For the rest of the analysis, we will focus on Class A, where $q_1 = 1$ and $q_3 = 0$ and Class D, where $q_4 = h_D$.

17 Class A of unbending strategies (continued)

As before, Class A is under the restriction of $q_1 = 1$ and $q_3 = 0$. The ZD player's payoff s_X is a quadratic rational function of χ (recall Table S27). We have

$$s_X = \frac{(T-S)(R-O)(a_{A1}\chi + a_{A0})\chi}{f_A(\chi)} + O, \tag{166}$$

where

$$\begin{cases} a_{A1} = [(P-S)q_2 + (T+S-O-P)]q_4 - (O-P)(1-q_2), \\ a_{A0} = [(T-P)q_2 - (T+S-O-P)]q_4 + (O-P)(1-q_2). \end{cases} \tag{167}$$

Moreover, the denominator $f_A(\chi) = d_{A2}\chi^2 + d_{A1}\chi + d_{A0}$ is a quadratic function of χ with

$$\begin{cases} d_{A2} = [(T-R)(P-S)q_2 + (T-S)(R-P) - (R-S)(O-P)]q_4 + (T-P)(R-O)(1-q_2), \\ d_{A1} = \{[T(T-P) - S(P-S) - R(T+S-2P)]q_2 + (2R-T-S)(O-P)\}q_4 - (T+S-2P)(R-O)(1-q_2), \\ d_{A0} = [(T-P)(R-S)q_2 + (O-P)(T-R) - (T-S)(R-P)]q_4 - (R-O)(P-S)(1-q_2). \end{cases} \tag{168}$$

If $q_2 = 0$, we have

$$\begin{aligned} s_X &= \frac{a_{A0}\chi}{d_{A1}\chi + d_{A0}} + O, \\ \frac{ds_X}{d\chi} &= \frac{a_{A0}d_{A0}}{(d_{A1}\chi + d_{A0})^2}, \end{aligned} \tag{169}$$

where

$$\begin{cases} a_{A0} = (T-S)(R-O)[(T+S-O-P)q_4 - (O-P)], \\ d_{A1} = [(T-S)(R-O) + (T-R)(O-P)]q_4 + (T-P)(R-O), \\ d_{A0} = [(T-S)(R-O) + (R-S)(O-P)]q_4 + (R-O)(P-S). \end{cases} \tag{170}$$

The set of unbending strategies depends on the relation between $T + S$ and $2P$. We present closed-form analysis in two different cases: $T + S = 2P$ and $T + S < 2P$, whereas for $T + S > 2P$ (see Fig. 3a in the main text) we resort to numerical solutions to find unbending strategies.

17.1 Case II: $T + S = 2P$

If $T + S = 2P$, we have

$$s_X = \tfrac{(T-S)(R-O)(a_{A1}\chi + a_{A0})\chi}{f_A(\chi)} + O, \tag{171}$$

where

$$\begin{cases} a_{A1} = [(T-S)q_2 - (2O-T-S)]q_4 - (2O-T-S)(1-q_2), \\ a_{A0} = [(T-S)q_2 + (2O-T-S)]q_4 + (2O-T-S)(1-q_2), \end{cases} \tag{172}$$

and $f_A(\chi) = d_{A2}\chi^2 + d_{A1}\chi + d_{A0}$ with

$$\begin{cases} d_{A2} = [(T-R)(T-S)q_2 + (T-S)(2R-T-S) - (R-S)(2O-T-S)]q_4 + (T-S)(R-O)(1-q_2), \\ d_{A1} = [(T-S)^2 q_2 + (2R-T-S)(2O-T-S)]q_4, \\ d_{A0} = [(T-S)(R-S)q_2 + (2O-T-S)(T-R) - (T-S)(2R-T-S)]q_4 - (T-S)(R-O)(1-q_2). \end{cases} \tag{173}$$

Further, we obtain

$$\tfrac{ds_X}{d\chi} = \tfrac{(T-S)(R-O)g_A(\chi)}{f_A^2(\chi)}, \tag{174}$$

where $g_A(\chi) = e_2\chi^2 + e_1\chi + e_0$ with e_i's being functions of q_2 and q_4. We show that $g_A(1) = e_2 + e_1 + e_0 \leq 0$ is the necessary and sufficient condition for s_X to be monotonically decreasing with respect to χ.

Routine calculation gives

$$g_A(1) = e_2 + e_1 + e_0 = 2(T-S)^2 q_2 q_4 \Gamma(q_4), \tag{175}$$

of which the last factor on the right-hand side is

$$\Gamma(q_4) = 2[(R-S)q_2 - (2R-T-S)]q_4 - (2R-T-S)(1-q_2). \tag{176}$$

If $q_2 = 0$ or $q_4 = 0$ (that is, $q_3 = q_4 = 0$), we immediately have $ds_X/d\chi < 0$. Otherwise, the sign of $ds_X/d\chi$ is decided by $g_A(\chi)$ and $g_A(1) \leq 0$ is a necessary condition for s_X to be a decreasing function of χ. It suffices to show that $g_A(1) \leq 0$, or equivalently, $\Gamma(q_4) \leq 0$ is also a sufficient condition.

Similarly, we let

$$2e_2 + e_1 = 4(T-S)q_2 q_4 \gamma(q_4), \tag{177}$$

of which the last factor on the right-hand side is

$$\gamma(q_4) = [(T-S)(R-S)q_2 - (T-S)(2R-T-S) - (T-R)(2O-T-S)]q_4 - (T-S)(R+O-T-S)(1-q_2). \quad (178)$$

Notice that

$$(T-O)\Gamma - \gamma = (R-S)(2O-T-S)(1-q_2)(1+q_4) > 0. \quad (179)$$

We get $\gamma < 0$.

On the other hand, since

$$\tfrac{2(T-O)^2 q_2 q_4 \Gamma - e_2}{(2O-T-S)(1-q_2)(1+q_4)} = (*)q_4 + (**) > 0, \quad (180)$$

where

$$\begin{cases} (*) = (R-S)[2(T-O)+T-S]q_2 + (T-S)(2R-T-S) - (T-R)(2O-T-S), \\ (**) = (T-S)(R-O)(1-q_2), \end{cases} \quad (181)$$

we have $e_2 < 0$.

To conclude, $g_A(1) \leq 0$ always implies $e_2 < 0$ and $2e_2 + e_1 < 0$. As a result, we have $g_A(\chi) < 0$ for $\chi > 1$. The set of q_2 and q_4 is therefore:

$$0 \leq q_4 \leq \begin{cases} 1, & 0 \leq q_2 < \frac{3(2R-T-S)}{4R-T-3S} \\ \frac{(1-q_2)(2R-T-S)}{2[(R-S)q_2-(2R-T-S)]}. & \frac{3(2R-T-S)}{4R-T-3S} \leq q_2 < 1 \end{cases} \quad (182)$$

It is worth pointing out that for a general O between P and R, this set of unbending strategies is essentially the same as that for $O = P$ (that is, when player X uses an extortionate ZD strategy).

17.2 Case III: $T + S < 2P$

For a general IPD game where $T + S < 2P$, we obtain (once again)

$$ds_X/d\chi = \tfrac{(T-S)(R-O)g_A(\chi)}{f_A^2(\chi)}, \quad (183)$$

where $g_A(\chi) = e_2\chi^2 + e_1\chi + e_0$ with e_i's being functions of q_2 and q_4. We show that (once again) $g_A(1) = e_2 + e_1 + e_0 \leq 0$ is the necessary and sufficient condition for s_X to be monotonically decreasing with respect to χ.

As before, let

$$g_A(1) = (T-S)^2 q_2 q_4 \Gamma(q_4), \quad (184)$$

of which the last factor on the right-hand side is

$$\Gamma(q_4) = [(R-S)q_2 - (2R-T-S)]q_4 - (R-P)(1-q_2). \quad (185)$$

If $q_2 = 0$ or $q_4 = 0$ (that is, $q_3 = q_4 = 0$), we immediately have $ds_X/d\chi < 0$. Otherwise, the sign of $ds_X/d\chi$ is decided by $g_A(\chi)$ and $g_A(1) \leq 0$ is a necessary condition for s_X to decrease. It suffices to show that $g_A(1) \leq 0$, or equivalently, $\Gamma(q_4)$ is also a sufficient condition.

Similarly, we let

$$2e_2 + e_1 = 2(T-S)q_2q_4\gamma(q_4), \tag{186}$$

of which the last factor on the right-hand side is

$$\gamma(q_4) = [(R-S)(P-S)q_2 - (T-S)(R+P-T-S) - (T-R)(O-P)]q_4 - [(T-S)(R-P) - (R-O)(P-S)](1-q_2). \tag{187}$$

Notice that

$$(T-O)\Gamma - \gamma = (R-S)(1-q_2)[(O+P-T-S)q_4 + O-P] > 0. \tag{188}$$

We get $\gamma < 0$.

On the other hand, since

$$\tfrac{(T-O)^2 q_2 q_4 \Gamma - e_2}{(1-q_2)[(O+P-T-S)q_4+O-P]} = (*)q_4 + (**) > 0, \tag{189}$$

where

$$\begin{cases} (*) = (R-S)(T+P-O-S)q_2 + (T-S)(R-P) - (T-R)(O-P), \\ (**) = (R-O)(P-S)(1-q_2), \end{cases} \tag{190}$$

we have $e_2 < 0$.

To conclude, $g_A(1) \leq 0$ always implies $e_2 < 0$ and $2e_2 + e_1 < 0$. As a result, we have $g_A(\chi) < 0$ for $\chi > 1$. The set of q_2 and q_4 is therefore:

$$\begin{cases} 0 \leq q_2 < 1, \\ 0 \leq q_4 \leq h_A(q_2), \end{cases} \tag{191}$$

where

$$h_A(q_2) = \begin{cases} 1, & 0 \leq q_2 < \frac{(R-P)+(2R-T-S)}{2R-P-S} \\ \frac{(R-P)(1-q_2)}{(R-S)q_2-(2R-T-S)}. & \frac{(R-P)+(2R-T-S)}{2R-P-S} \leq q_2 < 1 \end{cases} \tag{192}$$

Once again, for a general O between P and R, this set of unbending strategies is essentially the same as that for $O = P$ (that is, when player X uses an extortionate ZD strategy).

18 Class D of unbending strategies (continued)

The last class requires

$$q_4 = h_D(q_1, q_2, q_3) = \tfrac{a_{D00}}{2R-T-S}, \tag{193}$$

under which we obtain

$$\begin{cases} s_X = \dfrac{(T-S)a_{D0}\chi}{f_D(\chi)} + P, \\ \dfrac{\partial s_X}{\partial O} = \dfrac{-d_{D0}(\chi-1)}{f_D(\chi)}, \\ \dfrac{\partial s_X}{\partial \chi} = \dfrac{(T-S)a_{D0}d_{D0}}{f_D^2(\chi)}. \end{cases} \tag{194}$$

Here, $f_D(\chi) = d_{D1}\chi + d_{D0}$ and

$$\begin{cases} a_{D00} = -(T+S-2P)q_1 + (R-P)(q_2+q_3) + T + S - R - P, \\ a_{D0} = -(T+S-2O)q_1 + (R-O)(q_2+q_3) + T + S - R - O, \\ d_{D1} = -(T-S)q_1 + (T-R)q_2 + (R-S)q_3 + R - S, \\ d_{D0} = -(T-S)q_1 + (R-S)q_2 + (T-R)q_3 + T - R. \end{cases} \tag{195}$$

To determine the monotonicity of s_X, we need to figure out the sign of $a_{D0}d_{D0}$. For the set of unbending strategies, we need $a_{D0}d_{D0} < 0$. That is, $a_{D0} < 0 < d_{D0}$ or $d_{D0} < 0 < a_{D0}$. Combined with the fact that $0 \leq q_4 \leq 1$, or equivalently, $0 \leq a_{D00} \leq 2R - T - S$, the set of unbending strategies is

$$a_{D0} < 0 < d_{D0} \quad \text{or} \quad d_{D0} < 0 < a_{D0} \quad \text{and} \quad 0 \leq a_{D00} \leq 2R - T - S. \tag{196}$$

We are more interested in the subset (which we consider as Class D)

$$d_{D0} < 0 < a_{D0} \quad \text{and} \quad 0 \leq a_{D00} \leq 2R - T - S, \tag{197}$$

which can be simplified as $d_{D0} < 0 < a_{D0}$. The proof is given below.

On the one hand, it can be shown that $a_{D00} \geq a_{D0}$. Since $a_{D00} - a_{D0} = (O-P)(q_2+q_3+1-2q_1)$, if there exist such a point (p_1, p_2, p_3) that $a_{D0} > a_{D00}$, then $q_1 > (q_2 + q_3 + 1)/2$. On the other hand, a_0 evaluated at the point is $(2R - T - S)(q_2 + q_3 - 1)/2$. As $a_0 > 0$, we get $q_2 + q_3 > 1$ and hence $q_1 > 1$, a contradiction. Therefore, $a_{D00} \geq a_{D0}$ always holds and the plane $a_{D00} = 0$ is never above the plane $a_{D0} = 0$ inside the unit cube.

On the other hand, we have shown previously that if $d_{D0} < 0 < a_{D00}$, we always have $a_{D00} \leq 2R - T - S$. Therefore, for Class D of unbending strategies, the implicit condition for s_X to be a decreasing function with respect to χ is $d_{D0} < 0 < a_{D0}$.

Again, we point out that Class D is actually the set of general ZD strategies with baseline payoff O' satisfying $P < O' \leq R$. For a general IPD game, assume that player X and player Y use two ZD strategies with baseline payoffs O and O', respectively. A conclusion can be drawn intuitively.

(i) If $O' > O$, it is better off for player X to increase O and decrease χ.

(ii) If $O' < O$, it is better off for player X to increase O and increase χ.

19 Arms race of adaptive learning dynamics between unbending players and their co-players

In the steering learning dynamics, we have focused on an adaptive payoff-maximizing player against a fixed unbending co-player in various scenarios that directly complement the original study by Press and Dyson, where they assume an evolutionary adaptive player against a fixed extortionate ZD co-player. Previous experiments on IPD behavior with human participants have shown that although players often try to discourage extortionate behavior by refusing to comply with unfair demands, some players who consistently refuse to be extorted may give up on punishing extortionists if they realize that the behavior of their co-player is unlikely to change. This can occur, for example, when extortionists are pre-programmed computer agents or when they are incentivized to win an advantage, making it difficult to discipline them effectively.

To account for this tug-of-war situation in the adaptive dynamics of behavior response, we introduce a relative time scale ω that governs the time evolution of the behavioral change of an unbending player as compared to that of their co-player. To illustrate the co-adaptive dynamics of this sort, we focus on unbending players Y using class A (in the general form of $[1, q_2, 0, q_4]$) or class D (in the particular form of a general ZD) in IPD games with the conventional payoff structure, without any loss of generality. Also for ease of visualization, we consider the donation games in which reactive strategies, if employed by either parties X and Y (i.e., $[q_1, q_2, q_1, q_2]$) are in fact a subset of ZD strategies.

Specifically, the co-adaptive dynamics between a ZD player X ($\boldsymbol{p} = [p_1, p_2, p_1, p_2]$) and an unbending player Y from class A (starting from a prescribed $\boldsymbol{q} = [1, q_2, 0, q_4]$) can be described by the following system of differential equations:

$$\begin{cases} \dfrac{dp_1}{dt} = (1-\omega)\dfrac{\partial s_X(\boldsymbol{p}, \boldsymbol{q})}{\partial p_1}, \\ \dfrac{dp_2}{dt} = (1-\omega)\dfrac{\partial s_X(\boldsymbol{p}, \boldsymbol{q})}{\partial p_2}, \\ \dfrac{dq_2}{dt} = \omega\dfrac{\partial s_Y(\boldsymbol{p}, \boldsymbol{q})}{\partial q_2}, \\ \dfrac{dq_4}{dt} = \omega\dfrac{\partial s_Y(\boldsymbol{p}, \boldsymbol{q})}{\partial q_4}. \end{cases} \tag{198}$$

Similarly, the co-adaptive dynamics between a ZD player X ($\boldsymbol{p} = [p_1, p_2, p_1, p_2]$) and an unbending player Y from class D (starting from a general ZD, $\boldsymbol{q} = [q_1, q_2, q_1, q_2]$) can be given by:

$$\begin{cases} \dfrac{dp_1}{dt} = (1-\omega)\dfrac{\partial s_X(\boldsymbol{p}, \boldsymbol{q})}{\partial p_1}, \\ \dfrac{dp_2}{dt} = (1-\omega)\dfrac{\partial s_X(\boldsymbol{p}, \boldsymbol{q})}{\partial p_2}, \\ \dfrac{dq_1}{dt} = \omega\dfrac{\partial s_Y(\boldsymbol{p}, \boldsymbol{q})}{\partial q_1}, \\ \dfrac{dq_2}{dt} = \omega\dfrac{\partial s_Y(\boldsymbol{p}, \boldsymbol{q})}{\partial q_2}. \end{cases} \tag{199}$$

Intuitively, as $\omega \to 0$, the resulting dynamics reverts to our above original steering learning scenario in which an unbending player is fixed in their behavior, while for large values of ω (i.e., $\omega \to 1$), the dynamics converges to the scenario studied by Press and Dyson, which features a fixed ZD player. For intermediate values of ω, an intriguing arms race emerges between the two adaptive players, reminiscent of Red Queen dynamics. Two sets of examples are given in Figure S22 for the same extortionate player with extortion factor $\chi = 2.8$ and two different unbending players from class A and class D, respectively.

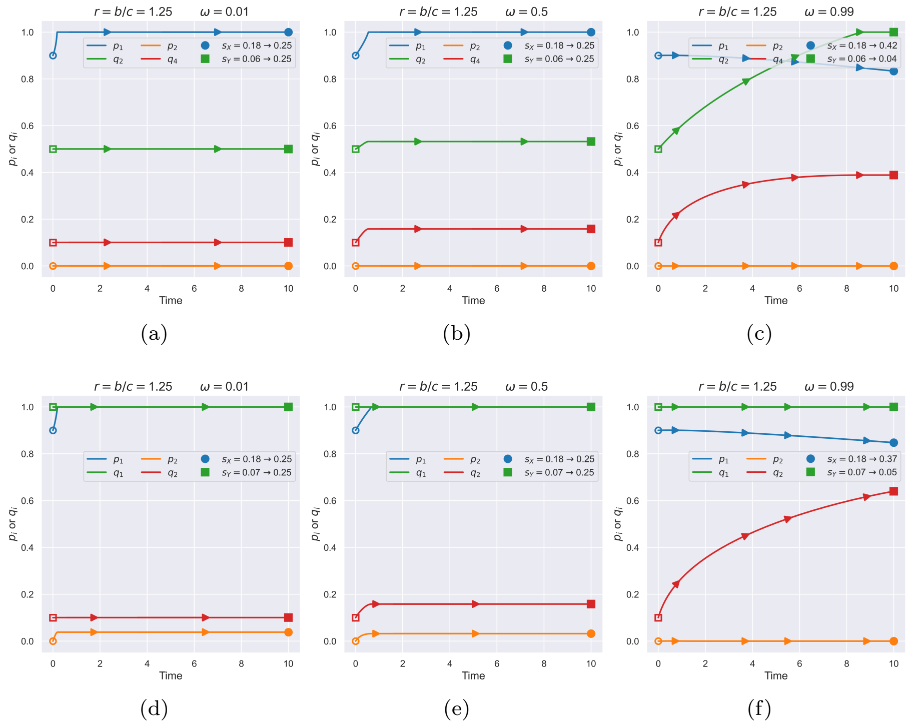

Figure S22: Co-adaptive learning dynamics of a ZD player and their unbending co-players from class A or class D. The learning curves of (a) - (c) p_1, p_2, q_2, and q_4 and (e) - (f) p_1, p_2, q_1, and q_2 are given with respect to time. The circle and the square stand for player X and player Y, the empty and the solid points represent the initial and the final states, and the arrows indicate the direction. Different values of ω between 0 and 1 are considered. The initial and the final payoffs of the two players (rounded to 2 decimal places) are also shown. As before, $r = b/c$ is the benefit-to-cost ratio in the donation games.

20 Beyond pairwise interactions: evolutionary stability of unbending strategies in population dynamics settings

So far, we have investigated the characteristics of unbending strategies and their steering role in adaptive dynamics based on pairwise interactions, which involve two parties: a focal player and their co-player. However, in order to better understand the evolutionary dynamics of unbending strategies, it is important to investigate their evolution in the context of finite populations consisting of multiple individuals (population size $N \geq 2$) using a variety of prescribed IPD strategies. To accomplish this, we typically construct a "meta" 2×2 payoff matrix based on the average payoffs obtained from the underlying IPD games, which serves to characterize the pairwise interactions between any two randomly chosen IPD strategies. If we include m different IPD strategies in our analysis, the resulting payoff matrix will have an overall dimension of $m \times m$. For instance, we can express the payoff matrix for game interactions between an unbending strategy (UB) and another extortionate ZD strategy as follows

$$\begin{array}{c} \\ \text{UB} \\ \text{ZD} \end{array} \begin{array}{c} \begin{array}{cc} \text{UB} & \text{ZD} \end{array} \\ \begin{bmatrix} a_{11} & a_{12} \\ a_{21} & a_{22} \end{bmatrix} \end{array}. \tag{200}$$

In this case, it is possible for the latter to ensure a higher payoff ($a_{21} > a_{12}$) (assuming we have $T + S > 2P$, as in conventional PD games). However, the presence of an unbending strategy can lead to a situation where $a_{11} > a_{22}$ if mutual cooperation is established among the unbending strategies (i.e., $a_{11} = R$) while the extortionate ZD strategies fail to cooperate with each other (i.e., $a_{22} = P$). Consequently, the evolutionary stability and success of unbending strategies largely depend on their ability of self-cooperation. Unbending strategies from class A ($[1, q_2, 0, q_4]$) can achieve mutual cooperation among themselves. On the other hand, the ability of unbending strategies from class D to form mutual cooperation depends on their level of generosity ($P < O \leq R$). Their self-cooperation level declines as O decreases, with unbending generous ZD players ($O = R$) being capable of mutual cooperation. In the conventional IPD game where the unbending strategy is the PSO Gambler ($[1, 0.5217, 0, 0.1205]$) from class A and the extortionate ZD strategy is the so-called ZD-Extort-2 ($[8/9, 1/2, 1/3, 0]$ with $\chi = 2$), Equation 200 becomes

$$\begin{array}{c} \\ \text{PSO Gambler} \\ \text{ZD-Extort-2} \end{array} \begin{array}{c} \begin{array}{cc} \text{PSO Gambler} & \text{ZD-Extort-2} \end{array} \\ \begin{bmatrix} 3 & 1.497 \\ 1.994 & 1 \end{bmatrix} \end{array}. \tag{201}$$

Assume a well-mixed population where each individual is equally likely to interact with anyone else in the population (excluding self-interactions). We use the average payoff π_i accrued by an individual i using an IPD strategy and interacting with the others to calculate the corresponding fitness, $f_i = \exp(\beta \pi_i)$. Here, $\beta > 0$ is the selection strength. We have $\beta \ll 1$ for weak selection and

$\beta \gg 1$ for strong selection instead. Furthermore, we use the Moran process (birth-death process) for evolutionary updating: an individual i is chosen with a probability proportional to their fitness and an offspring is produced to replace another individual randomly chosen from the entire population (including their parent). With probability μ, the offspring mutates and selects a random strategy from the set of m prescribed IPD strategies, and otherwise with probability $1-\mu$ the offspring is identical to the parental strategy. We consider the long-term abundance of IPD strategies under the limit of rare mutations ($\mu \to 0$). In this limit, the system spends most of the time on homogeneous population states and the fate of a mutant will be determined, either reaching fixation or going extinct, before the next mutant arises [10]. Figure S23 shows results from different population sizes. We find that a smaller population actually could favor extortionate ZD strategies, but their advantages would lose in a larger population. Overall, unbending strategies are favored by natural selection.

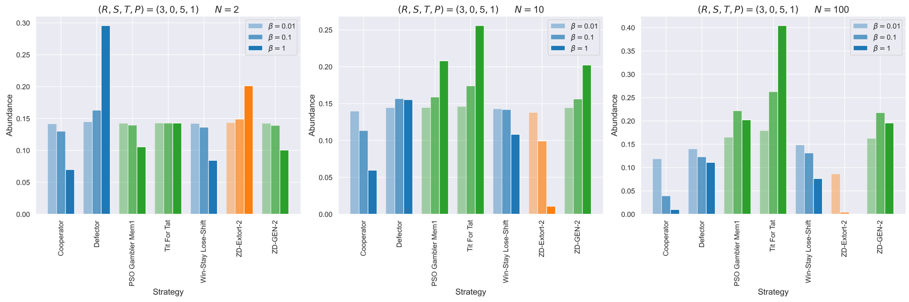

(a) small size (b) medium size (c) large size

Figure S23: Stationary distributions of the abundances in a population containing 7 memory-one IPD strategies under the limit of rare mutations with varying population sizes. The only extortionate ZD strategy is colored in orange, the unbending strategies from class A and class D and the fair ZD strategy, Tit-for-Tat, are colored in green, and the rest three strategies are colored in blue. Different transparency levels indicate different values of the selection strength β. The conventional IPD game is considered where we have $(R, S, T, P) = (3, 0, 5, 1)$.

These results can be understood using the pairwise competition dynamics since the system at most has two different IPD strategies present due to the limit of rare mutations. Specifically, once again using the above case of an unbending strategy (UB) versus an extortionate ZD strategy, natural selection favors UB over ZD (in other words, the fixation probability ρ_{UB} of one single UB player in a resident population of ZDs is greater than the fixation probability ρ_{ZD} of one single ZD player in a resident population of UBs) in their pairwise competition dynamics if and only if [11]

$$(N-2)R + Na_{12} > Na_{21} + (N-2)P. \tag{202}$$

This condition actually holds for any selection strength β and not just for weak selection. For $N = 2$, ZD has an absolute advantage to dominate UB since $a_{12} < a_{21}$. However, as the population size becomes larger, it is possible that

the above condition is satisfied and thus the evolution of unbending strategies is favored, while extortionate ZD is disfavored by natural selection.

21 Noisy IPD games with implementation errors

It is important to take into account the potential impact of noise on the evolutionary performance of IPD strategies, including those unbending ones. As pointed out above, one of the crucial factors impacting evolutionary stability is their ability to maintain mutual cooperation (or overall high cooperativity) in the presence of noise. Without loss of generality, we assume that individuals may make implementation errors: with probability $1-\varepsilon$ a focal individual makes the intended move, and otherwise with probability ε it makes the opposite move. For memory-one strategies, the transition probability $\psi(p,q)$ from outcome pair $\{a_X, a_Y\}$ to $\{a_X^*, a_Y^*\}$ after one time step becomes

$$\psi(p,q) = [(1-\varepsilon)p + \varepsilon(1-p)][(1-\varepsilon)q + \varepsilon(1-q)]. \tag{203}$$

Here, p and q denote the focal player's probability of choosing a_X^* and its co-player's probability of choosing a_Y^* given the current state $\{a_X, a_Y\}$, respectively. Taking a concrete example, we give the transition matrix for state changes between $\{\mathrm{CC}, \mathrm{CD}, \mathrm{DC}, \mathrm{DD}\}$ when the two players use the same unbending strategy from class A ($[1, q_2, 0, q_4]$) as follows

$$\begin{bmatrix} \psi(1,1) & \psi(1,0) & \psi(0,1) & \psi(0,0) \\ \psi(q_2,0) & \psi(q_2,1) & \psi(1-q_2,0) & \psi(1-q_2,1) \\ \psi(0,q_2) & \psi(0,1-q_2) & \psi(1,q_2) & \psi(1,1-q_2) \\ \psi(q_4,q_4) & \psi(q_4,1-q_4) & \psi(1-q_4,q_4) & \psi(1-q_4,1-q_4) \end{bmatrix}. \tag{204}$$

To be more specific, we have

$$\begin{cases} \psi(1,1) = (1-\varepsilon)^2, \\ \psi(1,0) = \psi(0,1) = (1-\varepsilon)\varepsilon, \\ \psi(0,0) = \varepsilon^2, \end{cases} \tag{205}$$

$$\begin{cases} \psi(q_2,0) = \psi(0,q_2) = [(1-2\varepsilon)q_2 + \varepsilon]\varepsilon, \\ \psi(q_2,1) = \psi(1,q_2) = [(1-2\varepsilon)q_2 + \varepsilon](1-\varepsilon), \\ \psi(1-q_2,0) = \psi(0,1-q_2) = [1-\varepsilon-(1-2\varepsilon)q_2]\varepsilon, \\ \psi(1-q_2,1) = \psi(1,1-q_2) = [1-\varepsilon-(1-2\varepsilon)q_2](1-\varepsilon), \end{cases} \tag{206}$$

and

$$\begin{cases} \psi(q_4,q_4) = [(1-2\varepsilon)q_4 + \varepsilon]^2, \\ \psi(q_4,1-q_4) = \psi(1-q_4,q_4) = [(1-2\varepsilon)q_4 + \varepsilon][1-\varepsilon-(1-2\varepsilon)q_4], \\ \psi(1-q_4,1-q_4) = [1-\varepsilon-(1-2\varepsilon)q_4]^2. \end{cases} \tag{207}$$

By calculating the left eigenvector of this transition matrix corresponding to the eigenvalue one, we obtain the stationary distribution of the states over

{CC, CD, DC, DD} in closed form. It is worth pointing out that the same approach can be applied to any memory-one strategies. Up to a common normalization factor $\varphi(q_2, q_4, \varepsilon)$, we can show that

$$\begin{cases} v_{\mathrm{CC}} = [(1-2\varepsilon)q_4 + \varepsilon]\{(1-2\varepsilon)^2(1-q_2)q_4 + [(1-2\varepsilon)q_2 + 1]\varepsilon\}/\varphi(q_2, q_4, \varepsilon), \\ v_{\mathrm{CD}} = v_{\mathrm{DC}} = [(1-2\varepsilon)q_4 + \varepsilon][2(1-\varepsilon) - (1-2\varepsilon)q_4]\varepsilon/\varphi(q_2, q_4, \varepsilon), \\ v_{\mathrm{DD}} = [4\varepsilon^2 - 5\varepsilon + 2 - (1-2\varepsilon)(2-3\varepsilon)q_2]\varepsilon/\varphi(q_2, q_4, \varepsilon). \end{cases} \tag{208}$$

For small levels of noise, $\varepsilon \to 0$,

$$\begin{cases} v_{\mathrm{CC}} \approx 1 - \dfrac{2[(2-q_4)q_4 + 1 - q_2]}{(1-q_2)q_4^2}\varepsilon, \\ v_{\mathrm{CD}} = v_{\mathrm{DC}} \approx \dfrac{2-q_4}{(1-q_2)q_4}\varepsilon, \\ v_{\mathrm{DD}} \approx \dfrac{2}{q_4^2}\varepsilon. \end{cases} \tag{209}$$

In Figure S24, we use a specific example, the PSO Gambler $[1, 0.5217, 0, 0.1205]$, which suggests that unbending strategies are robust to implementation errors. Similarly, we can demonstrate the robustness of class D using generous ZD strategies.

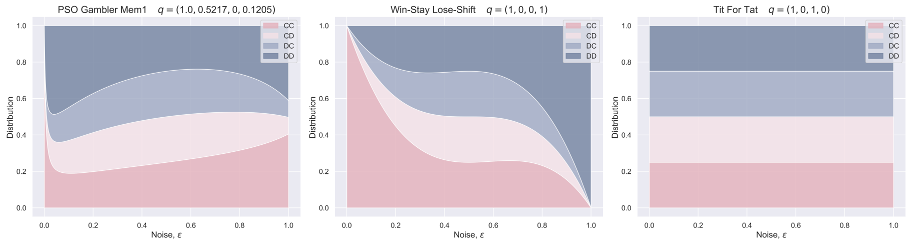

(a) (b) (c)

Figure S24: Stationary distributions of the outcomes in a match between two identical players. The conventional IPD game is considered where we have $(R, S, T, P) = (3, 0, 5, 1)$.

In contrast, the stationary distribution of states among TFT players in the presence of noise becomes

$$v_{\mathrm{CC}} = \frac{1}{4},\; v_{\mathrm{CD}} = \frac{1}{4},\; v_{\mathrm{DC}} = \frac{1}{4},\; v_{\mathrm{DD}} = \frac{1}{4}, \tag{210}$$

for any level of noise ε.

In sum, our comparative analyses indicate the robustness of unbending strategies (from class A and from class D containing generous ZD strategies) with respect to small levels of noise.

References

[1] Press, W. H. & Dyson, F. J. Iterated prisoner's dilemma contains strategies that dominate any evolutionary opponent. *Proceedings of the National Academy of Sciences* **109**, 10409–10413 (2012). URL https://www.pnas.org/content/109/26/10409. https://www.pnas.org/content/109/26/10409.full.pdf.

[2] Stewart, A. J. & Plotkin, J. B. Extortion and cooperation in the prisoner's dilemma. *Proceedings of the National Academy of Sciences* **109**, 10134–10135 (2012). URL https://www.pnas.org/content/109/26/10134. https://www.pnas.org/content/109/26/10134.full.pdf.

[3] Stewart, A. J. & Plotkin, J. B. From extortion to generosity, evolution in the iterated prisoner's dilemma. *Proceedings of the National Academy of Sciences* **110**, 15348–15353 (2013). URL https://www.pnas.org/content/110/38/15348. https://www.pnas.org/content/110/38/15348.full.pdf.

[4] Adami, C. & Hintze, A. Evolutionary instability of zero-determinant strategies demonstrates that winning is not everything. *Nature Communications* **4**, 2193 EP – (2013). URL https://doi.org/10.1038/ncomms3193.

[5] Hilbe, C., Nowak, M. A. & Sigmund, K. Evolution of extortion in iterated prisoner's dilemma games. *Proceedings of the National Academy of Sciences* **110**, 6913–6918 (2013). URL https://www.pnas.org/content/110/17/6913. https://www.pnas.org/content/110/17/6913.full.pdf.

[6] Hilbe, C., Nowak, M. A. & Traulsen, A. Adaptive dynamics of extortion and compliance. *PLOS ONE* **8**, 1–9 (2013). URL https://doi.org/10.1371/journal.pone.0077886.

[7] Hilbe, C., Traulsen, A. & Sigmund, K. Partners or rivals? strategies for the iterated prisoner's dilemma. *Games and Economic Behavior* **92**, 41 – 52 (2015). URL http://www.sciencedirect.com/science/article/pii/S0899825615000822.

[8] Hilbe, C., Wu, B., Traulsen, A. & Nowak, M. A. Evolutionary performance of zero-determinant strategies in multiplayer games. *Journal of Theoretical Biology* **374**, 115 – 124 (2015). URL http://www.sciencedirect.com/science/article/pii/S0022519315001472.

[9] Adami, C., Schossau, J. & Hintze, A. Evolutionary game theory using agent-based methods. *Physics of Life Reviews* **19**, 1 – 26 (2016). URL http://www.sciencedirect.com/science/article/pii/S1571064516300884.

[10] Fudenberg, D. & Imhof, L. A. Imitation processes with small mutations. *Journal of Economic Theory* **131**, 251–262 (2006). URL https://www.sciencedirect.com/science/article/pii/S0022053105001080.

[11] Antal, T., Nowak, M. A. & Traulsen, A. Strategy abundance in 2×2 games for arbitrary mutation rates. *Journal of Theoretical Biology* **257**, 340–344 (2009). URL https://www.sciencedirect.com/science/article/pii/S0022519308006127.

[12] Harper, M. *et al.* Reinforcement learning produces dominant strategies for the iterated prisoner's dilemma. *PLOS ONE* **12**, 1–33 (2017). URL https://doi.org/10.1371/journal.pone.0188046.

[13] Hilbe, C., Röhl, T. & Milinski, M. Extortion subdues human players but is finally punished in the prisoner's dilemma. *Nature Communications* **5**, 3976 EP – (2014). URL https://doi.org/10.1038/ncomms4976.

[14] Hilbe, C., Wu, B., Traulsen, A. & Nowak, M. A. Cooperation and control in multiplayer social dilemmas. *Proceedings of the National Academy of Sciences* **111**, 16425–16430 (2014). URL https://www.pnas.org/content/111/46/16425. https://www.pnas.org/content/111/46/16425.full.pdf.

[15] Chen, J. & Zinger, A. The robustness of zero-determinant strategies in iterated prisoner's dilemma games. *Journal of Theoretical Biology* **357**, 46 – 54 (2014). URL http://www.sciencedirect.com/science/article/pii/S0022519314002744.
